# Application of non-HDL cholesterol for population-based cardiovascular risk stratification: results from the Multinational Cardiovascular Risk Consortium

**DOI:** 10.1016/S0140-6736(19)32519-X

**Published:** 2019-12-14

**Authors:** Fabian J Brunner, Christoph Waldeyer, Francisco Ojeda, Veikko Salomaa, Frank Kee, Susana Sans, Barbara Thorand, Simona Giampaoli, Paolo Brambilla, Hugh Tunstall-Pedoe, Marie Moitry, Licia Iacoviello, Giovanni Veronesi, Guido Grassi, Ellisiv B Mathiesen, Stefan Söderberg, Allan Linneberg, Hermann Brenner, Philippe Amouyel, Jean Ferrières, Abdonas Tamosiunas, Yuriy P Nikitin, Wojciech Drygas, Olle Melander, Karl-Heinz Jöckel, David M Leistner, Jonathan E Shaw, Demosthenes B Panagiotakos, Leon A Simons, Maryam Kavousi, Ramachandran S Vasan, Robin P F Dullaart, S Goya Wannamethee, Ulf Risérus, Steven Shea, James A de Lemos, Torbjørn Omland, Kari Kuulasmaa, Ulf Landmesser, Stefan Blankenberg, Multinational Cardiovascular Risk Consortium, Tanja Zeller, Jukka Kontto, Satu Männistö, Andres Metspalu, Karl Lackner, Philipp Wild, Annette Peters, Christa Meisinger, Chiara Donfrancesco, Stefano G. Signorini, Maris Alver, Mark Woodward, Francesco Gianfagna, Simona Costanzo, Tom Wilsgaard, Mats Eliasson, Torben Jørgensen, Henry Völzke, Marcus Dörr, Matthias Nauck, Ben Schöttker, Thiess Lorenz, Nataliya Makarova, Raphael Twerenbold, Jean Dallongeville, Annette Dobson, Sofia Malyutina, Andrzej Pajak, Gunnar Engström, Martin Bobak, Börge Schmidt, Tuija Jääskeläinen, Teemu Niiranen, Pekka Jousilahti, Graham Giles, Allison Hodge, Jens Klotsche, Dianna J. Magliano, Magnus N. Lyngbakken, Kristian Hveem, Christos Pitsavos, Emelia J. Benjamin, Stephan J.L. Bakker, Peter Whincup, M. Kamran Ikram, Martin Ingelsson, Wolfgang Koenig

**Affiliations:** a University Heart & Vascular Center Hamburg, Department of Cardiology, Hamburg, Germany; b National Institute for Health and Welfare, Helsinki, Finland; c Centre for Public Health, Queens University of Belfast, Belfast, UK; d Catalan Department of Health, Barcelona, Spain; e Institute of Epidemiology, Helmholtz Zentrum München, German Research Center for Environmental Health, Neuherberg, Germany; f Department of Cardiovascular, Endocrine-metabolic Diseases, and Ageing, National Institutes of Health-ISS, Rome, Italy; g Department of Medicine and Surgery, University of Milano-Bicocca, Milan, Italy; h Cardiovascular Epidemiology Unit, Institute of Cardiovascular Research, University of Dundee, Dundee, UK; i Department of Epidemiology and Public health, University Hospital of Strasbourg, Strasbourg, France; j Department of Epidemiology and Prevention, IRCCS Neuromed, Pozzilli, Italy; k Research Center in Epidemiology and Preventive Medicine, Department of Medicine and Surgery, University of Insubria, Varese, Italy; l Clinica Medica, Department of Medicine and Surgery, University of Milano Bicocca, Milan, Italy; m Department of Clinical Medicine, University of Tromsø—The Arctic University of Tromsø, Tromsø, Norway; n Department of Neurology and Neurophysiology, University Hospital of North Norway, Tromsø, Norway; o Department of Public Health and Clinical Medicine, and Heart Center, Cardiology, Umeå University, Umeå, Sweden; p Center for Clinical Research and Prevention, Bispebjerg and Frederiksberg Hospital, Copenhagen, Denmark; q Department of Clinical Medicine, Faculty of Health and Medical Sciences, University of Copenhagen, Copenhagen, Denmark; r Division of Clinical Epidemiology and Aging Research, German Cancer Research Center, Heidelberg, Germany; s Risk Factors and Molecular Determinants of Aging Diseases, University of Lille, Lille, France; t Inserm, Lille, France; u Centre Hospitalier Universitaire de Lille, Lille, France; v Institut Pasteur de Lille, Lille, France; w Toulouse University School of Medicine, Toulouse, France; x Institute of Cardiology, Lithuanian University of Health Sciences, Kaunas, Lithuania; y Research Institute of Internal and Preventive Medicine, Branch of Federal Research Center, Institute of Cytology and Genetics, Siberian Branch, Russian Academy of Sciences, Novosibirsk, Russia; z Department of Epidemiology, Cardiovascular Disease Prevention and Health Promotion, National Institute of Cardiology, Warsaw, Poland; aa Department of Clinical Sciences, Malmö, Lund University, Malmö, Sweden; ab Institute for Medical Informatics, Biometry and Epidemiology, University Hospital of Essen, Essen, Germany; ac Department of Cardiology, Charité Berlin—University Medicine, Campus Benjamin Franklin, Berlin, Germany; ad German Centre for Cardiovascular Research, Partner Site Berlin, Berlin, Germany; ae Berlin Institute of Health, Berlin, Germany; af Baker Heart and Diabetes Institute, Melbourne, VIC, Australia; ag Department of Nutrition and Dietetics, School of Health Science and Education, Harokopio University, Athens, Greece; ah University of New South Wales, Sydney, NSW, Australia; ai St Vincent's Hospital, Sydney, NSW, Australia; aj Department of Epidemiology, Erasmus Medical Center, University Medical Center Rotterdam, Rotterdam, Netherlands; ak Boston University and the National Heart, Lung, and Blood Institute's Framingham Study, Framingham, MA, USA; al Department of Endocrinology, University Medical Center Groningen, University of Groningen, Groningen, Netherlands; am Department of Primary Care and Population Health, University College London, London, UK; an Department of Public Health and Caring Sciences, Clinical Nutrition and Metabolism, Uppsala University, Uppsala, Sweden; ao Departments of Medicine and Epidemiology, Columbia University, New York, NY, USA; ap Division of Cardiology, University of Texas Southwestern Medical Center, Dallas, TX, USA; aq Department of Cardiology, Division of Medicine, Akershus University Hospital, Lørenskog, Norway; ar Center for Heart Failure Research, Institute of Clinical Medicine, University of Oslo, Oslo, Norway; as German Center for Cardiovascular Research, Partner Site Hamburg/Kiel/Lübeck, Hamburg, Germany

## Abstract

**Background:**

The relevance of blood lipid concentrations to long-term incidence of cardiovascular disease and the relevance of lipid-lowering therapy for cardiovascular disease outcomes is unclear. We investigated the cardiovascular disease risk associated with the full spectrum of bloodstream non-HDL cholesterol concentrations. We also created an easy-to-use tool to estimate the long-term probabilities for a cardiovascular disease event associated with non-HDL cholesterol and modelled its risk reduction by lipid-lowering treatment.

**Methods:**

In this risk-evaluation and risk-modelling study, we used Multinational Cardiovascular Risk Consortium data from 19 countries across Europe, Australia, and North America. Individuals without prevalent cardiovascular disease at baseline and with robust available data on cardiovascular disease outcomes were included. The primary composite endpoint of atherosclerotic cardiovascular disease was defined as the occurrence of the coronary heart disease event or ischaemic stroke. Sex-specific multivariable analyses were computed using non-HDL cholesterol categories according to the European guideline thresholds, adjusted for age, sex, cohort, and classical modifiable cardiovascular risk factors. In a derivation and validation design, we created a tool to estimate the probabilities of a cardiovascular disease event by the age of 75 years, dependent on age, sex, and risk factors, and the associated modelled risk reduction, assuming a 50% reduction of non-HDL cholesterol.

**Findings:**

Of the 524 444 individuals in the 44 cohorts in the Consortium database, we identified 398 846 individuals belonging to 38 cohorts (184 055 [48·7%] women; median age 51·0 years [IQR 40·7–59·7]). 199 415 individuals were included in the derivation cohort (91 786 [48·4%] women) and 199 431 (92 269 [49·1%] women) in the validation cohort. During a maximum follow-up of 43·6 years (median 13·5 years, IQR 7·0–20·1), 54 542 cardiovascular endpoints occurred. Incidence curve analyses showed progressively higher 30-year cardiovascular disease event-rates for increasing non-HDL cholesterol categories (from 7·7% for non-HDL cholesterol <2·6 mmol/L to 33·7% for ≥5·7 mmol/L in women and from 12·8% to 43·6% in men; p<0·0001). Multivariable adjusted Cox models with non-HDL cholesterol lower than 2·6 mmol/L as reference showed an increase in the association between non-HDL cholesterol concentration and cardiovascular disease for both sexes (from hazard ratio 1·1, 95% CI 1·0–1·3 for non-HDL cholesterol 2·6 to <3·7 mmol/L to 1·9, 1·6–2·2 for ≥5·7 mmol/L in women and from 1·1, 1·0–1·3 to 2·3, 2·0–2·5 in men). The derived tool allowed the estimation of cardiovascular disease event probabilities specific for non-HDL cholesterol with high comparability between the derivation and validation cohorts as reflected by smooth calibration curves analyses and a root mean square error lower than 1% for the estimated probabilities of cardiovascular disease. A 50% reduction of non-HDL cholesterol concentrations was associated with reduced risk of a cardiovascular disease event by the age of 75 years, and this risk reduction was greater the earlier cholesterol concentrations were reduced.

**Interpretation:**

Non-HDL cholesterol concentrations in blood are strongly associated with long-term risk of atherosclerotic cardiovascular disease. We provide a simple tool for individual long-term risk assessment and the potential benefit of early lipid-lowering intervention. These data could be useful for physician–patient communication about primary prevention strategies.

**Funding:**

EU Framework Programme, UK Medical Research Council, and German Centre for Cardiovascular Research.

**Research in context**

**Evidence before this study**

The blood concentrations of non-HDL and LDL cholesterol are accepted as causal cardiovascular risk factors and constitute a cornerstone of cardiovascular disease risk prediction in the general population. The benefit of lipid-lowering interventions for cardiovascular disease risk reduction is proven by mendelian randomisation studies, prospective epidemiological cohort studies, and randomised trials. For the present study, we assessed recommendations made by the European and US guidelines and their underlying references for lipid-lowering strategies in primary prevention. Furthermore, we reviewed the cardiovascular risk calculators as recommended by these guidelines (Systematic COronary Risk Evaluation [SCORE] and Pooled Cohort Equations). We found very little data describing the effect of baseline non-HDL and LDL cholesterol on long-term or even lifetime outcomes. Cardiovascular risk assessment was mainly based on 10-year medium-term follow-up. All identified randomised trials for lipid-lowering intervention in cardiovascular primary prevention did not exceed a follow-up of 7 years and, moreover, especially young individuals were underrepresented in those studies.

**Added value of this study**

To our knowledge, our study provides the most comprehensive analysis of long-term risk for cardiovascular disease related to non-HDL cholesterol and offers an easily applicable tool to assess the long-term probabilities for cardiovascular disease events associated with non-HDL cholesterol. On the basis of a derivation and validation approach to risk prediction, our data provide robust multinational information. By using an up-to-date multinational population-based pooled cohort dataset, we provide a model that calculates the potential benefit of an early lipid-lowering strategy in individuals without prevalent cardiovascular disease across a range of non-HDL cholesterol categories.

**Implications of all the available evidence**

There is evidence for the positive outcomes in lipid-lowering interventions in individuals with cardiovascular disease or those with very high cholesterol concentrations. Our data extend current knowledge and highlight the impact of non-HDL cholesterol on very long-term cardiovascular disease outcomes. A simulation of early reduction of non-HDL cholesterol across a range of lipid concentration categories estimates the risk reduction in the general population. Meeting targets for global reduction of cardiovascular disease morbidity and mortality will require increased awareness of the value of early cholesterol determination and careful evaluation of potential strategies for reduction of lipid concentrations across the lifespan.

## Introduction

Numerous studies have provided consistent evidence for a causal relationship between blood cholesterol concentrations and cardiovascular disease.^1^, ^2^ Calculating the concentration of non-HDL cholesterol offers a simple way to analyse the total amount of proatherogenic lipoproteins containing apolipoprotein B (apoB).^3^ Such proteins include very low-density lipoproteins and their metabolic remnants, intermediate-density lipoproteins, lipoprotein(a), and low-density lipoproteins.^3^ Besides estimating cholesterol concentrations contained in LDL particles (LDL cholesterol), the assessment of non-HDL cholesterol calculated as total cholesterol minus HDL cholesterol is therefore recommended by current US and European guidelines for cardiovascular risk estimation.^4^, ^5^ The indication for lipid-lowering therapy to prevent cardiovascular disease events in high-risk individuals or for secondary prevention is unequivocal.^4^, ^5^, ^6^, ^7^, ^8^, ^9^ However, the decision on implementing a lipid-lowering intervention in the primary prevention setting is a major challenge in clinical practice for several reasons. Data on the association between the concentrations of the entire range of bloodstream lipids and very long-term cardiovascular outcomes in the general population are rather sparse.^10^, ^11^ In addition, conventional primary prevention guidelines recommend lipid-lowering intervention on the basis of lipid concentration thresholds and the person's individual 10-year cardiovascular risk.^4^, ^5^ This risk assessment might underestimate or fail to take proper account of the cumulative lifetime risk of cardiovascular disease and lipid-related cardiovascular disease risk, particularly in young adults.^12^ Furthermore, increased non-HDL cholesterol blood concentrations early in life seem to be stable over the life course and are predictive for incident cardiovascular disease.^13^ Therefore, in this study we aimed to (1) evaluate long-term risk for cardiovascular disease in the population related to non-HDL cholesterol on the basis of existing thresholds of blood lipid concentrations; (2) establish an easily applicable tool to assess the long-term probabilities for cardiovascular disease events associated with non-HDL cholesterol, using a derivation and validation approach; and (3) provide a model indicating the potential benefit of an early lipid-lowering strategy in individuals without prevalent cardiovascular disease.

## Methods

### Study design and participants

In this risk-evaluation and risk-modelling study, we used individual-level data from the Multinational Cardiovascular Risk Consortium (appendix pp 2–11). Currently, the Consortium comprises data on 524 444 individuals from 44 population-based cohorts across Europe, Australia, and the USA. These include 23 harmonised cohorts from the MONICA Risk Genetics Archiving and Monograph (MORGAM)^14^ and Biomarker for Cardiovascular Risk Assessment in Europe (BiomarCaRE)^15^ studies and another 21 harmonised, population-based cohorts with the same specifications as defined for the BiomarCaRE and MORGAM projects (appendix pp 15–18). Of these, we used cohorts that had available data on cardiovascular disease endpoints (appendix pp 16–18). We excluded data on participants with prevalent cardiovascular disease, defined as a history of myocardial infarction, coronary artery bypass grafting, percutaneous transluminal coronary angioplasty, or ischaemic or haemorrhagic stroke. Detailed information about the study cohorts including the number of individuals with available information for each variable are provided in the appendix (pp 19–27). Informed consent was obtained if necessary (depending on survey year and local requirement). Detailed information for each cohort is given in the study descriptions (appendix pp 2–11).

### Procedures

Our outcome measure was the first occurrence of a major cardiovascular event (appendix pp 16–18). This was assessed in all participating individuals and death of non-cardiovascular-disease cause was used as a competing risk if appropriate. We defined it as a composite endpoint of the first non-fatal or fatal coronary heart disease or ischaemic stroke event. Coronary heart disease was defined as a composite of non-fatal or fatal myocardial infarction including unstable angina, coronary death, and coronary revascularisation. Outcomes were measured in each prospective cohort study by standardised follow-up (visit, report, registry information, etc; details are given for each cohort, appendix pp 2–11). Composite endpoint information for current analyses is provided in the appendix (pp 16–18). There were no secondary outcomes.

We based our cholesterol threshold concentrations on the treatment-determining cholesterol categories of the 2016 European guidelines on cardiovascular disease prevention.^16^ As recommended by the guidelines, we calculated non-HDL cholesterol thresholds by adding 30 mg/dL to the LDL cholesterol thresholds defined in our cohorts. Our analyses were based on baseline information available for concentrations of non-HDL cholesterol and LDL cholesterol. We report threshold concentrations in mmol/L, assuming that 1 mmol/L contains 38·67 mg/dL of LDL cholesterol and non-HDL cholesterol (for non-HDL cholesterol, the following categories were used: <100 mg/dL [<2·6 mmol/L]; 100 to <145 mg/dL [2·6 to <3·7 mmol/L]; 145 to <185 mg/dL [3·7 to <4·8 mmol/L]; 185 to <220 mg/dL [4·8 to <5·7 mmol/L], and ≥220 mg/dL [≥5·7 mmol/L]). The cutoff between the second and third category differs slightly from the European guidelines. We adapted this cutoff from 130 mg/dL (3·4 mmol/L) to 145 mg/dL (3·7 mmol/L), since the latter is the goal for lipid-lowering therapy recommended for individuals at low risk,^4^ who comprise most of our study population.

The following study variables were considered for analysis (but were not endpoints): age, sex, year of examination, body-mass index (BMI), systolic blood pressure, smoking status, use of cholesterol-lowering medication, total cholesterol, HDL cholesterol, and non-HDL cholesterol. History of arterial hypertension, diabetes, stroke, and myocardial infarction were defined as they had been documented or self-reported. Antihypertensive medication, lipid-lowering medication, and smoking status were self-reported. For individuals receiving lipid-lowering therapy, baseline concentrations of non-HDL cholesterol and LDL cholesterol were inflated by 30% for the analyses, assuming that treatment with statins had a moderate effect on lipid reduction and was initiated late during lifetime.^17^ Information about individuals receiving lipid-lowering therapy stratified by survey decades and age is shown in the appendix (p 28).

### Statistical analysis

For each analysis, only individuals with all data available on the relevant variables were used. To investigate the association of non-HDL cholesterol concentrations and time to cardiovascular disease, sex-specific cumulative incidence curves were produced according to the defined baseline non-HDL cholesterol threshold concentrations, with death of non-cardiovascular-disease causes as competing risk, using the Aalen-Johansen estimator. Multivariable Cox proportional hazards models were then computed, stratified for cohort and sex. Non-HDL cholesterol was coded as a categorical variable, with the help of dummy variables, using our predefined thresholds and included an interaction term with sex, to obtain sex-specific associations. To examine the association of non-HDL cholesterol across different ages, the models were expanded by adding an interaction between age category (<45, 45–59, and ≥60 years at baseline) and non-HDL cholesterol. Additional Cox regression analyses were done to model the concentrations of non-HDL cholesterol as a continuous variable via cubic splines. All Cox models were adjusted for age (timescale), sex (strata), cohort (strata), and classical cardiovascular risk factors (smoking status, diabetes, BMI, systolic blood pressure, and antihypertensive medication). In a sensitivity analysis, sex-specific hazard ratios (HRs) for non-HDL cholesterol as a categorical variable were estimated in each country using models similar to the primary analysis, and the results were pooled using a random-effects multivariate meta-analysis. Further sensitivity analyses include the computation of time-dependent HRs for non-HDL cholesterol to examine deviations from the proportional hazards assumption (appendix p 29).

To establish a tool to assess the probability of cardiovascular disease by the age of 75 years, we split the whole data randomly into two parts of approximately equal size into derivation and validation datasets. In particular, each single cohort was part of both datasets. Using those observations where the required endpoint and covariate information was available, we developed a model that was calculated on the basis of cause-specific Cox models for cardiovascular disease stratified for sex and cohort. Death from non-cardiovascular-disease causes was added as a competing risk, to account for the fact that an individual dying from such a cause before developing cardiovascular disease will not be able to develop cardiovascular disease. These models used age as the timescale and the same variables as our previous Cox models. Non-HDL cholesterol, BMI, and systolic blood pressure were modelled using cubic splines. The models included an interaction between non-HDL cholesterol and sex. The model computation used the full available follow-up periods but was applied only to individuals aged between 35 and 70 years at baseline to estimate their probability of cardiovascular disease by the age of 75 years. These predicted probabilities were averaged within each combination of sex, non-HDL cholesterol category, age category, and according to whether the number of risk factors present (smoking, arterial hypertension, diabetes, and obesity) was greater than or equal to two, or less than two. We then assumed a reduction of the probabilities and their averages through a 50% or 30% reduction of non-HDL cholesterol, on the basis of the equation 1 – exp(−0·249 + [number of years of treatment–5] × [–0·0152]) for the expected proportional risk reduction per mmol/L in LDL cholesterol for a given treatment duration.^2^ It was assumed that this equation was also valid for non-HDL cholesterol. Following the methods described by Austin,^18^ these average probabilities can then be used to estimate risk differences, numbers needed to treat, and differences in relative risk.

To obtain predicted probabilities from the cause-specific Cox models for each combination of sex and cohort, the respective cause-specific baseline hazard function was estimated by fitting a Weibull curve to age and adjusting for the linear predictor of the Cox model. By using age as the timescale, it is possible to estimate the probability of an event within each combination of sex and cohort for the whole range of ages available in that part of the data. C-indices and smooth calibration curves were calculated for different timeframes separately in the derivation and validation datasets. The calibration curves were based on smoothing pseudo-values calculated with the Aalen-Johansen estimate of cumulative incidence.^19^ Tenfold cross-validation was used in these computations.

Sensitivity analyses used time-dependent coefficients that were allowed to change with attained age in the predefined groups (<45, 45–59, and ≥60 years). To calculate the effect of non-HDL cholesterol reduction on cardiovascular risk, we assumed a reduction of baseline non-HDL cholesterol by 30% or 50% (adapted from the 2018 American College of Cardiology/American Heart Association Guideline on the Management of Blood Cholesterol).^5^ On the basis of this reduction, we recalculated the probability of a cardiovascular disease event by the age of 75 years, as in our predictive model. The models and their averaged predictions were calculated in the derivation cohorts and also independently in the validation cohorts, and the discrepancy between the average predictions was compared using the root mean square error. This last step was repeated using the complete dataset, excluding each country in turn and also in the excluded country, thus generating country-specific root mean square errors. The final models and their predictions were computed in the complete dataset. Cumulative incidence curves for specific combinations of variables were produced on the basis of the cause-specific Cox models. Here, we also assumed a 50% reduction in non-HDL cholesterol for the included individuals. Applying this equation for the expected proportional risk reduction for a given treatment duration could yield a non-monotonic curve. Therefore, the resulting curves were forced to remain constant in the event of a decrease.

To compare the discrimination of a model using non-HDL cholesterol with that of a model using LDL cholesterol, additional cause-specific Cox models were computed using age as the timescale, sex and cohort as strata, adjusting for cardiovascular risk factors (smoking status, diabetes, BMI, systolic blood pressure, and antihypertensive medication), and including a sex–lipid variable interaction. All continuous variables, except age, were modelled using cubic splines. Tenfold cross-validation was used when computing the C-indices. The main analyses for non-HDL cholesterol were repeated for LDL cholesterol. All statistical methods were implemented in R statistical software, version 3.6.1.

### Role of the funding source

The funder of the study had no role in study design, data collection, data analysis, data interpretation, or writing of the report. The corresponding author (SB) and the lead statistician (FO) had full access to all the data in the study, and the corresponding author had final responsibility for the decision to submit for publication.

## Results

We identified 38 cohorts reporting on 398 846 individuals with 5 604 735 person-years of follow-up data available for analysis, of whom 184 055 (48·7%) were women (Table 1, Table 2, appendix pp 19–27, 30). Examination years ranged from 1970 to 2013 (years for each cohort are given in the appendix pp 19–27). We included 199 415 individuals in the derivation cohort (91 786 [48·4%] women) and 199 431 (92 269 [49·1%] women) in the validation cohort. The median age of participants was 51·0 years (IQR 40·7–59·7). 12 311 (4·9%) were receiving lipid-lowering therapy. Median follow-up time was 13·5 years (IQR 7·0–20·1) with a maximum follow-up of 43·6 years.

**Table 1:** Weighted baseline characteristics of the study population including derivation and validation datasets

	**Examination years**	**Examination age (years)**	**Age <45 years**	**Age 45–59 years**	**Age ≥60 years**	**Female**	**SCORE**	**PCE**	**BMI (kg/m^2^)**	**Hypertension**	**Diabetes**	**Daily smoker**
All (n=398 846)	1970–2013	51·0 (40·7–59·7)	118 731 (32·9%)	179 265 (42·8%)	100 842 (24·3%)	184 055 (48·7%)	1·0 (0·2–3·6)	4·3 (1·2–11·4)	25·7 (23·1–28·8)	162 632 (40·1%)	18 656 (4·8%)	130 940 (33·3%)
Numbers available	377 596 (95·3%)	398 838 (>99·9%)	398 838 (>99·9%)	398 838 (>99·9%)	398 838 (>99·9%)	398 846 (100·0%)	357 911 (81·4%)	277 278 (62·7%)	395 505 (99·2%)	388 941 (97·8%)	384 164 (96·5%)	387 447 (97·4%)
Derivation (n=199 415)	1970–2013	51·0 (40·9–59·7)	59 387 (32·5%)	89 676 (43·1%)	50 348 (24·4%)	91 786 (48·4%)	1·0 (0·2–3·6)	4·3 (1·2–11·4)	25·8 (23·1–28·8)	81 279 (40·3%)	9245 (4·8%)	65 640 (33·8%)
Numbers available	188 767 (95·2%)	199 411 (>99·9%)	199 411 (>99·9%)	199 411 (>99·9%)	199 411 (>99·9%)	199 415 (100·0%)	178 980 (82·0%)	138 663 (63·3%)	197 767 (99·2%)	194 534 (97·8%)	192 097 (96·4%)	193 708 (97·4%)
Validation (n=199 431)	1970–2013	50·9 (40·6–59·6)	59 344 (33·3%)	89 589 (42·5%)	50 494 (24·2%)	92 269 (49·1%)	1·0 (0·2–3·6)	4·3 (1·2–11·4)	25·7 (23·2–28·9)	81 353 (40·0%)	9411 (4·7%)	65 300 (32·8%)
Numbers available	188 829 (95·3%)	199 427 (>99·9%)	199 427 (>99·9%)	199 427 (>99·9%)	199 427 (>99·9%)	199 431 (100·0%)	178 931 (80·9%)	138 615 (62·2%)	197 738 (99·2%)	194 407 (97·8%)	192 067 (96·6%)	193 739 (97·5%)
p value	0·51	0·63	0·49	0·44	0·66	0·43	0·85	0·76	0·85	0·70	0·66	0·19

Data are n (%) for numbers available, median (IQR) for continuous variables (except for examination years where the range is given), n (%) of participants or % (95% CI) for categorical variables. p values are given for the validation cohort versus the derivation cohort using weighted versions of the Mann-Whitney and χ^2^ test. Due to the presence of the Estonian case-cohort dataset, the summary estimates (% and median [IQR]) are weighted by the inverse of the inclusion probability for individuals in that cohort. The individuals from other cohorts are given weight 1 in the computation. Totals (n) are not weighted. From the Estonian data only, the sub-cohort is used in the table computations. SCORE=Systematic Coronary Risk Estimation. PCE=Pooled Cohort Equations. BMI=body-mass index.

**Table 2:** Weighted baseline characteristics for cholesterol-related measures

	**Cholesterol-lowering medication**	**LDL cholesterol (mmol/L)**	**HDL cholesterol (mmol/L)**	**Non-HDL cholesterol (mmol/L)**	**Non-HDL cholesterol categories**				
					<2·6 mmol/L	2·6 to <3·7 mmol/L	3·7 to <4·8 mmol/L	4·8 to <5·7 mmol/L	≥5·7 mmol/L
All (n=398 846)	12 311 (4·9%)	3·6 (2·9–4·3)	1·3 (1·1–1·6)	4·3 (3·5–5·2)	16 572 (5·1%)	85 811 (26·2%)	109 270 (33·3%)	67 099 (20·4%)	49 575 (15·1%)
Numbers available	247 733 (59·7%)	264 999 (60·1%)	328 517 (74·4%)	328 327 (74·3%)	328 327 (74·3%)	328 327 (74·3%)	328 327 (74·3%)	328 327 (74·3%)	328 327 (74·3%)
Derivation (n=199 415)	6081 (4·9%)	3·6 (2·9–4·3)	1·3 (1·1–1·6)	4·3 (3·5–5·2)	8194 (5·1%)	42 941 (26·2%)	54 771 (33·4%)	33 463 (20·3%)	24 804 (15·1%)
Numbers available	123 904 (60·3%)	132 424 (60·5%)	164 276 (75·0%)	164 173 (74·9%)	164 173 (74·9%)	164 173 (74·9%)	164 173 (74·9%)	164 173 (74·9%)	164 173 (74·9%)
Validation (n=199 431)	6230 (5·0%)	3·6 (2·9–4·3)	1·3 (1·1–1·6)	4·3 (3·5–5·2)	8378 (5·1%)	42 870 (26·2%)	54 499 (33·2%)	33 636 (20·4%)	24 771 (15·0%)
Numbers available	123 829 (59·0%)	132 575 (59·6%)	164 241 (73·8%)	164 154 (73·7%)	164 154 (73·7%)	164 154 (73·7%)	164 154 (73·7%)	164 154 (73·7%)	164 154 (73·7%)
p value	0·18	0·79	0·01	0·79	0·85	0·99	0·58	0·29	0·64

Data are n (%) for numbers available, median (IQR) for continuous variables, n (%) of participants or % (95% CI) for categorical variables. p values are given for the validation cohort versus the derivation cohort using weighted versions of the Mann-Whitney and χ^2^ test. Due to the presence of the Estonian case-cohort dataset, the summary estimates (% and median (IQR)) are weighted by the inverse of the inclusion probability for individuals in that cohort. The individuals from other cohorts are given weight 1 in the computation. Totals (n) are not weighted. From the Estonian data only, the sub-cohort is used in the table computations.

**Figure 1:**
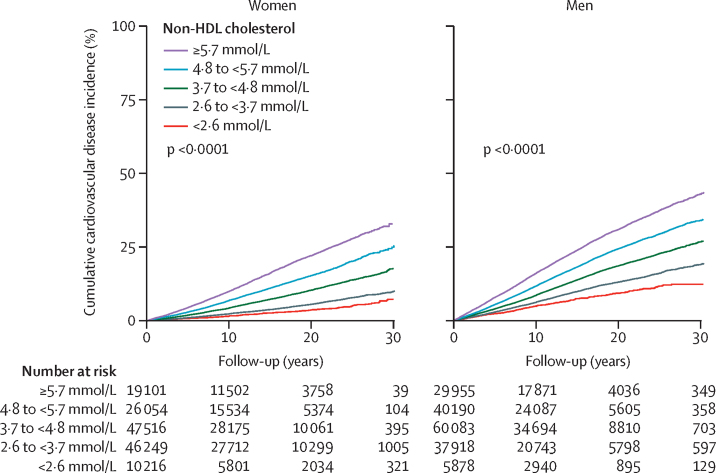
Incidence of cardiovascular disease across non-HDL cholesterol thresholds Cumulative incidence curves and numbers at risk for incident fatal and non-fatal cardiovascular disease according to non-HDL cholesterol concentration categories in women and men. Death from non-cardiovascular-disease causes was used as competing risk. p values are given for Gray's test comparing cumulative incidence curves.

**Figure 2:**
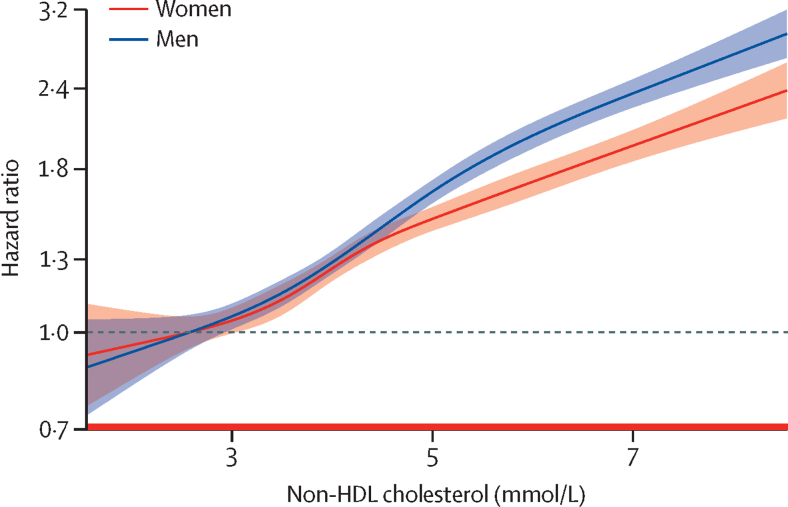
Sex-specific continuous association of non-HDL cholesterol and cardiovascular disease Sex-specific linear association of non-HDL cholesterol and cardiovascular disease risk (winsorised at 1·6 and 8·5 mmol/L). The Cox model used is adjusted for age, sex, study cohort, smoking, diabetes, body-mass index, systolic blood pressure, and antihypertensive medication. Non-HDL cholesterol was modelled using cubic splines. An interaction between sex and non-HDL cholesterol was included in the model. Median follow-up was 12·8 (IQR 7·5–18·4) years.

**Figure 3:**
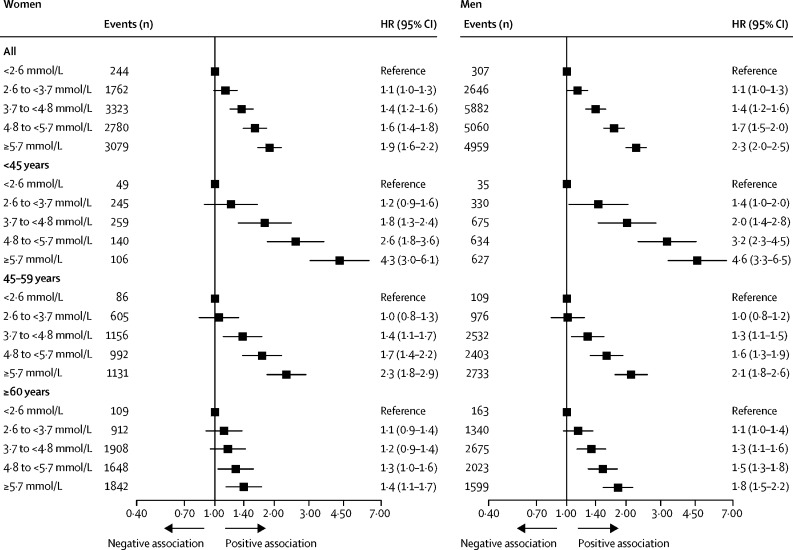
Age-specific and sex-specific association of non-HDL cholesterol and cardiovascular disease Lifetime sex-specific HRs for fatal and non-fatal cardiovascular disease (reference non-HDL cholesterol <2·6 mmol/L) in the overall cohort and according to three age categories (p<0·001 for the interaction of age and non-HDL cholesterol categories in women and in men). The Cox regression models were adjusted for age at baseline, sex, study cohort, smoking, diabetes, body-mass index, systolic blood pressure, and antihypertensive medication. HR=hazard ratio.

54 542 cardiovascular disease endpoints (37 888 in men, 16 654 in women; 27 185 in the derivation cohort, 27 357 in the validation cohort) occurred throughout the follow-up period. The predefined thresholds of non-HDL cholesterol concentrations strongly differentiated cardiovascular disease risk, particularly beyond 10 years, even in the lowest cholesterol categories (figure 1). The cumulative incidence curves showed a stepwise increase of cardiovascular disease events across increasing concentrations of non-HDL cholesterol. 30-year cardiovascular disease event rates were approximately three-to-four times higher in women and men in the highest non-HDL cholesterol category (≥5·7 mmol/L) than those in the lowest category (<2·6 mmol/L; 3253 [33·7%] *vs* 262 [7·7%] in women and 7689 [43·6%] *vs* 375 [12·8%] in men). Multivariable analyses confirmed a strong continuous association of concentrations of non-HDL cholesterol with the sex-specific cardiovascular disease risk. The lowest hazard for cardiovascular disease was found in women and men with the lowest non-HDL cholesterol concentrations, whereas there was a continuous and linear increase for higher non-HDL cholesterol concentrations (figure 2; the reference value [HR 1·0] was set as 2·6 mmol/L non-HDL cholesterol in both women and men). The hazard of cardiovascular disease increased progressively with higher non-HDL cholesterol categories compared with the reference category (figure 3). This association remained consistent in a sensitivity analysis using a random-effects multivariate meta-analysis (appendix p 31). The steepest increase of the relative hazard associated with non-HDL cholesterol was found in individuals younger than 45 years at baseline (maximum HR 4·3, 95% CI 3·0–6·1 in women and 4·6, 3·3–6·5 in men, for non-HDL cholesterol ≥5·7 mmol/L). Within the older groups, the association of non-HDL cholesterol with incident cardiovascular disease was attenuated but still detectable in individuals aged 60 years and older (HR 1·4, 95% CI 1·1–1·7 in women and 1·8, 1·5–2·2 in men, for non-HDL cholesterol ≥5·7 mmol/L; p<0·001 for the interaction of age and non-HDL cholesterol categories in women and in men; figure 3). Similar results were found in sensitivity analyses excluding individuals with diabetes (appendix p 38) and those receiving lipid-lowering therapy (appendix p 39) at baseline. The association of non-HDL cholesterol and cardiovascular disease remained consistent after additionally adjusting for HDL cholesterol (appendix p 40).

We found similar results for the association of blood concentrations of LDL cholesterol and incident cardiovascular disease to non-HDL cholesterol (appendix p 32). Cardiovascular disease event rates increased stepwise with increasing LDL cholesterol categories (appendix p 41). Multivariable analyses showed a linear association of LDL cholesterol with cardiovascular disease on the log-hazard scale, with the lowest relative risk for cardiovascular disease in the individuals with the lowest LDL cholesterol concentrations (appendix p 42). The strongest association was detected in individuals younger than 45 years and was attenuated in individuals aged 60 years and older (p<0·001 for the interaction of age and LDL cholesterol category in women and in men; appendix p 43).

**Figure 4:**
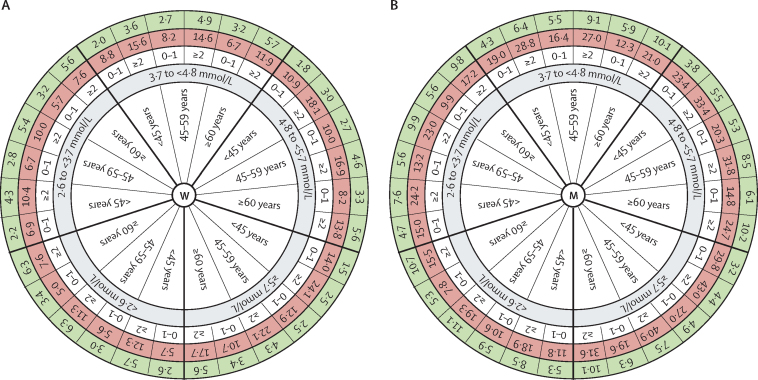
Model of long-term cardiovascular disease risk prediction and the benefit of lipid reduction Individual risk of fatal or non-fatal cardiovascular disease in women (A) and men (B) according to age, non-HDL cholesterol concentration, and the number of additional cardiovascular risk factors (daily smoking, arterial hypertension, diabetes, and obesity; white circle). The red circle represents the probability (%) of cardiovascular disease by the age of 75 years. The hypothetically achievable probability (%) for cardiovascular disease by the age of 75 years after 50% reduction of non-HDL cholesterol is given in the green circle. Corresponding 95% CIs are provided in the appendix (p 37).

To estimate the long-term probability of a cardiovascular disease event associated with non-HDL cholesterol, we established a model for cardiovascular disease risk up to the age of 75 years on the basis of a derivation–validation approach. Besides the non-HDL cholesterol category, sex, age, and the baseline number (≤1 *vs* ≥2) of classical modifiable cardiovascular risk factors (ie, arterial hypertension, diabetes, obesity, and smoking) were incorporated into this model (figure 4). For example, women with non-HDL cholesterol concentrations between 3·7 and 4·8 mmol/L, younger than 45 years, and with at least two additional cardiovascular risk factors had a 15·6% (95% CI 14·9–16·6) probability of experiencing a non-fatal or fatal cardiovascular disease event by the age of 75 years (28·8%, 28·1–29·5 in men with the same characteristics). The highest long-term risk of cardiovascular disease was seen in individuals younger than 45 years of age. The risk predictions obtained from this model were highly comparable in the derivation and validation datasets with a root mean square error lower than 1% for the estimated probabilities of cardiovascular disease (appendix p 44). Country-specific root mean square errors, as well as C-indices and smooth calibration curves specific for sex and follow-up time are provided in the appendix (pp 33, 34, 45). Further sensitivity analyses using time-dependent HRs did not show relevant differences in the prediction of cardiovascular disease by the age of 75 years (appendix p 46).

We calculated the optimally achievable risk reduction for cardiovascular disease by the age of 75 years assuming a 50% reduction of non-HDL cholesterol (figure 4). In the population with non-HDL cholesterol of 3·7–4·8 mmol/L, younger than 45 years, and with at least two risk factors, the long-term risk of cardiovascular disease could hypothetically be reduced from 15·6% (95% CI 14·9–16·6) to 3·6% (3·4–3·8) in women and from 28·8% (28·1–29·5) to 6·4% (6·3–6·6) in men. Further, corresponding numbers of patients needed to treat over the lifespan up to the age of 75 years, and achievable relative risk reduction (RRR) are shown in table 3. For example, in the same individuals, the number needed to treat to reduce one cardiovascular disease event over the lifespan by the age of 75 years was 8·3 in women and 4·5 in men, with RRR of 77% in women and 78% in men (see appendix p 35 for estimates with a 30% reduction of non-HDL cholesterol). Additionally, we computed the incidence for cardiovascular disease and the achievable risk reduction due to 50% reduction of non-HDL cholesterol for specific individuals starting at different ages. Absolute risk reductions of cardiovascular disease were more pronounced in individuals with two or more cardiovascular disease risk factors than in those with one or no risk factors, and in men than women. Within each category, the greatest effect was found for the youngest individuals (appendix pp 36, 47).

**Table 3:** Numbers needed to treat and RRR by age group and number of comorbid risk factors for non-HDL cholesterol concentration categories

		**<2·6 mmol/L**		**2·6 to <3·7 mmol/L**		**3·7 to <4·8 mmol/L**		**4·8 to <5·7 mmol/L**		**≥5·7 mmol/L**	
		NNT	RRR	NNT	RRR	NNT	RRR	NNT	RRR	NNT	RRR
**Women**											
<45 years											
	0–1	32·0	0·55	21·2	0·68	14·8	0·77	11·0	0·83	8·1	0·89
	≥2	15·3	0·53	11·0	0·68	8·3	0·77	6·7	0·83	4·6	0·90
45–59 years											
	0–1	38·8	0·46	25·3	0·59	18·1	0·67	13·7	0·73	9·7	0·80
	≥2	19·8	0·45	13·5	0·58	10·3	0·67	8·1	0·73	5·6	0·81
≥60 years											
	0–1	63·4	0·32	40·2	0·43	28·9	0·52	20·8	0·59	13·8	0·68
	≥2	36·0	0·31	23·8	0·43	16·3	0·52	12·3	0·59	8·3	0·68
**Men**											
<45 years											
	0–1	15·5	0·55	9·7	0·69	6·8	0·78	5·1	0·84	3·8	0·89
	≥2	9·6	0·55	6·0	0·69	4·5	0·78	3·6	0·84	2·6	0·90
45–59 years											
	0–1	21·4	0·44	13·1	0·58	9·1	0·67	6·7	0·74	4·5	0·82
	≥2	12·2	0·42	7·6	0·57	5·6	0·66	4·3	0·73	3·0	0·82
≥60 years											
	0–1	39·8	0·32	23·3	0·43	15·7	0·52	11·4	0·59	7·6	0·68
	≥2	20·6	0·31	13·5	0·43	9·2	0·52	6·9	0·59	4·7	0·68

Sex-specific and age-specific estimated NNT to avoid one cardiovascular disease event and RRR for cardiovascular disease by age 75 years. The model used is assuming a hypothetical 50% reduction of non-HDL cholesterol. NNT=numbers needed to treat. RRR=relative risk reduction.

## Discussion

Using individual-level data from the Multinational Cardiovascular Risk Consortium of individuals without prevalent cardiovascular disease, we characterised the age-specific and sex-specific long-term association of non-HDL cholesterol with cardiovascular disease. On the basis of this association, we derived and validated a tool specific for age, sex, and cardiovascular risk factors to assess the individual long-term probability of cardiovascular disease by the age of 75 years associated with non-HDL cholesterol. Further, we modelled the potentially achievable long-term cardiovascular disease risk, assuming a 50% reduction of non-HDL cholesterol.

The proatherogenic effects of apoB-containing lipoproteins are largely based on the development and progression of atherosclerotic plaques and by accumulation of these lipoproteins within the arterial intima.^20^, ^21^ Although the measurement of LDL cholesterol can be influenced by increased triglycerides^22^, ^23^ in cardiovascular risk assessment, an appropriate way to estimate the amount of apoB-containing lipoproteins is the determination of non-HDL cholesterol.^3^, ^21^, ^24^, ^25^ Therefore, our main analyses are based on non-HDL cholesterol. In line with previous studies, we found a comparable prognostic relevance for non-HDL cholesterol and LDL cholesterol.^26^ A large body of evidence including inherited disorders,^27^ genetic^28^, ^29^ and epidemiological studies,^10^, ^11^ and clinical trials^7^, ^8^, ^9^ leads to the hypothesis that reducing apoB-containing lipoproteins, regardless of the method, should yield a corresponding reduction in cardiovascular events. To intervene early and intensively during the lifespan might even lead to regression of early manifestation of atherosclerosis.^30^ Epidemiological studies have shown the association between cholesterol concentration and cardiovascular mortality during long-term follow-up.^11^, ^31^ Furthermore, repeated non-HDL cholesterol testing in 2516 individuals of the Framingham Offspring Study suggested that stable concentrations over the lifetime^13^ might represent increased risk of cardiovascular disease. Clinical trials, however, mostly address older (age >60 years for most studies) individuals with prevalent cardiovascular disease or at markedly increased cardiovascular risk and report observations over short follow-up intervals, varying between 2 and 7 years.^7^, ^8^, ^9^

Considerable uncertainty exists about the extent to which slightly increased or apparently normal cholesterol concentrations affect lifetime cardiovascular risk and about which thresholds should be used to merit a treatment recommendation, particularly in young people. Our study extends current knowledge because it suggests that increasing concentrations of non-HDL cholesterol predict long-term cardiovascular risk, particularly in cases of modest increase at a young age. Only a few studies have reported the age-dependent association of lipids and cardiovascular disease with focus on young individuals.^10^, ^11^, ^13^ In our analyses, individuals younger than 45 years showed the strongest HRs for the association of blood lipids with the incidence of cardiovascular disease during long-term follow-up. This effect is probably related to the following considerations: (1) the effect of lifetime accumulation of proatherogenic lipids in the vulnerable period of the fourth and fifth decades of life,^32^ (2) the shorter timespan that individuals aged 60 years and older have to achieve the cardiovascular disease endpoint due to their age itself, and (3) the fact that people with prevalent cardiovascular disease are not included in our study and thus individuals who reach the age of 60 without prevalent cardiovascular disease are a population enriched with protective characteristics against the proatherogenic effect of cholesterol. However, although there is a weaker association of non-HDL cholesterol with cardiovascular disease in older individuals (>60 years), these people are at higher absolute risk than young individuals, and so the more modest relative risks represent large absolute risk differences across non-HDL cholesterol categories.^10^

The risk circle tool displays the individual lifetime risk of non-fatal and fatal cardiovascular disease by the age of 75 years that is associated with non-HDL cholesterol and is specific for the burden of age, sex, and risk factor. It also estimates the corresponding risk reduction from lipid-lowering therapies. The risk scores currently used for decision making about lipid-lowering intervention assess only the 10-year cardiovascular risk^33^, ^34^ and therefore underestimate lifetime risk, particularly in young individuals.^11^, ^12^ Pencina and colleagues^31^ estimated the 30-year risk of cardiovascular disease in the Framingham Offspring cohort and showed 30-year risks that were up to ten times higher than the projected 10-year risks in young people.^31^ Therefore, in young individuals, the long-term risk of cardiovascular disease should constitute the basis of primary prevention. Since the risk factor burden is incorporated as a categorical variable in our model, we cannot provide a weighting among the different risk factors or a quantitative consideration of every single risk factor as do other risk models. Instead, because of its simplicity, our risk circle tool provides an opportunity to estimate lifetime risks based on non-HDL cholesterol in an accessible and easily understood way that can improve physician–patient communication about preventive strategies in clinical practice. The modelled risk reduction is calculated hypothetically and is based on a 50% lipid reduction leading to a decrease of the cumulative burden of non-HDL cholesterol. Since the calculated numbers needed to treat are based on different timeframes for different age ranges, they have to be compared carefully between the different age categories. They have to be interpreted individually for a certain spectrum of ages, sexes, and risk factors to guide decision making on any lipid-lowering intervention. Furthermore, numbers needed to treat might be higher than those we showed in low-risk countries with lower than average cardiovascular disease incidence and mortality.

The following limitations merit consideration. First, due to the population-based longitudinal study design, the endpoint information is mainly based on medical reports or local registers and we cannot exclude misclassification of endpoints for some of the observed effects. However, endpoint information was harmonised on the basis of individual data according to the specification of the MORGAM study, and multivariable analyses were adjusted for potential confounders. Therefore, the best achievable data quality can be assumed for the current analyses. Second, only baseline data of blood lipids were available for current analyses. No information about dynamic changes during follow-up or about the initiation of lipid-lowering therapy, particularly in those individuals with very high blood concentrations, could be considered.^35^ However, non-HDL cholesterol concentrations in young individuals are generally stable over the 30-year life course,^13^ and not adjusting for changes in risk factors was shown to have little bearing on lifetime cardiovascular disease risk.^31^ Third, our cohorts largely included individuals with European ancestry from high-income countries in Europe, North America, and Australia. The generalisability of our findings to other regions or individuals from other racial and ethnic groups is unknown. Finally, the therapeutic benefit of lipid-lowering intervention investigated in our study is based on a hypothetical model that assumes a stable reduction of non-HDL cholesterol. We recognise that making such an assumption posits that treatment effects are sustained over a much longer term than has been studied in clinical trials and that real-world benefits of taking statins are probably lower than those shown in trials because of adherence and side-effects.^36^ However, since clinical trials investigating the benefit of lipid-lowering therapy in individuals younger than 45 years during a follow-up of 30 years are not available, our study provides unique insights into the benefits of a potential early intervention in primary prevention. We agree with Hippisley-Cox and colleagues^37^ that future research needs to address many related questions, including whether intervention in young people with a high lifetime risk but low 10-year risk would yield greater benefits than late intervention.

**This online publication has been corrected. The corrected version first appeared at thelancet.com on December 6, 2019**
