# Department of Error

**DOI:** 10.1016/S0140-6736(19)33059-4

**Published:** 2019-12-14

*Brunner FJ, Waldeyer C, Ojeda F, et al. Application of non-HDL cholesterol for population-based cardiovascular risk stratification: results from the Multinational Cardiovascular Risk Consortium.* Lancet *2019; **394:** 2173–83*—The licence type for this Article has been changed to Gold Open Access. This correction has been made to the online version as of Dec 6, 2019, and the printed version is correct.

