# Supplementary material for: Application of non-HDL cholesterol for population-based cardiovascular risk stratification: results from the Multinational Cardiovascular Risk Consortium
Source: Lancet. 2019 Dec 14;394(10215):2173–83. doi: 10.1016/S0140-6736(19)32519-X (PMC6913519; doi:10.1016/S0140-6736(19)32519-X)
Supplement: Supplementary appendix [file mmc1.pdf]

THE LANCET

Supplementary appendix

This appendix formed part of the original submission and has been peer reviewed. We post it as supplied by the authors.

Supplement to: Brunner FJ, Waldeyer C, Ojeda F, et al. Application of non-HDL cholesterol for population-based cardiovascular risk stratification: results from the Multinational Cardiovascular Risk Consortium. *Lancet* 2019; published online Dec 3. http://dx.doi.org/10.1016/S0140-6736(19)32519-X.

Supplementary appendix

Application of Non-HDL-Cholesterol for Population-based Cardiovascular Risk Stratification Results from the Multinational Cardiovascular Risk Consortium

Table of contents

The Multinational Cardiovascular Risk Consortium ... 2
Overview about the MORGAM/BiomarCaRE cohorts ... 2
Overview about the non-MORGAM/non-BiomarCaRE cohorts ... 7
Affiliations of the consortium members ... 12
Online-only methods ... 15
Detailed definition of cardiovascular risk factors ... 15
Detailed definition of endpoints ... 16
Online-only tables ... 19
Table S1: Baseline information – MORGAM/BiomarCaRE cohorts ... 19
Table S2: Baseline information – non-MORGAM/non-BiomarCaRE cohorts ... 25
Table S3: Individuals on lipid lowering therapy ... 28
Table S4: Sex-specific time dependent hazard ratios for non-HDL-cholesterol categories. ... 29
Table S5: Sex-specific baseline characteristics for derivation and validation cohorts ... 30
Table S6: Association of non-HDL-cholesterol categories with CVD – random effects ... 31
Table S7: LDL- vs. non-HDL-cholesterol – C-indices for the prediction of CVD ... 32
Table S8: Cross validation for each country ... 33
Table S9: Risk tool – C-indices for the prediction of CVD ... 34
Table S10: 30% reduction of baseline non-HDL-cholesterol ... 35
Table S11: Additive CVD risk and exemplary risk reduction ... 36
Table S12: Long-term CVD risk prediction and the benefit of lipid reduction ... 37
Online-only figures ... 38
Figure S1: Association of non-HDL-cholesterol and CVD in individuals without diabetes mellitus ... 38
Figure S2: Association of non-HDL-cholesterol and CVD in individuals without lipid lowering therapy ... 39
Figure S3: Association of non-HDL-cholesterol and CVD additionally adjusted for HDL-cholesterol ... 40
Figure S4: Incidence of CVD across LDL-cholesterol thresholds ... 41
Figure S5: Sex-specific continuous association of LDL-cholesterol and CVD ... 42
Figure S6: Age- and sex-specific association of LDL-cholesterol and CVD ... 43
Figure S7: Diagnostic accuracy in derivation and validation datasets ... 44
Figure S8. Smooth calibration curves. ... 45
Figure S9: Predicted CVD risk – time dependent hazard ratios ... 46
Figure S10: Additive CVD risk and exemplary risk reduction ... 47
Funding source of participating cohorts ... 48
Online-only references ... 51

The Multinational Cardiovascular Risk Consortium

Overview about the MORGAM/BiomarCaRE cohorts

Study/cohort	Country	Study/cohort full name and short description
ATBC[1]	Finland	The Alpha-Tocopherol, Beta-Carotene Cancer Prevention (ATBC) Study is a cohort of male smokers (aged 50-69 years) recruited in 1985-1988. The participants were screened from the total male population aged 50-69 years living in southern and western Finland (N=290,406) through a postal questionnaire on their smoking habits and willingness to participate. Smokers of at least 5 cigarettes per day and willing to participate were invited to the local study centre for further evaluation of their eligibility. Participants were excluded due to a history of cancer or serious disease limiting their ability to participate or use of excess vitamin supplements. In all, 29,133 men were randomized to receive in a controlled trial design either alpha-tocopherol, or beta-carotene, or both, or placebo, and 99·9% of them donated a baseline serum sample. Men were prospectively followed up for cardiovascular and cancer endpoints until 2004 using record linkage to national Hospital Discharge Register, Cancer Registry, and Register of Causes of Death with validation described elsewhere.[1–3] http://www.thl.fi/publications/morgam/cohorts/full/finland/fin-atba.htm http://atbcstudy.cancer.gov/
DanMONICA Study, RCPH[4]	Denmark	The DanMONICA cohorts from the Research Center for Prevention and Health (RCPH) are three prospective population-based cohorts from 11 municipalities from the western part of the suburbs of Copenhagen, Denmark. Random sampling was based on the national population register, stratified by sex and year of birth. Cohort 1 and 3 consists of men and women aged 30-70 years and cohort 2 consists of men and women aged 30-60. Cohort 1 was collected in 1982-1984 (N=4052). Cohort 2 (N=1504) was examined in 1986-1987 and cohort 3 (N=2026) was examined in 1991-1992. Follow-up is achieved through linkage to the National Cause of Death Register and National Hospital Discharge Register, with endpoint diagnosis based on MORGAM criteria and validation described elsewhere.[5] The follow-up for the cohorts 1, 2, and 3 were completed to 31st December 2010. http://www.thl.fi/publications/morgam/cohorts/full/denmark/den-gloa.htm
EGCUT[6]	Estonia	Estonian Genome Center of the University of Tartu (EGCUT) – The Estonian Biobank: The Estonian Biobank is a population-based biobank of the Estonian Genome Center of the University of Tartu (EGCUT). The project is conducted in accordance with the Human Genes Research Act of Estonia (www.biobank.ee) and all subjects have been recruited randomly, on a voluntary basis by general practitioners and physicians in hospitals. As of June 2013, the number of individuals is 51,713, which represents about 5% of Estonia's adult population. Phenotyping of subjects (18-103 years of age) was performed by Computer Assisted Personal interview (CAPI), including personal and genealogical data, educational and occupational history, and lifestyle factors. Follow-up of incident fatal and non-fatal coronary heart disease and stroke events of a subset of the cohort is on-going as our database is being linked with the national healthcare registries and regional and central hospital databases. Events are recorded according to the International Classification of Diseases (ICD-10). All subjects provided written informed consent prior to participation and the approval for the study was granted by the Ethics Review Committee on Human Research at the University of Tartu. For current analyses a case-cohort set of 4,971 persons was available. http://www.biobank.ee
ESTHER Study[7]	Germany	The ESTHER study (Epidemiologische Studie zu Chancen der Verhütung, Früherkennung und optimierten Therapie chronischer Erkrankungen in der älteren Bevölkerung [German]) is a prospective population-based cohort study in the federal state of Saarland, Germany. In total 9,949 men and women (aged 50-74 years at baseline) were recruited during a routine health check-up between 2000 and 2002. Follow-up was completed by 31st of December 2010. Approval for the study was granted by the Ethics Committees of the Medical Faculty Heidelberg at the University of Heidelberg and the Physicans' Board of Saarland. All participants provided written informed consent. https://thl.fi/publications/morgam/cohorts/full/germany/ger-esra.htm
FINRISK[8]	Finland	The FINRISK study is a series of population-based cardiovascular risk factor surveys carried out every five years in five (or six in 2002) districts of Finland, including North Karelia, Northern Savo (former Kuopio), Southwestern Finland, Oulu Province, Lapland province (in 2002 only), and the region of Helsinki and Vantaa. A stratified random sample was drawn for each survey from the national population register; the age-range was 25-74 years. All individuals enrolled in the study received a physical examination, a self-administered questionnaire, and a blood sample was drawn. FINRISK cohorts are based on surveys carried out in 1982, 1987, 1992, 1997 and 2002 were used in this analysis. The numbers of participants in each survey are shown in **Table S2** and the participation rates can be found in the web address below. During follow-up, the National Hospital Discharge Register, the National Causes of Death Register and the National Drug Reimbursement Register were used to identify endpoints. In these analyses, the follow-up extends until 31st December 2010. The Coordinating Ethics Committee of the Helsinki and Uusimaa Hospital District approved the study, which followed the declaration of Helsinki. All subjects gave their informed consent.

		http://www.thl.fi/publications/morgam/cohorts/full/finland/fin-fina.htm
GHS[9]	Germany	The Gutenberg Health Study (GHS) is designed as a community-based, prospective, observational, single-center cohort study in the Rhine-Main area of Western Germany. The sample was drawn randomly from the governmental local registry offices in the city of Mainz and the district of Mainz-Bingen. The sample was stratified 1:1 for sex and residence (urban and rural) and in equal strata for decades of age. Individuals between 35 and 74 years of age were enrolled. Exclusion criteria were insufficient knowledge of the German language and physical or psychological inability to participate in the examinations at the study center. Baseline examination of 15,000 study participants was performed between 2007 and 2012. A 2·5 year follow-up conducted as a telephone interview started in 2009. Since 2012, the 5-year follow-up has been achieved through record linkage, including a second visit at the study centre with extensive medical examination and re-sampling of the biomaterial which is ongoing. For current analyses, the endpoint available in GHS was overall mortality. http://www.gutenberghealthstudy.org/
Kaunas Study[10]	Lithuania	Epidemiological studies of heart disease in Kaunas, event registration and population surveys, were started in 1972 as part of the myocardial infarction register and the Kaunas-Rotterdam Intervention study (KRIS) coordinated by WHO. The latter study included only men. In Kaunas, the KRIS study still continues as a cohort study. The Kaunas-MONICA study was the first to include both sexes in risk factor surveys, and it also initiated stroke registration. The main results of Kaunas-MONICA have been used to prepare strategies for health promotion and prevention of non-communicable diseases at local and national levels. Since 1994 mortality rates from the main cardiovascular diseases have declined in Lithuania.[11] For current analyses, the MONICA cohorts from 1983-85, 1986-1987, and 1992-1993 were used. Follow-up is completed to 31st December 2013. Non-fatal strokes, and hence CVD3, were followed up only up to age 65. https://thl.fi/publications/morgam/cohorts/full/lithuania/ltu-kaua.htm
KORA, MONICA[12]	Germany	The WHO Multinational Monitoring of Trends and Determinants in Cardiovascular Diseases (MONICA)/ Cooperative Health Research in the Region of Augsburg (KORA) cohorts comprise all respondents from representative sample surveys from the city of Augsburg and the less urban Landkreis Augsburg and Landkreis Aichach-Friedberg regions in Bavaria, Southern Germany. A list of municipalities and population registers was used as sampling frame for the first and the second stage of two-stage sampling, respectively. The second stage of sampling was stratified by sex and ten-year age groups. The Surveys 1 (S1) (baseline: 1984/85; n=4022), S2 (baseline: 1989/90; n=4940) and S3 (baseline: 1994/1995; n=4856) were carried out as part of the WHO MONICA project and S4 (baseline: 1999-2001; n=4261) was carried out within KORA. Participants were aged 25-64 (S1) and 25-74 years (S2-S4) at baseline. Response rates ranged from 79% (S1) to 66% (S4). Follow-up questionnaires were sent to the participants in 2002 and 2009. Coronary events within the study area occurring at ages below 75 years were identified through the MONICA/KORA Augsburg coronary event registry.[13] Non-fatal coronary events which occurred outside the study area and in participants aged ≥75 years and all incident strokes were identified by questionnaires. Mortality follow-up until 2009 was conducted through national death registers and fatal coronary events and fatal strokes were identified through death certificates. Coronary events and strokes were validated by autopsy reports, death certificates or medical records. Information on incident stroke is only available for S3 and S4 in the MORGAM database, therefore, the composite variable on incident CVD is also only available for S3 and S4. http://www.thl.fi/publications/morgam/cohorts/full/germany/ger-auga.htm
Krakow Study[14]	Poland	The Krakow study is based on residents aged 25–64 of the south-eastern rural province of Tarnobrzeg Voivodship, Poland. Tarnobrzeg Voivodship was chosen for POLMONICA Krakow to contrast its rural population and health care with that of Warsaw. The Project also promoted cardiovascular disease prevention. However, the information on incident CVD is absent in the Krakow study. Additional survey data was used locally and for the Poland and US Collaborative Study on Cardiopulmonary Epidemiology. MONICA monitored risk factors, medical care and trends in coronary heart disease mortality (which increased up to, and decreased after, 1992) during major political, economic and social changes.[11] Three cohorts, from years 1983-84, 1987-88 and 1992-93 were used for the current analysis. Follow-up, completed to 31st December 1998, covers fatal cases only. https://thl.fi/publications/morgam/cohorts/full/poland/pol-tara.htm
MATISS Rome Study[15]	Italy	Participants of the MATISS (Malattie cardiovascolari ATerosclerotiche, Istituto Superiore di Sanità [Italian]) study were recruited in the district of Latina between 1983 and 1987 (N=8,265) and 1993-1996 (N=1,970). Electoral rolls were used as sampling frames in the single-stage sampling, which was stratified by the municipality, sex and 5-year age group. The baseline examination was partly carried out as part of the WHO MONICA Project, where it was identified with code ITA-LAT. The other examinations were carried out as part of the MATISS Project using the WHO MONICA methods. Individuals were 20 to 69 years old. From the first screening (1983) to the last screening (1996), municipalities were contacted every five years for information about vital status, emigration and residency; from 1996 onwards, municipalities were contacted every year. The follow-up procedure covers the Lazio Region. If a person moved out of the Lazio Region, then he/she was lost to follow-up since the date of emigration. Follow-up was completed to 31st December 2004.

		https://www.thl.fi/publications/morgam/cohorts/full/italy/ita-roma.htm
Moli-Sani Study[16]	Italy	The cohort of the Moli-Sani study was recruited in the Molise region from city hall registries by multistage sampling. First, townships were sampled in major areas by cluster sampling; then, within each township, participants aged 35 years or older were selected by simple random sampling. Exclusion criteria were pregnancy at the time of recruitment, inability to comprehend, current multiple trauma or coma, or refusal to sign the informed consent. A total of 24,325 men (47%) and women (53%) were examined at baseline from 2005 to 2010. Participation was 70%. The cohort was followed up for a median of 4·2 years (maximum 6·5 years) at December 2011 (follow-up data was used up to December 2011). Follow-up is achieved through record linkage to national mortality registries and hospital discharge registers, validation of events was achieved through hospital record linkage and doctor's medical records using updated MORGAM criteria.<br>http://www.moli-sani.org/
MONICA Brianza Study[17]	Italy	The MONICA-Brianza Cohort Study is a prospective observational study of three cohorts of 25-64 years old residents in Brianza, a highly-industrialized area located between Milan and the Swiss border, Northern Italy. Gender- and ten-year age-stratified samples were randomly drawn in 1986, 1990, and 1993, and cardiovascular risk factors were investigated at baseline following the procedures of the WHO MONICA Project. The overall participation rate was 69%. For all subjects whole-blood and serum samples were stored in a biobank. The protocol was approved by the Monza Hospital Ethical Committee. Study participants were followed up for first coronary or stroke events, fatal and non-fatal, up to the end of 2008, for a median of 15 years.<br>http://epimed.uninsubria.eu
MONICA Catalonia[18,19]	Spain	The Catalonia Study consists of two cohorts sampled from representative surveys from the central area of Catalonia and part of the metropolitan area of Barcelona, Spain. The first stage drew a random sample of individuals from nine municipalities with probability proportional to population size. In the second stage, a random sample of men and women from the municipal population registries were used. As a sampling frame, a random sample from each municipal population was stratified by sex and ten-year age groups, 25-64 years, of a fixed number, was selected. Baseline examinations were carried out in 1986-1988 for cohort 1 (Round 1) of N=2571, response rate was 74%. Cohort 2 (Round 1) was collected in 1990-1992 (N=2936), response rate was 67%. Non-fatal strokes were mostly self-reported, and therefore not included in the composite endpoint of CVD for current analyses. Participants were mainly from the Industrial and services economy. All subjects gave informed consent. The project was approved by the Institute of Health Studies steering committee. Follow-up until 1997 for Cohort 1 and until 1999 for Cohort 2 was achieved through follow-up questionnaires and record linkage with MONICA registers, national mortality index register and hospital discharge registers.
MONICA Friuli[20]	Italy	The Friuli population cohorts were collected as part of the WHO MONICA surveys. The Friuli area covers three provinces of north-east Italy (Friuli-Venezia-Giulia) recruited using a single stage sampling frame from the official Regional Health Roll stratified by Health Unit (combination of municipalities covering 40,000 inhabitants), sex and 5-year age group. Four cohorts were included in these analyses. Follow-up through registry linkage is available until 1998 collected through the framework of the Progetto Cuore.<br>http://www.cuore.iss.it/eng/assessment/procedures.asp
MONICA Newcastle[21]	Australia	The MONICA Newcastle cohort consists of residents aged 25–69 years of the five local government areas of Newcastle, Lake Macquarie, Port Stephens, Maitland, and Cessnock participated in the MONICA Project in Newcastle. There were 76,831 men and 76,502 women aged 35 to 64 years in the study population in 1991. Three surveys of risk factors were conducted during the study period, the first in 1983, the second in 1988 and 1989 and the third in 1994. Information about incident CVD was not available.<br>https://thl.fi/publications/morgam/cohorts/full/australia/aus-newa.htm
MONICA PAMELA	Italy	The PAMELA study collected a representative sample of N=2,044 men and women aged 25-74 in the region of Brianza, Italy. The city population register was used as a sampling frame for the single state sampling. This sample was stratified by sex and age in 10-year age groups and a random sample of 300 subjects was added to each stratum. The baseline survey was carried out between 1990 and 1993 and after an extension of the follow-up time, it was finalized on the 31st December 2002. The study investigated the following end-points: non-fatal acute myocardial infarction, cardiac revascularization, non-fatal stroke events, and death.
Northern Sweden MONICA Study[22]	Sweden	The Northern Sweden cohort was recruited in the Västerbotten and the Norrbotten counties. Both counties are a sparsely populated area, half rural and half urban with higher-than-average mortality and unemployment and low socio-economic status. The cohort was formed by the respondents of representative sample surveys with an age from 24 to 75. National population register was used as sampling frame for the single stage sampling which was stratified by sex and 10-year age group. The baseline examinations were partly carried out as part of the WHO MONICA Project. Recruitment was performed in 1986 (N=1,625), 1990 (N=1,576), 1994 (N=1,893), 1999 (N=1,789), 2004 (N=1,863) and 2009 (N=1,704). The response rate was between 68 and 81%. Everyone in Sweden has a unique personal identification code issued by the National Tax Authority. The cohorts were linked to the registers mentioned above using the personal identification code, all dates of death, and the

		causes of death were obtained until the 31st December 2011. http://www.org.umu.se/monica https://www.thl.fi/publications/morgam/cohorts/full/sweden/swe-nswa.htm
Novosibirsk Study[23]	Russia	For the Novosibirsk Study residents aged 25–64 of the city of Novosibirsk, central West Siberia, the industrial and scientific centre of Siberia, were included. Coronary heart disease and stroke morbidity and mortality rates are high in men and women in Novosibirsk. The total population in 1991 was 482,000 (Novosibirsk Control, NOCb) and 160,000 (Novosibirsk Intervention). Population surveys followed the MONICA protocol but with some additional items added. In this analysis we used four cohorts: cohort 1 (Round 1) with N=3,065, cohort 2 and 3 (Round 1) with 6,357, and cohort 4 (Round 1) with 1,546 individuals. The high cardiovascular mortality in Novosibirsk increased dramatically at the beginning of the 1990s but declined modestly after 1994.[11] The follow-up is completed to 31st December 1998, with upper age limit of 64 years. https://thl.fi/publications/morgam/cohorts/full/russia/rus-nova.htm
PRIME[24]	United Kingdom and France	The PRIME (Prospective Epidemiological Study of Myocardial Infarction) study examined the classic and putative cardiovascular risk factors to explain the large difference in heart disease incidence between Ireland and France. The study includes four cohorts of men aged 50-59; from Belfast, Northern Ireland (N=2,745) and Lille (N=2,633), Toulouse (N=2,610) and Strasbourg (N=2,612) in France. Baseline examinations took place in 1990-1993 and targeted cohorts which had broadly similar social class structures to the background population, initially sampling from industries and various employment groups, employment groups with more than 10% of their workforce of foreign origin were excluded. Follow-up for 10 years for each participant (Toulouse, Strasbourg and Lille) and for 18 years (Belfast) was achieved through annual follow up questionnaires with verification against national death registers, medical records, hospital discharge diagnoses. Endpoints were validated by expert medical committee. http://www.thl.fi/publications/morgam/cohorts/full/uk/unk-bela.htm
SHHEC[25]	Scotland	The Scottish Heart Health Extended Cohort (SHHEC) comprises different cohorts of men and women aged 25-64 (25-75 in SHHEC 2) recruited randomly across Scottish districts from 1984-1995 in the Scottish Heart Health Study and Scottish MONICA in contribution to the WHO MONICA Project. Apart from age differences the surveys following the same protocol. Of 18,107 in SHHEC, those 15,999 with the most complete risk factor data were entered into the MORGAM study and thence the MORGAM Biomarker Study and BiomarCaRE. Follow-up data extends to the end of 2009 using the Scottish National Health Service Central Register and the Scottish Record Linkage System for mortality and cardiovascular endpoints. https://www.thl.fi/publications/morgam/cohorts/full/uk/unk-sco.htm
SHIP[26]	Germany	SHIP (Study of Health in Pomerania) is a population-based project conducted in Northeast Germany. The study aims to assess prevalence and incidence of common risk factors, subclinical disorders and clinical diseases and to investigate associations and interactions among them using comprehensive medical assessments. The first SHIP cohort was recruited between 1997 and 2001 and included 4,308 individuals at baseline (SHIP-0, 20-70 years, response 68·8%), 3,300 after five years (SHIP-1) and 2,333 after 11 years (SHIP-2). In parallel to SHIP-2, baseline examinations of a second, independent cohort (SHIP-TREND) were conducted in 4,420 participants (20-79 years, response 50·3%). For the current analysis only data of SHIP-0 was used. Data were transferred from Greifswald. The harmonization was implemented in Hamburg. SHIP is one of the MORGAM/BiomarCaRE cohort studies and one of the population-based projects with very comprehensive examinations including interviews, cardio-metabolic ultrasound exams, cardiopulmonary exercise tests and whole-body magnetic resonance imaging in a general population setting. In addition to the examination follow-ups, information on fatal and non-fatal disease is collected on a regular basis. However, information of incident CVD is lacking. Mortality follow-ups are conducted semi-annually by record linkage with data bases of the regional population registry. Causes of death are defined from the official death documents provided by regional health authorities. Active follow-ups for non-fatal diseases are performed biannually and by interviews during follow-up examinations every five years. http://www.medizin.uni-greifswald.de/cm/fv/ship.html
Tromsø Study[27]	Norway	The Tromsø Study is a prospective repeated population-based health survey of men and women aged 20-97 years in Northern Norway. Specific age groups in the municipality were invited to the different surveys and over time this enabled collection of repeated risk factor measurements in many subjects. The 3rd Tromsø Study survey (Tromsø) was carried out in 1986-87 and those invited were all men in the 20-61 age group, all women in the 20-56 age group, a randomly selected 10% sample from the 12-19 age group (born 1967-1974) and a subsample who were included in a family intervention study. A total of 21,826 participated, 75% of the invited. Of these, data on all men and women aged 20-59 years are included in the BiomarCaRE study (N=20,300). A total of 27,158 men and women participated in the subsequent Tromsø 4 survey (participation rate 77%). This analysis comprises two cohorts with N=4,851 (1986-1987 and N=25,705 (1994-1995). The cohorts are being followed up with registration of incident myocardial infarction, stroke (ischemic, hemorrhagic, subarachnoid haemorrhage and unclassifiable), diabetes, atrial fibrillation and cause-specific death. Cases of incident events were identified by linkage to the diagnosis registry at the University Hospital of North Norway (the only

		hospital in the region) and to the National Causes of Death Registry. Validation of hospitalized and out-of hospital events was performed by an independent endpoint committee and based on data from hospital and out-of hospital journals, autopsy records, and death certificates. Slightly modified World Health Organization MONICA/MORGAM criteria for myocardial infarction[28] and stroke[29] were used. For the current analyses Tromsø 4 individuals were used together with those Tromsø 3 individuals not participating in the Tromsø 4 survey. http://tromsoundersokelsen.uit.no/tromso/
Warsaw Study[30]	Poland	The Warsaw study includes residents aged 25–64 of the two districts of the capital city of Warsaw east of the Vistula. These districts are partly industrial and partly residential and home to hospitals, banks, governmental offices and universities. Poland's changing economy has affected the living conditions and behaviour of the population. In 1989/90 the free market produced mixed benefits: loss of State social support, high inflation, high unemployment, but greater access to food products previously found only in the western markets. Risk-factor profiles have changed. Cardiovascular disease mortality, previously rising, began to decrease from 1991. The total population in 1991 was 494,000.[11] In this analysis used two cohorts with N=2,239 and N=2,550, respectively. Fatal events were followed up till 31st December 1998 and non-fatal events till 31st December 1994 with upper age limit of 64 years. https://thl.fi/publications/morgam/cohorts/full/poland/pol-wara.htm

Overview about the non-MORGAM/non-BiomarCaRE cohorts

Study/cohort	Country	Study/cohort full name and short description
AusDiab[31]	Australia	The baseline study conducted in 1999–2000 provided benchmark national data on the prevalence (or number of people) of diabetes, obesity, hypertension, and kidney disease in Australia. The second phase of AusDiab, completed in December 2005, was a five-year follow-up of the people who participated in the baseline survey. A twelve-year follow-up was completed in 2012. AusDiab is a national population-based longitudinal study established to examine the prevalence of diabetes and related risk factors in Australia. Using a stratified cluster sampling method, it enrolled 11,247 adults (aged 25 years and above) from 42 randomly selected census collector districts across Australia between May 1999 and December 2000. Over 85% of the participants were from an Australian, New Zealand or British background. The baseline survey consisted of an initial household interview and was followed by biomedical exams. At baseline and follow-ups, anthropometric measures, and fasting blood samples were collected and participants completed interviewer-administered questionnaires on health and lifestyle factors. Participants underwent a standard 75 g oral glucose tolerance test and T2D was classified as fasting plasma glucose ≥7·0 mmol/L, 2 h plasma glucose ≥ 11·0 mmol/L or current treatment with insulin or oral hypoglycemic agents.<br>https://www.baker.edu.au/ausdiab/
Atherosclerosis Risk in Communities Study (ARIC)[32]	USA	ARIC (NCT00005131) is a large-scale, long-term prospective study that measures associations of established and suspected coronary heart disease risk factors with both atherosclerosis and new CHD events in men and women from four geographically diverse communities. The project has two components: community surveillance of morbidity and mortality; and repeated examinations of a representative cohort of men and women in each community. The community surveillance involves abstracting hospital records and death certificates and investigating out-of-hospital deaths. The representative cohorts include approximately 4,000 persons from each community. Community surveillance data includes detailed hospital record abstraction, ECG tracings, and event adjudication. Data from out-of-hospital events in the community include physician, informant, and coroner questionnaires as well as death certificate data and event adjudication.<br>All cohort participants were examined four times at three year intervals and contacted annually to update their medical histories. Atherosclerosis was measured by carotid ultrasonography. Risk factors studied include: blood lipids, lipoprotein cholesterols, and apolipoproteins; plasma haemostatic factors; blood chemistries and haematology; sitting, supine and standing blood pressures; anthropometry; fasting blood glucose and insulin levels; ECG findings; cigarette and alcohol use; physical activity levels; dietary aspects; and family history. (https://biolincc.nhlbi.nih.gov/studies/aric/)<br>https://www2.cscc.unc.edu/aric/
ATTICA[33,34]	Greece	The ATTICA is a health and nutrition prospective cohort study of 3,042 men and women participants (aged 18+, 50% men) that was established in 2002 in the metropolitan area of Athens, Greece. The sampling was random, multistage and was based on the age and sex distribution of the province of Attica provided by the National Statistical Service, according to the census of 2001. Only one participant per household was enrolled. After the baseline, there were two follow-up examinations. In 2006, the ATTICA study's investigators performed the intermediate 5-year follow-up and during 2011-12 they performed the 10-year follow-up.<br>https://www.maelstrom-research.org/mica/individual-study/attica#/population-ATTICA
British Regional Heart Study (BRHS)[35]	Great Britain	The British Regional Heart Study (BRHS) is a prospective study in middle-aged men (40-59 years) drawn from general practices in 24 British towns (recruited in 1978-1980). The study was set up to determine the factors responsible for the considerable variation in coronary heart disease, hypertension, and stroke in Great Britain. It also seeks to determine the causes of these conditions in order to provide a rational basis for recommendations towards their prevention. The 20 year re- examination, used for current analyses, took place between 1998 and 2000 when the men were aged 60-79 years.<br>https://www.ucl.ac.uk/iehc/research/primary-care-and-population-health/research/brhs
Cardiovascular Health Study (CHS)[36]	USA	The Cardiovascular Health Study (CHS; NCT00005133) is a study of risk factors for development and progression of CHD and stroke in people aged 65 years and older. The objectives of the Cardiovascular Health Study are to: 1) quantify associations of conventional and hypothesized risk factors with CHD and stroke; 2) assess the associations of non-invasive measures of subclinical disease with the incidence of CHD and stroke; 3) quantify the associations of risk factors with subclinical disease; 4) characterize the natural history of CHD and stroke, and identify factors associated with clinical course; and 5) describe the prevalence and distributions of risk factors, non-invasive measures of subclinical disease, and clinical CHD and stroke. 5,888 study participants were recruited from four U.S. communities and have undergone extensive clinic examinations for evaluation of markers of subclinical cardiovascular disease. The original cohort totalled 5,201 participants. A new cohort was recruited in 1992. The 687 participants in the new cohort are predominately African-American and were recruited at three of the four field centres. The 2,962 women and 2,239 men were examined yearly from 1989 through 1999. The added minority cohort of 256 men and 431 women was examined from 1992 to 1999. Examination components have

		included medical history questionnaires, measurement of ankle-brachial index, abdominal and carotid ultrasound studies, echocardiograms, ambulatory electrocardiograms, cerebral magnetic resonance imaging, spirometry, and retinal photographs over the past decade. The most extensive evaluations were at study entry (baseline) and again in 1992-1993 to assess change in subclinical disease measures. CHS has undertaken extensive follow-up for ascertainment of cardiovascular events including incident claudication, myocardial infarction, congestive heart failure, stroke and death. This manuscript was prepared using data obtained from the National Heart, Lung, and Blood Institute (NHLBI) Biologic Specimen and Data Repository Information Coordinating Center (BioLINCC) for the CHS and does not necessarily reflect the opinions or views of the CHS or NHLBI. (https://biolincc.nhlbi.nih.gov/studies/chs/) https://chs-nhlbi.org/
Dallas Heart Study (DHS)[37]	USA	The Dallas Heart Study (DHS) was initiated in 2000 with funding from the Donald W. Reynolds Foundation and with the primary goal of improving the diagnosis, prevention, and treatment of heart disease. From 2000 to 2002, 6,000 residents of Dallas County, aged 18 to 65 years, completed a detailed medical survey. Participants over the age of 30 (N=3,500) were invited to provide a blood sample and to undergo comprehensive state-of-the-art imaging studies to assess plaque buildup in the blood vessels of the heart, the size and function of the heart, and the amount and distribution of body fat.<br>The DHS was transformed from a cross-sectional to longitudinal study in 2007. All prior DHS participants were invited to return to the clinic for repeat evaluation to determine who had developed cardiovascular disease in the seven-year interval since the initial survey. The study is now supported by the Hoffman Family Center in Genetics and Epidemiology, directed by Dr. Helen Hobbs, and the National Center for Advancing Translational Sciences (NCATS). The DHS participants continue to be followed for the development of cardiovascular and metabolic disease through annual surveys and collaboration with hospitals in the DFW metroplex.<br>https://www.utsouthwestern.edu/research/translational-medicine/doing-research/dallas-heart/
DETECT[38]	Germany	The “Diabetes Cardiovascular Risk Evaluation Targets and Essential Data for Commitment of Treatment” (DETECT) trial is a cross-sectional clinical-epidemiological study with a prospective-longitudinal component in a nationally representative sample. The baseline study consisted of a nationwide representative sample of doctors with primary care functions (medical practitioners, general practitioners, general internists) in 3,188 primary care offices in Germany. Within this study cohort, a representative partial sample of 7,519 subjects, randomly selected in 1,000 primary care offices, underwent additional laboratory tests and was evaluated for a 5-year time period. The DETECT survey received the approval of the Ethics Committee of the Carl Gustav Carus Medical Faculty at the Technical University of Dresden (AZ EK149092003; Date 16.09.2003), and was registered at clinicaltrials.gov (NCT01076608). The following endpoints were reported by the physician during the 5-year follow-up: all-cause mortality, mortality of cardiovascular cause, occurrence of a myocardial infarction, and manifestation of CAD as evidenced by the necessity for coronary revascularization by either bypass graft (CABG) surgery or percutaneous coronary intervention (PCI). All information on end points was taken from a standardized assessment form by the primary care physician or the institution in which the patient was previously treated. Further information about causes of death from the death registry was taken into account. Fasting blood samples were collected and shipped by courier within 24 h to the central laboratory at the Medical University of Graz (Austria). Upon arrival, the samples were centrifuged immediately and serum was stored at -20°C until further processing. Clinical chemical parameters as well as cholesterol, triglycerides and lipoprotein (a) [Lp(a)] were determined on a Roche Modular automatic analyser. Lipoproteins (HDL, LDL and VLDL) were determined electrophoretically on the HELENA SAS-3/SAS-4 system. For all parameters, reagents and secondary standards were used as recommended by the manufacturers.
Dubbo Study of the elderly[39]	Australia	The Dubbo Study is an ongoing prospective study of a cohort of elderly people first examined in 1988–1989 in Dubbo, New South Wales. The study population were non-institutionalised subjects, comprising 1237 males and 1568 females 60 years and over. Methods and measures have been described in detail elsewhere.[39,40]
Framingham Heart Study (FHS)[41]	USA	The objectives of the Framingham Study (NCT00005121) are to study the incidence and prevalence of cardiovascular disease (CVD) and its risk factors, trends in CVD incidence and its risk factors over time, and familial patterns of CVD and risk factors. Other important objectives include the estimation of incidence rates of disease and description of the natural history of cardiovascular disease, including the sequence of clinical signs and systems that precede the clinically recognizable syndrome and the consequences and course of clinically manifest disease.<br>The Framingham Study began in 1948 under the U.S. Public Health Service and was transferred under the direct operations of the new National Heart Institute, NIH, in 1949. Participants were sampled from Framingham, Massachusetts, including both men and women. This was the first prospective study of cardiovascular disease and identified the concept of risk factors and their joint effects. The study has continued to examine participants every two years and is currently supported by a contract to Boston University from the NHLBI, and from many grants for specialized studies.<br>The Framingham Study is a longitudinal investigation of constitutional and environmental factors influencing the development of CVD in men and women.

		Examination of participants has taken place every two years and the cohort has been followed for morbidity and mortality over that time period. The cardiovascular disease conditions under investigation include coronary heart disease (angina pectoris, myocardial infarction, coronary insufficiency and sudden and non-sudden death), stroke, hypertension, peripheral arterial disease and congestive heart failure (https://biolincc.nhlbi.nih.gov/studies/framcohort/). https://www.framinghamheartstudy.org/
HAPIEE[42]	Eastern Europe	Health, Alcohol and Psychosocial factors in Eastern Europe (HAPIEE): The HAPIEE study comprises four prospective urban population based cohorts from Eastern Europe, including Novosibirsk (Russia), Krakow (Poland), Kaunas (Lithuania), and two cities of the Czech Republic. Each cohort recruited a random sample of men and women aged 45-69 years at baseline in May 2002 (August 2006 in Lithuania), stratified by sex and 5-year age group. Participants were selected from population registers (electoral roll list in Russia). Participants completed extensive questionnaire, underwent examination in clinic and provided a blood sample. The sample size (response rates) were 9,360 (61%) in Russia; 10,728 (61%) in Poland; 7,161 (61%) in Lithuania; and 8,857 (55%) in the Czech Republic. Deaths in the cohorts were identified by linkages with national or regional death registers. This dataset used as validation cohort study have only fatal endpoints. Follow-up is completed to 2011 for the Czech Republic and Lithuania, to 2010 for Russia and to 2009 for Poland. http://www.ucl.ac.uk/easteurope/hapiee.html
Health 2000/2011[43]	Finland	The Health 2000 Survey was carried out in Finland in 2000-2001. The study population was a two-stage stratified cluster sample representing the adult population living in mainland Finland. The Health 2000 sample comprised 8028 persons aged 30 or older of whom 87% participated in the home health interview and 84% in the comprehensive health examination. The purpose of the survey was to provide an up-to-date account of major public health problems in Finland, their causes and treatment, as well as functional capacity and work ability in the population.[43] For current analyses, we used data of N=7,384 individuals including fatal and non-fatal cardiovascular endpoints. https://thl.fi/en/web/thlfi-en/research-and-expertwork/projects-and-programmes/health-2000-2011/publications
Heinz Nixdorf RECALL Study (HNRS)[44]	Germany	The Heinz Nixdorf RECALL (Risk Factors, Evaluation of Coronary Calcification, and Lifestyle) Study is a population-based study in the large, heavily industrialized Ruhr area, Germany. From December 2000 to August 2003 random samples of men and women aged 45-74 were drawn from mandatory residency lists of three cities in het Ruhr area of North-western Germany (Essen, Mülheim and Bochum). Participants were invited via letter, and a maximum of two reminder letters and phone calls were made to the initial non-responders. Eligible for participation were all subjects without cardiovascular disease willing to participate and without any conditions which precluding follow-up over 5 years, without current pregnancy, and without severe psychiatric illness. Aim of the study was the evaluation of an improved risk prediction by coronary calcium, as a sign of subclinical coronary atherosclerosis, for cardiovascular events in comparison to traditional and new risk factors. Vital status in subjects not reached by yearly follow-up is extracted from mandatory citizen registries. For all possible primary study endpoints, hospital and nursing home records including electrocardiograms, laboratory values, and pathology reports were collected. For deceased subjects, death certificates were collected and interviews with general practitioners, relatives and eyewitnesses were under taken if possible. Medical records were obtained in 100% of all reported endpoints. An external criteria and endpoint committee blinded for conventional risk factor status and CAC scores reviewed all available documents of possible primary endpoints and classified the endpoints thereafter. Cardiovascular endpoints were defined as follows: Acute myocardial infarction according to the WHO MONICA diagnostic categories derived from symptoms, signs of electrocardiography, and enzymes (levels of creatine kinase (CK-MB)) as well as troponin T or I, and necropsy as (1) non-fatal acute myocardial infarction and (2) coronary death, which occurred after the baseline examination. Resuscitated Cardiac Arrest: Physician or emergency physicians documented fatal or non-fatal cardiac arrest with resuscitation. Stroke (not TIA): Rapidly developing focal neurologic symptoms lasting at least 24 hours or until death due to no other than vascular cause. CHD Death: Death related to coronary heart disease documented in hospital records and confirmed by the event committee unrelated to any time intervals. https://www.uni-due.de/recall-studie/die-studien/hnr/
HUNT[45]	Norway	The Nord-Trøndelag Health Study (Helse Undersøkelsen i Nord-Trøndelag [Norwegian]: HUNT) was initiated in the 1980s as a health survey that addressed four main topics: hypertension, diabetes, quality of life, and tuberculosis as well as other lung diseases. Today, the HUNT study includes 125,000 Norwegian participants from four surveys (HUNT 1 1984-86; HUNT 2 1995-97; HUNT 3 2006-08, and HUNT 4 2017-2019). Every citizen of Nord-Trøndelag County in Norway aged 20 years or older, has been invited to all the surveys for adults. Participants may be linked in families and followed up longitudinally between the surveys and in several national health- and other registers covering the total population. The HUNT Study includes data from questionnaires, interviews, clinical measurements and biological samples (blood and urine). The questionnaires included questions on socioeconomic conditions, health related behaviours, symptoms, illnesses and diseases. For the current analyses data from 9,557 participants in the HUNT 2 cohort was used. https://www.ntnu.edu/hunt

Jackson Heart Study (JHS)[46]	USA	The objectives of the Jackson Heart Study (NCT00005485) are to: 1) investigate the associations of biological, psychosocial, and behavioral factors with the incidence atherosclerotic events and health outcomes in an African American cohort; and 2) increase access to and the participation of African American populations and scientists in biomedical research and professions.<br>Participants were enrolled in the study from 2000-2004 from urban and rural areas of the three counties (Hinds, Madison and Rankin) that make up the Jackson MS, metropolitan statistical area (MSA). Participants were enrolled from each of 4 recruitment pools: a random sample component (17%), volunteer component (30%), currently enrolled in the Atherosclerosis Risk in Communities (ARIC) Study (31%), and secondary family members (22%). Recruitment was limited to non-institutionalized adult African Americans 35-84 years old, except in the family cohort where those 21 to 34 years of age were eligible. The final cohort of 5,301 participants includes 6·59% of all African American Jackson MSA residents aged 35-84 (N-76,426, US Census 2000). Data collection at the baseline exam included a medical history, physical examination, blood/urine analyses and interview questions on areas such as: physical activity; stress, coping and spirituality; racism and discrimination; socioeconomic status; and health care access.<br>The current release of the Jackson Heart Study includes data collected at the baseline and visit 2 examinations. Jackson is an ongoing study and Eighty-two percent of the surviving JHS participants (N = 4203) completed Exam 2, and projected retention for Exam 3 is 80% (N = 4082). Annual cohort follow-up of the cohort for incident clinical events of interest is ongoing. (https://biolincc.nhlbi.nih.gov/studies/jhs/)<br>https://www.jacksonheartstudy.org/
Malmö Diet and Cancer Study (MDCS)[47]	Sweden	The Malmö Diet and Cancer Study (MDCS) was original initiated to clarify whether a western diet is associated with certain forms of cancer whilst taking other life-style factors into account. Individuals aged 45-69 years living in Malmö city, Sweden, were eligible to participate**.** From 1991-1994, every other participant was invited to also take part in a substudy of the epidemiology of carotid artery disease. A total of 6,103 subjects accepted the invitation and where rescheduled for fasting blood sampling under standardized conditions. Risk factors were estimated on the basis of laboratory tests, baseline examinations, and through the questionnaire administered at the baseline visit. Information regarding smoking habits, physical activity, medical history, and use of medication was based on the self-administered questionnaire.[48] The study was approved by the Ethics Committee at Lund University. Each participant gave his or her written informed consent.
Malmö Prevention Project (MPP)[49]	Sweden	The MPP was started in the early 70's as a screening survey in the middle-aged population of Malmö, the third largest city of Sweden. Subjects born in Malmö and residents of the city were invited for a clinical examination, questionnaire, and blood sampling. In all 22,444 men and 10,902 women participated during the period 1974-1992. During a later follow-up, the MPP-Re-examination (MPP-RES) in all 17,284 of the original screens attended in 2002-2006 (https://snd.gu.se/en/catalogue/study/ext0013).<br>https://www.med.lu.se/malmoe_kost_cancer_och_malmoe_foerebyggande_medicin
Melbourne Collaborative Cohort Study (MCCS)[50]	Australia	The Melbourne Collaborative Cohort Study is a longitudinal study established in the 1990s by Cancer Council Victoria to investigate prospectively the role of diet and other lifestyle factors in cancer. Between 1990 and 1994, 41,500 people (24,500 women and 17,000 men) aged 40 to 69 were recruited into the study. Approximately one third of participants are southern European migrants to Australia, who were deliberately over-sampled to extend the range of data on lifestyle exposures and to increase genetic variation. At baseline, lifestyle exposure information, including dietary intake, was collected in a face-to-face interview. Physical measurements and blood pressure were also taken. A sample of blood was drawn and stored for analysis of DNA and other molecules of interest. Follow-up was conducted by mailed questionnaire and telephone to update lifestyle exposures and self-reports of non-cancer, non-fatal health events at 3 to 4 years after baseline. During 2003-2006, approximately 27,000 cohort participants attended the study centre to repeat the baseline measures and health survey. Follow-up is continuing. The study's main focus has been on identifying risk factors for cancer and other chronic diseases, such as type 2 diabetes, cardiovascular disease, eye disease, and arthritis. Through data collected from this contemporary large cohort study, the investigators are studying the determinants of chronic disease, with the aim of developing prediction tools applicable to the current Australian population. Results from this study will allow future patterns of chronic disease to be accurately forecasted, which in turn permits preventive strategies to be used in a more effective manner. The MCCS does not have incident CVD variables.<br>(https://epi.grants.cancer.gov/Consortia/members/melbourne.html).<br>https://www.cancervic.org.au/research/epidemiology/health_2020/health2020-overview
Multi-Ethnic Study of Atherosclerosis (MESA)[51]	USA	The Multi-Ethnic Study of Atherosclerosis (MESA) (NCT00005487) is a population-based longitudinal study of 6,800 ethnically diverse men and women free of clinical cardiovascular disease at baseline in 2000. MESA is investigating the prevalence, correlates, and progression of subclinical CVD and risk factors that predict progression to clinically overt CVD, and that predict progression of subclinical disease itself, with on-going follow-up of the cohort. Baseline measurements included coronary calcium using computed tomography; ventricular mass and function using

		cardiac magnetic resonance imaging; flow-mediated brachial artery endothelial vasodilation, carotid intimal-medial wall thickness, and distensibility of the carotid arteries using ultrasonography; peripheral vascular disease using ankle and brachial blood pressures; electrocardiography; and assessments of microalbuminuria, standard CVD risk factors, sociodemographic factors, life habits, and psychosocial factors. Participants are followed actively for identification and characterization of incident CVD events, including acute myocardial infarction and other coronary heart disease, stroke, peripheral vascular disease, and congestive heart failure; therapeutic interventions for CVD; and mortality. (https://biolincc.nhlbi.nih.gov/studies/mesa/) https://www.mesa-nhlbi.org/
PREVEND Study[52]	The Netherlands	The PREVEND (Prevention of REnal and Vascular ENd stage Disease) study is a general population-based prospective cohort study that was started in 1997 in the city of Groningen, The Netherlands. For this study all the inhabitants of the city of Groningen were asked, in 1997, to complete a brief questionnaire and provide morning urine. Of the 41,000 respondents, 8,600 persons were immediately invited for further examination. The PREVEND study was approved by the Medical Ethics Committee of the University of Groningen. Informed written consent was obtained from all participants. The study adhered to the ethical principles set by the Declaration of Helsinki.[53] https://www.umcg.nl/EN/Research/Researchers/Facilities/biobanks/biobanks/prevend/Paginas/default.aspx#
Rotterdam Study[54]	The Netherlands	The Rotterdam Study is a prospective cohort study ongoing since 1990 in the well-defined Ommoord district in the city of Rotterdam in The Netherlands. The study targets cardiovascular, endocrine, hepatic, neurological, ophthalmic, psychiatric, dermatological, otolaryngological, locomotor, and respiratory diseases. The Rotterdam Study initially included 7,983 persons (78% of 10,215 invitees) of 55 years of age or older (Rotterdam Study I). In 2000, 3,011 participants (out of 4,472 invitees) who had become 55 years of age or moved into the study district since the start of the study were added to the cohort (Rotterdam Study II). In 2006, a further extension of the cohort was initiated in which 3,932 subjects aged 45–54 years (out of 6,057 invited) were included (Rotterdam Study III). By the end of 2008, the Rotterdam Study therefore comprised 14,926 subjects aged 45 years or over. The overall response figure for all three cycles at baseline was 72·0% (14,926 of 20,744). For current analyses data of 10,057 individuals were available. The Rotterdam Study has medical ethics committee approval per the Population Study Act: Rotterdam Study, executed by the Ministry of Health, Welfare and Sport of the Netherlands. Written informed consent was obtained from all participants. http://www.epib.nl/research/ergo.htm
ULSAM Study[55,56]	Sweden	In the county of Uppsala, Sweden, all men born between 1920 and 1924 were invited to participate at the Uppsala Longitudinal Study of Adult Men (ULSAM) at age 50. Aim of this study, performed from 1970 to 1973 was to identify risk factors for cardiovascular disease. 82% of the invited men participated (N=2,322). The design and selection criteria for the cohort have been described previously.[55] For the current analysis we could include N=2,310 men. ULSAM participants were re-examined at age 60, 70, 77, and 82, 88, and 93 with a median follow-up of 30 years. Uppsala University Ethics Committee approved the study and informed written consent was obtained from study participants.[56] http://www.pubcare.uu.se/ulsam/

Affiliations of the consortium members

University Heart & Vascular Center Hamburg, Hamburg, Germany (Fabian J. Brunner, MD; Christoph Waldeyer, MD; Francisco Ojeda, PhD; Stefan Blankenberg, Prof, MD; Tanja Zeller, Prof, PhD; Thiess Lorenz, MA; Nataliya Makarova, PhD); National Institute for Health and Welfare (THL), Helsinki, Finland (Veikko Salomaa, Prof, MD; Kari Kuulasmaa, Prof, PhD; Jukka Kontto, M.Soc.Sc.; Satu Männistö, PhD; Tuija Jääskeläinen, PhD; Teemu Niiranen, MD; Pekka Jousilahti, Prof, MD); Centre for Public Health (NI), Queens University of Belfast, Belfast, Northern Ireland (Frank Kee, Prof, MD); Catalan Department of Health, Barcelona, Spain (Susana Sans, MD); Institute of Epidemiology, Helmholtz Zentrum München, German Research Center for Environmental Health, Neuherberg, Germany (Barbara Thorand, Prof, Ph.D; Annette Peters, Prof, PhD; Christa Meisinger, Prof, MD); Department of Cardiovascular, Endocrine-metabolic Diseases and Aging, National Institute of Health-ISS, Rome, Italy (Simona Giampaoli, MD); Department of Medicine and Surgery, University of Milano-Bicocca, Italy (Paolo Brambilla, MD; Stefano G. Signorini, MD); Cardiovascular Epidemiology Unit, Institute of Cardiovascular Research, University of Dundee, Dundee, United Kingdom (Hugh Tunstall-Pedoe, Prof, MD); Department of Epidemiology and Public health, University Hospital of Strasbourg, Strasbourg, France (Marie Moitry, MD); Department of Epidemiology and Prevention. IRCCS Neuromed. Pozzilli, Italy (Licia Iacoviello, Prof, PhD; Simona Costanzo, PhD); Research Center in Epidemiology and Preventive Medicine (EPIMED), Department of Medicine and Surgery, University of Insubria, Varese, Italy (Licia Iacoviello, Prof, PhD; Giovanni Veronesi, PhD; Francesco Gianfagna, PhD); Clinica Medica, Department of Medicine and Surgery, University of Milano Bicocca, Milan, Italy (Guido Grassi, Prof, MD); Department of Clinical Medicine, UiT The Arctic University of Norway, Tromsø, Norway and Department of Neurology, University Hospital of North Norway, Tromsø, Norway (Ellisiv B Mathiesen, Prof, MD); Department of Public Health and Clinical Medicine, and Heart Center, Cardiology, Umeå University, Umeå, Sweden (Stefan Söderberg, Prof, PhD); Center for Clinical Research and Prevention, Bispebjerg and Frederiksberg Hospital, Copenhagen, Denmark (Allan Linneberg, Prof, PhD; Torben Jørgensen, Prof, MD); Department of Clinical Medicine, Faculty of Health and Medical Sciences, University of Copenhagen, Copenhagen, Denmark (Allan Linneberg, Prof, PhD); Division of Clinical Epidemiology and Aging Research C070 German Cancer Research Center (DKFZ) Heidelberg Germany (Hermann Brenner, Prof, MD; Ben Schöttker, PhD); Univ. Lille, Inserm, Centre Hosp. Univ Lille, Institut Pasteur de Lille, UMR1167 - RID-AGE - Risk factors and molecular determinants of aging-related diseases, Epidemiology and Public Health Department, France (Philippe Amouyel, Prof, MD; Jean Dallongeville, MD); Toulouse University School of Medicine, Toulouse, France (Jean Ferrieres, Prof, MD); Institute of Cardiology, Lithuanian University of Health Sciences, Kaunas, Lithuania (Abdonas Tamosiunas, Prof, MD); Research Institute of Internal and Preventive Medicine - Branch of Federal Research Center, Institute of Cytology and Genetics, Siberian Branch, Russian Academy of Sciences, Novosibirsk, Russia (Yuriy P. Nikitin, Prof, PhD; Sofia Malyutina, PhD); Department of Epidemiology, CVD Prevention and Health Promotion, National Institute of Cardiology, Warsaw, Poland (Wojciech Drygas, Prof, PhD); Department of Clinical Sciences, Malmö, Lund University, Malmö, Sweden (Olle Melander, Prof, MD; Gunnar Engström, Prof, MD); Institute for Medical Informatics, Biometry and Epidemiology, University Hospital of Essen (Karl-Heinz Jöckel, Prof, PhD; Börge Schmidt, PhD); Department of Cardiology, Charité Berlin - University Medicine, Campus Benjamin Franklin, Berlin, Germany (David M. Leistner, MD; Ulf Landmesser, Prof, MD); DZHK (German Centre for Cardiovascular Research), Partner Site Berlin, and Berlin Institute of Health (BIH), Berlin, Germany (David M. Leistner, MD; Ulf Landmesser, Prof,

MD); Baker Heart and Diabetes Institute, Melbourne, Victoria, Australia (Jonathan E. Shaw, Prof, MD; Dianna J. Magliano, Prof, PhD); Department of Nutrition and Dietetics, School of Health Science and Education, Harokopio University, Athens, Greece (Demosthenes B. Panagiotakos, Prof, DrMed.); University Of NSW and St Vincent's Hospital, Australia (Leon A. Simons, Prof, MD); Department of Epidemiology, Erasmus MC, University Medical Center Rotterdam, Rotterdam, The Netherlands (Maryam Kavousi, PhD; M. Kamran Ikram, Prof, PhD); Boston University and the National Heart, Lung, and Blood Institute's Framingham Study, Framingham, MA, United States (Ramachandran S. Vasan, Prof, MD; Emelia J. Benjamin, Prof, MD); Department of Endocrinology, University Medical Center Groningen, University of Groningen, Groningen, The Netherlands (Robin P.F. Dullaart, MD); Primary Care and Population Health, University College London, London, United Kingdom (S. Goya Wannamethee, Prof, PhD); Department of Public Health and Caring Sciences, Clinical and Nutrition and Metabolism Unit, Uppsala University, Uppsala, Sweden (Ulf Risérus, Prof, PhD); Departments of Medicine and Epidemiology, Columbia University, New York, NY, United States (Steven Shea, Prof, MD); Division of Cardiology, University of Texas Southwestern Medical Center, Dallas TX, United States (James A. de Lemos, Prof, MD); Department of Cardiology, Division of Medicine, Akershus University Hospital, Lørenskog, Norway and Center for Heart Failure Research, Institute of Clinical Medicine, University of Oslo, Oslo, Norway (Torbjørn Omland Prof, MD; Magnus N. Lyngbakken, MD); Berlin Institute of Health (BIH), Berlin, Germany (Ulf Landmesser, Prof, MD); DZHK (German Center for Cardiovascular Research), Partner Site Hamburg/Kiel/Lübeck, Hamburg, Germany (Stefan Blankenberg, Prof, MD; Tanja Zeller, Prof, PhD); Institute of Genomics, University of Tartu, Tartumaa, Estonia (Andres Metspalu, Prof, MD; Maris Alver, MD); Institute for Clinical Chemistry and Laboratory Medicine, University Medical Center Mainz, Mainz, Germany (Karl Lackner, Prof, MD); Preventive Cardiology and Preventive Medicine, Center for Cardiology, University Medical Center, Johannes Gutenberg-University Mainz, Mainz, Germany (Philipp Wild, MD); Ludwig-Maximilians-Universität München, Chair of Epidemiology, UNIKA-T, Augsburg, Germany and Helmholtz Zentrum München, German Research Center for Environmental Health, Research Unit Clinical Epidemiology, Neuherberg, Germany (Christa Meisinger, Prof, MD); Cerebro and Cardiovascular Epidemiology Unit, National Centre of Epidemiology, Surveillance, and Promotion of Health, National Institute of Health, Rome, Italy (Chiara Donfrancesco, PhD); The George Institute for Global Health, University of Oxford, United Kingdom, and University of New South Wales, Australia (Mark Woodward, Prof, MD); Mediterranea Cardiocentro, Napoli, Italy (Francesco Gianfagna, PhD); Department of Community Medicine, UiT The Arctic University of Norway, Tromsø, Norway (Tom Wilsgaard, Prof, PhD); Department of Public Health and Clinical Medicine, Sunderby Research Unit, Umeå University, Sweden (Mats Eliasson, Prof, PhD); Institute for Community Medicine, University Medicine Greifswald, Greifswald, Germany (Henry Völzke, Prof, MD); DZHK (German Centre for Cardiovascular Research), partner site Greifswald, Greifswald, Germany (Henry Völzke, Prof, MD; Marcus Dörr, Prof, MD; Matthias Nauck, Prof, MD); Department of Internal Medicine B, University Medicine Greifswald, Greifswald, Germany (Marcus Dörr, Prof, MD); Institute of Clinical Chemistry and Laboratory Medicine, University Medicine Greifswald, Greifswald, Germany (Matthias Nauck, Prof, MD); Cardiovascular Research Institute Basel (CRIB) and Department of Cardiology, University Hospital Basel, University of Basel, Basel, Switzerland (Raphael Twerenbold, MD); School of Public Health, University of Queensland, Herston, Brisbane, Australia (Annette Dobson, Prof, PhD); Department of Epidemiology and Population Studies, Faculty of Health Sciences, Jagiellonian University Medical College, Krakow, Poland (Andrzej Pajak, Prof, PhD); Department of Epidemiology and Public Health, University College London,

London, United Kingdom (Martin Bobak, Prof, PhD); Department of Medicine, Turku University Hospital and University of Turku, Turku, Finland (Teemu Niiranen, MD); Centre for Epidemiology and Biostatistics, Melbourne School of Population and Global Health, The University of Melbourne, Melbourne, Australia (Graham Giles, Prof, PhD; Allison Hodge, Prof, PhD); German Rheumatism Research Center Berlin, Berlin, Germany; Institute for Epidemiology and Health Care Economics, Charité Berlin - University Medicine, Campus Mitte, Berlin, Germany (Jens Klotsche, Prof, PhD); K. G. Jebsen Center for Genetic Epidemiology, Dept of Public Health and Nursing, Faculty of Medicine and Health Sciences, NTNU, Norway and the HUNT Research Center, Dept of Public Health and Nursing, Faculty of Medicine and Health Sciences, NTNU, Norway (Kristian Hveem, Prof, PhD); First Cardiology Clinic, School of Medicine, University of Athens, Athens, Greece (Christos Pitsavos, Prof, MD); Department of Nephrology, University Medical Center Groningen, University of Groningen, Groningen, The Netherlands (Stephan J.L. Bakker, Prof, MD); Population Health Research Institute, St George's, University of London, London, United Kingdom (Peter Whincup, Prof, PhD); Department of Public Health and Caring Sciences, Section of Geriatrics, Uppsala University, Sweden (Martin Ingelsson, Prof, MD); Deutsches Herzzentrum München, Technische Universität München, Munich, Germany and German Center for Cardiovascular Research (DZHK e.V.), partner site Munich Heart Alliance, Munich, Germany (Wolfgang Koenig, Prof, MD); Institute of Epidemiology and Medical Biometry, University of Ulm, Ulm, Germany (Wolfgang Koenig, Prof, MD)

Online-only methods

Detailed definition of cardiovascular risk factors

Daily Smoking: The risk factor of daily smoking was assessed as current cigarette smoker (ARIC, BRHS, CHS, Dubbo, FHS Offspring, HAPIEE, MPP, PREVEND, ULSAM, DHS, SHIP), as current regular smoker (MDC), current smoker smoking 7 or more cigarettes per week (MCCS), or using the MORGAM definition of daily smoker of one cigarette or more (ATTICA, AusDiab, DETECT, FHS Gen3, HUNT, JHS, MESA, Health2000).

Diabetes: The risk factor diabetes was defined by the diagnosis of a physician (HAPIEE, Health2000), self-reported or by history of diabetes according to MORGAM (BRHS, DETECT, Dubbo, HRRS, HUNT), self-reported history of diabetes (DHS, MPP), definition by the American Diabetes Association (CHS, JHS, MESA), definition given by ICD-10 coding (ATTICA), definition based on blood glucose level or intake of antidiabetic medication (ARIC, FHS Offspring, FHS Gen3), diagnosed diabetes or intake of antidiabetic medication (AusDiab), self-reported and intake of antidiabetic medication (PREVEND), self-reported diabetes and blood glucose levels (MCCS), blood glucose levels and acute insulin response (ULSAM), diagnosed diabetes by a physician in combination with specific measures, including specific diet as well as elevated HbA1c and blood glucose levels (SHIP).

Hypertension: Hypertension was defined according to the WHO guidelines as systolic blood pressure above 140 mmHg or intake of antihypertensive medication.

Obesity: Obesity was defined according to WHO guidelines as a BMI of greater than or equal to 30.

Detailed definition of endpoints

Cardiovascular disease event as an endpoint was defined as the first fatal or non-fatal coronary heart disease event or likely cerebral infarction.

The coronary event included:

- acute definite or possible myocardial infarction or coronary death
- unstable angina pectoris
- cardiac revascularization
- unclassifiable death

"Unclassifiable" death refers to death with insufficient evidence of coronary origin and no competing cause. For further details of the definition, see MORGAM Derived Variable CVD3.[57]

In the **MONICA/KORA** Augsburg study, cardiac revascularization was not followed up. In the MONICA/KORA Augsburg and **MONICA Brianza** studies unstable angina pectoris was not assessed as an outcome but it is largely included in the category "possible myocardial infarction" of the WHO MONICA classification used in these studies. Likewise, the **PRIME Belfast** study did not include possible myocardial infarction, but this is largely compensated by unstable angina pectoris which was assessed. The follow-up starts at the date of baseline examinations. Details of the follow-up and diagnostic procedures of each participating study have been published elsewhere.[57]

The definition of the composite cardiovascular event in the **Atherosclerosis Risk in Communities Study** was based on definite or probable incident stroke, myocardial infarction, silent myocardial infarction and fatal coronary heart disease and by censoring date.

The **ATTICA** study variables are defined according to ICD-10.

The **AusDiab** study included the following diseases and coronary interventions for the definition of endpoint: first adjudicated myocardial infarction (primary or secondary definition), first fatal or non-fatal myocardial infarction (primary definition only), first fatal or non-fatal myocardial infarction (primary and secondary definition), first adjudicated coronary artery bypass surgery, first adjudicated coronary artery angioplasty or stent surgery, first fatal or non-fatal stroke (definite only), first fatal or non-fatal stroke (definite and possible), first adjudicated stroke (definite only), first adjudicated stroke (definite and possible combined).

The **British Regional Heart Study** used for the definition of endpoint fatal, non-fatal and non-fatal followed by fatal myocardial infarction and fatal, non-fatal and non-fatal followed by fatal stroke.

The **Cardiovascular Health Study** incorporated myocardial infarction, angina pectoris, angioplasty, coronary artery bypass surgery, electrocardiography based myocardial infarction (silent), stroke and fatal coronary heart disease when defining composite endpoint.

The **DETECT** trial harmonized its variables according to the definitions of MORGAM/BiomarCaRE.

The **Dallas Heart Study** used myocardial infarction, unstable angina, and stroke for the definition of endpoint.

The **Dubbo** study attempted to harmonize its variables close to definitions of MORGAM/BiomarCaRE. As the Dubbo study does not have all the variables for myocardial infarction, they provided coronary heart diseases and

stroke variables close to those from MORGAM/BiomarCaRE consortium and variables that could be used if the event was myocardial infarction.

The **Framingham Heart Offspring Study** and the **Framingham Generation 3** study used following endpoints by defining the major cardiovascular event:

- Coronary heart disease including myocardial infarction recognized, with diagnostic electrocardiography, myocardial infarction recognized, without diagnostic electrocardiography, with enzymes and history, myocardial infarction recognized, without diagnostic electrocardiography, with autopsy evidence, myocardial infarction unrecognized, silent, myocardial infarction unrecognized, not silent angina pectoris, first episode only, cardiac insufficiency, definite by both history and electrocardiography, questionable myocardial infarction, sudden death caused by coronary heart disease within 1 hour, non-sudden death caused by coronary heart disease between 1 and 23 hours, non-sudden death caused by coronary heart disease between 24 and 47 hours, non-sudden death caused by coronary heart disease after 48 hours or more.

- Congestive heart failure including not hospitalized, diagnosed on the basis of a medical examination or physician’s notes, hospitalized and questionable disease.

- Stroke including definite cerebrovascular accident, but questionable type, atherothrombotic infarction of the brain, cerebral embolism, intracerebral hemorrhage, subarachnoid hemorrhage, other cerebrovascular accident, questionable cerebrovascular accident;

- Cardiovascular disease includes in addition to coronary heart disease, congestive heart failure and stroke variables, acute myocardial infarction by autopsy, transient ischemic attack, definite cerebrovascular accident of unknown type, death caused by cerebrovascular accident, death caused by other cardiovascular disease, death by any other cause, cardiac insufficiency, first episode only and questionable cardiac insufficiency

For the **HAPIEE** study only fatal cardiovascular disease was available.

The **Health 2000/2011 study** used fatal and non-fatal CHD events as well as fatal and non-fatal ischaemic stroke events as endpoints.

The **Heinz Nixdorf RECALL study** used definitive fatal and nonfatal myocardial infarction, possible fatal CHD, definitive fatal CHD, possible non-fatal myocardial infarction, and stroke.

The **HUNT** study included myocardial infarction and stroke in the definition of composite endpoint. Cardiac revascularization and unstable angina pectoris were not assessed as an outcome.

The **Jackson Heart Study** used only ischemic stroke and coronary heart disease with cardiac procedure when defining a major cardiovascular event.

For the **Melbourne Collaborative Cohort Study** only fatal information was available.

The **Multi-Ethnic Study of Atherosclerosis** included coronary heart disease and stroke in the definition of the composite endpoint: Myocardial infarction, resuscitated cardiac arrest, definite angina, probably angina if followed by revascularization, brain infarction. Death caused by coronary heart disease was also part of the composite endpoint as well as other and unknown stroke types (haemorrhage categories were excluded for current analyses).

In the **Malmö Diet and Cancer Study** and the **Malmö Prevention Project** definition of major coronary event was based on the history of incident coronary event and first occurrence of coronary event until last follow-up date (2014-12-31). ICD-9 codes were used to encode coronary events. These are: acute myocardial infarction, other acute and subacute forms of ischemic heart disease, old myocardial infarction, and other forms of chronic ischemic heart disease. Stroke was based on history of incident stroke and first incident stroke event until last follow-up date (2014-12-31).

The **PREVEND** study used for the definition of endpoint well defined cardiac events including fatal and non-fatal myocardial infarction, fatal and non-fatal ischemic heart disease, fatal and non-fatal coronary artery bypass grafting, fatal and non-fatal percutaneous transluminal coronary angioplasty and well defined peripheral events. Stroke was based on well-defined cerebrovascular event including fatal and non-fatal occlusion and stenosis of precerebral arteries, occlusion of cerebral arteries and fatal and non-fatal rupture of carotid artery.

The **Rotterdam** study defined the CHD endpoint as incidents of myocardial infarction, PCI or bypass graft and furthermore included CVD and death as endpoints.

The **Uppsala Longitudinal Study of Adult Men** included in the definition of major cardiovascular event ischaemic heart disease, acute myocardial infarction and stroke.

Online-only tables

Table S1: Baseline information – MORGAM/BiomarCaRE cohorts

	**ATBC (N=26,748)**	**MONICA Brianza I (N=1,640)**	**MONICA Brianza II (N=1,574)**	**MONICA Brianza III (N=1,639)**	**DanMONICA I (N=3,925)**	**DanMONICA II (N=1,471)**	**DanMONICA III (N=1,945)**	**ESTHER (N=9,131)**
Examination years, range	1984-1988 AV: 26748	1986-1987 AV: 1640	1989-1990 AV: 1574	1993-1994 AV: 1639	1982-1985 AV: 3925	1986-1987 AV: 1471	1991-1992 AV: 1945	2000-2003 AV: 9131
Examination age (years)	56·9 (53·3, 61·2) AV: 26748	45·8 (36·0, 55·2) AV: 1640	46·3 (37·1, 55·7) AV: 1574	46·9 (37·0, 56·2) AV: 1639	50·5 (40·5, 60·5) AV: 3925	41·0 (39·8, 50·9) AV: 1471	49·9 (39·8, 60·0) AV: 1945	62·3 (56·8, 66·8) AV: 9131
Age <45 years %	0 AV: 26748	47·4 AV: 1640	46·3 AV: 1574	44·4 AV: 1639	49·5 AV: 3925	50·4 AV: 1471	41·8 AV: 1945	0 AV: 9131
Age 45-59 years %	68·9 AV: 26748	40·5 AV: 1640	40·0 AV: 1574	40·0 AV: 1639	23·9 AV: 3925	27·7 AV: 1471	31·0 AV: 1945	37·2 AV: 9131
Age ≥60 years %	31·1 AV: 26748	12·1 AV: 1640	13·8 AV: 1574	15·6 AV: 1639	26·6 AV: 3925	21·9 AV: 1471	27·2 AV: 1945	62·8 AV: 9131
Female %	0 AV: 26748	51·2 AV: 1640	50·1 AV: 1574	52·0 AV: 1639	49·1 AV: 3925	50·6 AV: 1471	51·1 AV: 1945	57·0 AV: 9131
SCORE (%)	5·5 (3·5, 9·0) AV: 26713	0·4 (0·1, 1·7) AV: 1618	0·4 (0·1, 1·7) AV: 1520	0·4 (0·1, 1·8) AV: 1635	0·4 (0·1, 2·0) AV: 3920	0·4 (0·0, 1·6) AV: 1469	0·6 (0·1, 3·5) AV: 1930	3·4 (1·6, 6·2) AV: 2962
PCE (%)	AV: 0	2·4 (0·7, 6·8) AV: 1580	2·3 (0·6, 6·8) AV: 1510	2·4 (0·7, 6·8) AV: 1477	2·9 (0·9, 7·7) AV: 3741	2·7 (0·8, 6·5) AV: 1448	3·9 (1·0, 11·9) AV: 1862	12·9 (6·7, 21·6) AV: 1792
BMI (kg/m²)	25·9 (23·7, 28·5) AV: 26731	24·6 (22·2, 27·1) AV: 1628	25·0 (22·7, 27·9) AV: 1548	25·1 (22·5, 28·1) AV: 1623	24·2 (21·9, 26·9) AV: 3924	24·3 (22·1, 26·8) AV: 1466	24·7 (22·4, 27·7) AV: 1945	27·2 (24·7, 29·9) AV: 9116
Hypertension %	54·1 AV: 26744	37·2 AV: 1632	34·7 AV: 1543	31·2 AV: 1636	22·9 AV: 3925	16·3 AV: 1469	27·8 AV: 1943	53·2 AV: 8882
Diabetes %	3·9 AV: 26748	2·4 AV: 1604	3·0 AV: 1565	2·2 AV: 1498	2·2 AV: 3925	1·2 AV: 1471	2·9 AV: 1945	14·4 AV: 9121
Daily smoker %	100·0 AV: 26748	33·0 AV: 1639	28·7 AV: 1573	28·4 AV: 1638	46·6 AV: 3925	42·5 AV: 1471	42·2 AV: 1945	44·4 AV: 3081
Cholesterol lowering medication %	AV: 0	AV: 0	AV: 0	2·2 AV: 1627	AV: 0	AV: 0	0·4 AV: 1943	8·1 AV: 9055
LDL cholesterol (mmol/L)	AV: 0	3·3 (2·7, 4·0) AV: 1578	3·4 (2·7, 4·1) AV: 1533	3·6 (3·0, 4·3) AV: 1622	3·6 (2·9, 4·3) AV: 3875	3·7 (3·1, 4·5) AV: 1439	3·7 (3·1, 4·4) AV: 1915	3·8 (3·1, 4·6) AV: 5406
HDL cholesterol (mmol/L)	1·1 (1·0, 1·4) AV: 26748	1·4 (1·2, 1·6) AV: 1631	1·4 (1·2, 1·7) AV: 1556	1·4 (1·2, 1·7) AV: 1636	1·4 (1·2, 1·7) AV: 3920	1·4 (1·2, 1·7) AV: 1469	1·4 (1·1, 1·7) AV: 1934	1·3 (1·1, 1·6) AV: 5566
Non-HDL cholesterol (mmol/L)	5·0 (4·2, 5·8) AV: 26717	3·9 (3·2, 4·7) AV: 1628	3·9 (3·1, 4·7) AV: 1554	4·2 (3·5, 5·0) AV: 1635	4·2 (3·4, 5·0) AV: 3920	4·3 (3·5, 5·2) AV: 1469	4·3 (3·5, 5·1) AV: 1931	4·5 (3·7, 5·4) AV: 5529
Non-HDL cholesterol <2·6 mmol/L %	1·2 AV: 26717	8·1 AV: 1628	10·2 AV: 1554	4·4 AV: 1635	5·4 AV: 3920	4·8 AV: 1469	4·2 AV: 1931	7·6 AV: 5529
Non-HDL cholesterol 2·6 to <3.7 mmol/L %	12·2 AV: 26717	36·9 AV: 1628	35·5 AV: 1554	29·0 AV: 1635	29·9 AV: 3920	27·0 AV: 1469	27·0 AV: 1931	19·2 AV: 5529
Non-HDL cholesterol 3·7 to <4.8 mmol/L %	30·7 AV: 26717	32·5 AV: 1628	31·8 AV: 1554	35·4 AV: 1635	33·0 AV: 3920	32·3 AV: 1469	34·7 AV: 1931	31·9 AV: 5529
Non-HDL cholesterol 4·8 to <5.7 mmol/L %	29·0 AV: 26717	13·8 AV: 1628	15·4 AV: 1554	19·4 AV: 1635	18·6 AV: 3920	20·2 AV: 1469	20·3 AV: 1931	22·6 AV: 5529
Non-HDL cholesterol ≥5·7 mmol/L %	26·9 AV: 26717	8·7 AV: 1628	7·1 AV: 1554	11·9 AV: 1635	13·0 AV: 3920	15·7 AV: 1469	13·7 AV: 1931	18·8 AV: 5529
Incident CVD %	28·8 AV: 26748	9·8 AV: 1637	7·9 AV: 1574	5·0 AV: 1639	18·1 AV: 3925	14·7 AV: 1471	14·1 AV: 1945	2·3 AV: 8813
Death %	47·4 AV: 26748	16·2 AV: 1639	12·3 AV: 1574	6·3 AV: 1639	37·5 AV: 3925	23·5 AV: 1471	25·4 AV: 1945	10·3 AV: 9130

	ECGUT (N=1,959)	FINRISK 1982 (N=8,706)	FINRISK 1987 (N=5,471)	FINRISK 1992 (N=5,707)	FINRISK 1997 (N=7,909)	FINRISK 2002 (N=8,848)	MONICA-Friuli 1 R1 (N=1,852)	MONICA-Friuli 2 R1 (N=1,806)
Examination years, range	2002-2013 AV: 1959	1982-1982 AV: 8706	1987-1987 AV: 5471	1992-1992 AV: 5707	1997-1997 AV: 7909	2002-2002 AV: 8848	1985-1986 AV: 1852	1989-1989 AV: 1806
Examination age (years)	41·4 (28·4, 55·2) AV: 1959	43·9 (34·3, 54·2) AV: 8706	44·4 (34·5, 53·9) AV: 5471	44·4 (34·9, 54·0) AV: 5707	47·6 (36·5, 58·5) AV: 7909	46·7 (35·8, 56·8) AV: 8848	44·9 (36·4, 54·8) AV: 1852	44·2 (35·2, 53·1) AV: 1806
Age <45 years %	56·4 AV: 1959	52·2 AV: 8706	51·5 AV: 5471	52·2 AV: 5707	44·2 AV: 7909	46·3 AV: 8848	50·2 AV: 1852	52·3 AV: 1806
Age 45-59 years %	25·4 AV: 1959	38·0 AV: 8706	38·8 AV: 5471	37·4 AV: 5707	34·4 AV: 7909	35·1 AV: 8848	37·0 AV: 1852	37·9 AV: 1806
Age ≥60 years %	18·2 AV: 1959	9·8 AV: 8706	9·7 AV: 5471	10·3 AV: 5707	21·4 AV: 7909	18·6 AV: 8848	12·7 AV: 1852	9·8 AV: 1806
Female %	68·9 AV: 1959	51·2 AV: 8706	52·8 AV: 5471	53·7 AV: 5707	51·1 AV: 7909	54·4 AV: 8848	50·1 AV: 1852	50·3 AV: 1806
SCORE (%)	2·7 (0·7, 9·0) AV: 529	0·4 (0·1, 1·8) AV: 8615	0·4 (0·0, 1·6) AV: 5439	0·3 (0·0, 1·5) AV: 5697	0·5 (0·1, 2·4) AV: 7735	0·5 (0·1, 2·1) AV: 8055	0·5 (0·1, 2·0) AV: 1816	0·4 (0·1, 1·5) AV: 1779
PCE (%)	9·1 (1·1, 21·2) AV: 292	2·8 (0·8, 7·5) AV: 8278	2·4 (0·7, 6·4) AV: 5271	2·2 (0·6, 6·2) AV: 5681	2·7 (0·7, 8·9) AV: 7580	2·4 (0·6, 7·8) AV: 6308	3·3 (1·1, 8·2) AV: 1445	2·0 (0·6, 5·7) AV: 1441
BMI (kg/m²)	24·8 (22·1, 29·1) AV: 1957	25·5 (23·1, 28·4) AV: 8701	25·9 (23·3, 28·8) AV: 5468	25·6 (23·1, 28·8) AV: 5707	26·1 (23·5, 29·1) AV: 7906	26·1 (23·5, 29·3) AV: 8164	25·7 (23·2, 28·4) AV: 1822	25·2 (22·8, 28·2) AV: 1793
Hypertension %	27·9 AV: 1955	52·2 AV: 8706	50·1 AV: 5471	41·3 AV: 5707	44·1 AV: 7903	41·3 AV: 8237	41·9 AV: 1828	41·8 AV: 1804
Diabetes %	4·3 AV: 1939	3·4 AV: 8706	4·2 AV: 5471	3·6 AV: 5707	5·1 AV: 7909	4·6 AV: 8848	0·8 AV: 1477	0·9 AV: 1475
Daily smoker %	29·1 AV: 1951	26·7 AV: 8615	24·0 AV: 5439	25·3 AV: 5697	22·0 AV: 7773	25·1 AV: 8796	31·3 AV: 1849	29·2 AV: 1803
Cholesterol lowering medication %	4·9 AV: 1421	0 AV: 6986	0 AV: 4324	1·8 AV: 3831	3·2 AV: 5490	7·1 AV: 6465	AV: 0	AV: 0
LDL cholesterol (mmol/L)	3·5 (2·8, 4·1) AV: 276	AV: 0	AV: 0	3·4 (2·8, 4·2) AV: 5574	3·4 (2·8, 4·0) AV: 7725	3·4 (2·8, 4·1) AV: 7996	AV: 0	AV: 0
HDL cholesterol (mmol/L)	1·4 (1·1, 1·8) AV: 323	1·3 (1·1, 1·6) AV: 8706	1·4 (1·2, 1·7) AV: 5471	1·4 (1·1, 1·6) AV: 5707	1·4 (1·1, 1·6) AV: 7866	1·5 (1·2, 1·8) AV: 8095	1·3 (1·1, 1·6) AV: 1809	1·5 (1·2, 1·8) AV: 1781
Non-HDL cholesterol (mmol/L)	4·2 (3·3, 4·9) AV: 323	4·6 (3·8, 5·4) AV: 8706	4·4 (3·6, 5·3) AV: 5471	4·1 (3·3, 4·9) AV: 5707	4·0 (3·3, 4·8) AV: 7866	4·0 (3·3, 4·8) AV: 8095	4·6 (3·8, 5·5) AV: 1808	4·0 (3·2, 4·8) AV: 1776
Non-HDL cholesterol <2·6 mmol/L %	9·4 AV: 323	2·8 AV: 8706	4·2 AV: 5471	6·2 AV: 5707	6·6 AV: 7866	6·7 AV: 8095	2·9 AV: 1808	8·8 AV: 1776
Non-HDL cholesterol 2·6 to <3.7 mmol/L %	27·8 AV: 323	21·5 AV: 8706	26·2 AV: 5471	32·7 AV: 5707	32·5 AV: 7866	32·6 AV: 8095	20·3 AV: 1808	34·2 AV: 1776
Non-HDL cholesterol 3·7 to <4.8 mmol/L %	35·2 AV: 323	32·0 AV: 8706	32·0 AV: 5471	32·5 AV: 5707	35·0 AV: 7866	34·5 AV: 8095	31·5 AV: 1808	30·4 AV: 1776
Non-HDL cholesterol 4·8 to <5.7 mmol/L %	14·6 AV: 323	23·7 AV: 8706	20·5 AV: 5471	17·8 AV: 5707	17·2 AV: 7866	16·9 AV: 8095	24·2 AV: 1808	16·7 AV: 1776
Non-HDL cholesterol ≥5·7 mmol/L %	13·0 AV: 323	20·0 AV: 8706	17·0 AV: 5471	10·9 AV: 5707	8·7 AV: 7866	9·3 AV: 8095	21·1 AV: 1808	9·9 AV: 1776
Incident CVD %	2·6 AV: 1959	23·5 AV: 8706	16·9 AV: 5471	10·0 AV: 5707	8·9 AV: 7909	3·3 AV: 8848	2·9 AV: 1851	2·2 AV: 1803
Death %	4·3 AV: 1959	31·3 AV: 8706	19·4 AV: 5471	10·8 AV: 5707	10·3 AV: 7909	3·3 AV: 8848	6·2 AV: 1851	4·2 AV: 1803

	MONICA-Friuli 3 R1/ITA-FSE 21 R1 (N=2,071)	MATISS (N=10,127)	PAMELA 21 R1 (N=1,959)	Kaunas I (N=1,411)	Kaunas II (N=1,681)	Kaunas III (N=1,217)	MOLI-SANI (N=23,658)	MONICA Catalonia I and II (N=5,375)
Examination years, range	1994-1996 AV: 2071	1982-1996 AV: 10127	1990-1993 AV: 1959	1983-1985 AV: 1411	1986-1987 AV: 1681	1992-1993 AV: 1217	2005-2010 AV: 23658	1986-1992 AV: 5375
Examination age (years)	47·8 (37·6, 55·4) AV: 2071	46·4 (34·5, 57·0) AV: 10127	50·1 (39·8, 61·9) AV: 1959	50·7 (42·3, 57·1) AV: 1411	48·8 (42·2, 56·2) AV: 1681	49·5 (42·2, 56·9) AV: 1217	54·2 (45·6, 63·9) AV: 23658	45·3 (34·9, 55·2) AV: 5375
Age <45 years %	41·7 AV: 2071	47·0 AV: 10127	40·6 AV: 1959	34·9 AV: 1411	35·5 AV: 1681	35·5 AV: 1217	23·1 AV: 23658	49·4 AV: 5375
Age 45-59 years %	46·8 AV: 2071	35·0 AV: 10127	30·2 AV: 1959	50·6 AV: 1411	52·5 AV: 1681	49·1 AV: 1217	42·9 AV: 23658	37·8 AV: 5375
Age ≥60 years %	11·5 AV: 2071	18·1 AV: 10127	29·1 AV: 1959	14·5 AV: 1411	12·0 AV: 1681	15·4 AV: 1217	34·0 AV: 23658	12·8 AV: 5375
Female %	50·9 AV: 2071	52·9 AV: 10127	50·4 AV: 1959	51·5 AV: 1411	49·7 AV: 1681	52·0 AV: 1217	52·7 AV: 23658	45·8 AV: 5375
SCORE (%)	0·6 (0·1, 1·9) AV: 2068	0·4 (0·0, 2·2) AV: 9833	0·7 (0·1, 3·5) AV: 1940	1·5 (0·4, 4·2) AV: 1409	1·3 (0·4, 3·6) AV: 1613	1·4 (0·4, 3·9) AV: 1192	1·4 (0·4, 4·6) AV: 23246	0·3 (0·0, 1·4) AV: 5249
PCE (%)	2·7 (0·8, 6·9) AV: 2062	2·9 (0·8, 8·7) AV: 8624	4·0 (0·9, 12·2) AV: 1928	4·0 (1·4, 9·0) AV: 1240	3·7 (1·4, 8·2) AV: 1541	3·9 (1·5, 9·2) AV: 1136	5·3 (1·8, 14·9) AV: 22976	2·5 (0·8, 6·8) AV: 5245
BMI (kg/m²)	25·5 (22·8, 28·1) AV: 2070	26·8 (24·0, 30·0) AV: 10051	25·1 (22·6, 27·8) AV: 1913	28·3 (25·6, 31·4) AV: 1411	28·1 (25·2, 31·2) AV: 1681	27·0 (24·2, 30·3) AV: 1209	27·5 (24·7, 30·8) AV: 23646	26·0 (23·5, 28·6) AV: 5368
Hypertension %	40·8 AV: 2071	40·3 AV: 10103	40·2 AV: 1953	47·8 AV: 1411	43·7 AV: 1681	46·4 AV: 1217	55·3 AV: 23655	16·8 AV: 5371
Diabetes %	3·7 AV: 2067	4·5 AV: 8936	1·9 AV: 1953	2·3 AV: 1411	1·8 AV: 1681	1·9 AV: 1217	6·1 AV: 23500	3·7 AV: 5372
Daily smoker %	26·1 AV: 2071	30·2 AV: 9957	27·9 AV: 1955	20·8 AV: 1411	20·9 AV: 1681	18·3 AV: 1217	20·8 AV: 23396	34·9 AV: 5371
Cholesterol lowering medication %	1·1 AV: 2064	1·4 AV: 8265	AV: 0	AV: 0	AV: 0	0·3 AV: 1174	6·8 AV: 22498	1·3 AV: 2858
LDL cholesterol (mmol/L)	3·5 (2·9, 4·2) AV: 2024	3·5 (2·8, 4·1) AV: 8103	3·7 (3·1, 4·4) AV: 1931	3·9 (3·3, 4·6) AV: 1218	4·1 (3·4, 4·8) AV: 1528	4·2 (3·4, 5·0) AV: 1110	3·4 (2·8, 4·0) AV: 23166	3·6 (3·0, 4·3) AV: 5188
HDL cholesterol (mmol/L)	1·4 (1·2, 1·7) AV: 2067	1·3 (1·1, 1·5) AV: 10023	1·4 (1·1, 1·7) AV: 1944	1·4 (1·2, 1·6) AV: 1242	1·3 (1·0, 1·7) AV: 1541	1·2 (1·0, 1·5) AV: 1136	1·4 (1·2, 1·7) AV: 23509	1·2 (1·0, 1·5) AV: 5250
Non-HDL cholesterol (mmol/L)	4·1 (3·4, 4·9) AV: 2066	4·1 (3·4, 4·9) AV: 10021	4·3 (3·5, 5·1) AV: 1944	4·5 (3·9, 5·3) AV: 1241	4·7 (4·0, 5·5) AV: 1541	4·7 (3·9, 5·6) AV: 1136	4·1 (3·4, 4·8) AV: 23508	4·1 (3·4, 4·9) AV: 5249
Non-HDL cholesterol <2·6 mmol/L %	7·3 AV: 2066	6·4 AV: 10021	4·2 AV: 1944	2·8 AV: 1241	2·0 AV: 1541	2·5 AV: 1136	5·6 AV: 23508	5·8 AV: 5249
Non-HDL cholesterol 2·6 to <3.7 mmol/L %	31·5 AV: 2066	30·6 AV: 10021	27·5 AV: 1944	18·9 AV: 1241	17·3 AV: 1541	17·7 AV: 1136	32·6 AV: 23508	30·3 AV: 5249
Non-HDL cholesterol 3·7 to <4.8 mmol/L %	32·6 AV: 2066	34·8 AV: 10021	34·3 AV: 1944	38·2 AV: 1241	32·6 AV: 1541	32·2 AV: 1136	36·7 AV: 23508	34·9 AV: 5249
Non-HDL cholesterol 4·8 to <5.7 mmol/L %	18·0 AV: 2066	17·4 AV: 10021	22·3 AV: 1944	23·6 AV: 1241	28·1 AV: 1541	25·4 AV: 1136	16·7 AV: 23508	18·7 AV: 5249
Non-HDL cholesterol ≥5·7 mmol/L %	10·6 AV: 2066	10·7 AV: 10021	11·8 AV: 1944	16·5 AV: 1241	20·1 AV: 1541	22·2 AV: 1136	8·4 AV: 23508	10·4 AV: 5249
Incident CVD %	1·1 AV: 2069	5·5 AV: 10114	4·5 AV: 1959	12·8 AV: 1411	10·9 AV: 1681	8·1 AV: 1217	1·7 AV: 23214	1·6 AV: 5375
Death %	1·6 AV: 2069	15·1 AV: 10114	7·9 AV: 1959	42·2 AV: 1411	33·0 AV: 1681	21·9 AV: 1217	2·1 AV: 23214	3·6 AV: 5375

	MONICA/KORA S3 (N=4,319)	MONICA/KORA S4 (N=4,032)	MONICA Northern Sweden 1986 (N=1,547)	MONICA Northern Sweden 1990 (N=1,537)	MONICA Northern Sweden 1994 (N=1,797)	MONICA Northern Sweden 1999 (N=1,682)	MONICA Northern Sweden 2004/2009 (N=3,350)	Warsaw 1 R1 (N=2,239)
Examination years, range	1994-1995 AV: 4319	1999-2001 AV: 4032	1986-1986 AV: 1547	1990-1990 AV: 1537	1994-1994 AV: 1797	1999-1999 AV: 1682	2004-2009 AV: 3350	1983-1985 AV: 2239
Examination age (years)	50·1 (37·4, 61·1) AV: 4319	48·9 (36·9, 60·9) AV: 4032	45·3 (36·1, 54·4) AV: 1547	44·7 (35·5, 54·3) AV: 1537	48·6 (36·7, 60·3) AV: 1797	49·7 (37·6, 61·4) AV: 1682	49·6 (37·8, 61·6) AV: 3350	49·8 (42·4, 57·1) AV: 2239
Age <45 years %	40·3 AV: 4319	41·8 AV: 4032	49·1 AV: 1547	50·8 AV: 1537	41·5 AV: 1797	40·2 AV: 1682	39·8 AV: 3350	33·5 AV: 2239
Age 45-59 years %	32·7 AV: 4319	31·1 AV: 4032	39·8 AV: 1547	38·9 AV: 1537	32·6 AV: 1797	32·2 AV: 1682	31·8 AV: 3350	52·3 AV: 2239
Age ≥60 years %	27·0 AV: 4319	27·0 AV: 4032	11·1 AV: 1547	10·3 AV: 1537	25·9 AV: 1797	27·6 AV: 1682	28·4 AV: 3350	14·1 AV: 2239
Female %	49·9 AV: 4319	52·0 AV: 4032	50·0 AV: 1547	51·5 AV: 1537	52·2 AV: 1797	52·1 AV: 1682	52·2 AV: 3350	50·3 AV: 2239
SCORE (%)	0·8 (0·1, 3·5) AV: 4319	0·6 (0·1, 2·8) AV: 4014	0·3 (0·1, 1·5) AV: 1540	0·3 (0·0, 1·5) AV: 1526	0·6 (0·1, 3·2) AV: 1785	0·7 (0·1, 3·5) AV: 1672	0·6 (0·1, 2·9) AV: 3298	1·7 (0·5, 4·8) AV: 2198
PCE (%)	4·2 (1·0, 12·4) AV: 4314	3·2 (0·8, 10·1) AV: 4005	3·0 (0·9, 7·4) AV: 1531	2·5 (0·7, 6·8) AV: 1516	3·5 (0·8, 10·8) AV: 1763	3·1 (0·7, 11·1) AV: 1609	3·3 (0·8, 10·8) AV: 3130	4·6 (1·9, 9·6) AV: 2187
BMI (kg/m^2)	26·4 (23·8, 29·3) AV: 4289	26·5 (23·8, 29·7) AV: 4001	24·7 (22·5, 27·4) AV: 1542	24·8 (22·7, 27·5) AV: 1533	25·3 (23·0, 28·2) AV: 1788	25·9 (23·6, 28·6) AV: 1679	28·7 (25·4, 32·4) AV: 3324	26·5 (24·0, 29·3) AV: 2239
Hypertension %	41·6 AV: 4319	36·1 AV: 4016	30·1 AV: 1547	29·1 AV: 1537	35·0 AV: 1796	38·5 AV: 1682	34·1 AV: 3339	52·4 AV: 2239
Diabetes %	4·4 AV: 4319	4·6 AV: 4032	2·6 AV: 1547	2·6 AV: 1537	3·0 AV: 1797	2·9 AV: 1682	3·7 AV: 3350	2·9 AV: 2239
Daily smoker %	23·0 AV: 4319	23·1 AV: 4028	24·8 AV: 1540	24·7 AV: 1537	21·4 AV: 1793	15·9 AV: 1680	12·5 AV: 3322	45·9 AV: 2239
Cholesterol lowering medication %	AV: 0	0 AV: 2686	AV: 0	0·5 AV: 1525	0·8 AV: 1783	3·0 AV: 1668	6·7 AV: 3336	AV: 0
LDL cholesterol (mmol/L)	3·6 (3·0, 4·3) AV: 4082	4·0 (3·4, 4·7) AV: 1570	4·0 (3·3, 4·8) AV: 1019	4·0 (3·3, 4·9) AV: 891	4·0 (3·3, 4·8) AV: 1312	3·6 (2·8, 4·6) AV: 1623	3·1 (2·4, 3·9) AV: 3228	3·3 (2·8, 3·9) AV: 2151
HDL cholesterol (mmol/L)	1·3 (1·1, 1·6) AV: 4314	1·4 (1·2, 1·8) AV: 4024	1·2 (1·0, 1·4) AV: 1545	1·4 (1·2, 1·6) AV: 1527	1·3 (1·1, 1·6) AV: 1790	1·5 (1·2, 1·8) AV: 1623	1·2 (1·0, 1·5) AV: 3228	1·4 (1·2, 1·6) AV: 2189
Non-HDL cholesterol (mmol/L)	4·5 (3·7, 5·3) AV: 4314	4·3 (3·6, 5·1) AV: 4024	4·7 (3·9, 5·7) AV: 1545	4·7 (3·8, 5·7) AV: 1522	4·7 (3·8, 5·6) AV: 1790	4·3 (3·4, 5·2) AV: 1622	4·5 (3·6, 5·3) AV: 3222	4·0 (3·4, 4·7) AV: 2187
Non-HDL cholesterol <2·6 mmol/L %	3·2 AV: 4314	5·3 AV: 4024	1·6 AV: 1545	2·6 AV: 1522	3·2 AV: 1790	7·2 AV: 1622	4·4 AV: 3222	4·6 AV: 2187
Non-HDL cholesterol 2·6 to <3.7 mmol/L %	22·1 AV: 4314	25·4 AV: 4024	19·8 AV: 1545	21·0 AV: 1522	19·4 AV: 1790	26·8 AV: 1622	24·4 AV: 3222	33·2 AV: 2187
Non-HDL cholesterol 3·7 to <4.8 mmol/L %	35·2 AV: 4314	35·4 AV: 4024	30·9 AV: 1545	29·0 AV: 1522	32·5 AV: 1790	30·3 AV: 1622	31·7 AV: 3222	38·4 AV: 2187
Non-HDL cholesterol 4·8 to <5.7 mmol/L %	22·8 AV: 4314	20·4 AV: 4024	22·2 AV: 1545	22·0 AV: 1522	21·7 AV: 1790	21·5 AV: 1622	22·1 AV: 3222	16·8 AV: 2187
Non-HDL cholesterol ≥5·7 mmol/L %	16·7 AV: 4314	13·6 AV: 4024	25·5 AV: 1545	25·3 AV: 1522	23·2 AV: 1790	14·2 AV: 1622	17·3 AV: 3222	7·1 AV: 2187
Incident CVD %	6·6 AV: 4017	3·9 AV: 3618	20·2 AV: 1547	15·9 AV: 1537	15·6 AV: 1797	9·6 AV: 1682	3·4 AV: 3350	5·1 AV: 2190
Death %	12·4 AV: 4319	5·3 AV: 4032	23·3 AV: 1547	14·2 AV: 1537	16·6 AV: 1797	8·8 AV: 1682	2·2 AV: 3350	18·3 AV: 2226

	Warsaw 2 R1/POL-WAR 3 R1 (N=2,550)	PRIME/Belfast (N=2,560)	PRIME/Lille (N=2,554)	PRIME/Strasbourg (N=2,544)	PRIME/Toulouse (N=2,574)	Novosibirsk 1 R1 (N=3,065)	Novosibirsk 2 R1/ Novosibirsk 3 R1 (N=6,357)	Novosibirsk 21 R1 (N=1,546)
Examination years, range	1988-1993 AV: 2550	1991-1994 AV: 2560	1991-1993 AV: 2554	1991-1993 AV: 2544	1991-1993 AV: 2574	1985-1986 AV: 3065	1988-1995 AV: 6357	1983-1985 AV: 1546
Examination age (years)	48·6 (41·3, 55·8) AV: 2550	54·6 (52·2, 57·3) AV: 2560	55·2 (52·6, 57·6) AV: 2554	54·6 (52·2, 57·2) AV: 2544	55·1 (52·5, 57·2) AV: 2574	45·3 (35·9, 55·2) AV: 3065	44·7 (34·8, 54·6) AV: 6357	46·6 (42·1, 51·0) AV: 1546
Age <45 years %	37·6 AV: 2550	0 AV: 2560	0 AV: 2554	0 AV: 2544	0 AV: 2574	49·0 AV: 3065	50·4 AV: 6357	38·8 AV: 1546
Age 45-59 years %	50·9 AV: 2550	99·2 AV: 2560	97·7 AV: 2554	99·5 AV: 2544	99·5 AV: 2574	40·8 AV: 3065	38·3 AV: 6357	61·0 AV: 1546
Age ≥60 years %	11·5 AV: 2550	0·8 AV: 2560	2·3 AV: 2554	0·5 AV: 2544	0·5 AV: 2574	10·2 AV: 3065	11·4 AV: 6357	0·2 AV: 1546
Female %	49·6 AV: 2550	0 AV: 2560	0 AV: 2554	0 AV: 2544	0 AV: 2574	50·5 AV: 3065	50·3 AV: 6357	0 AV: 1546
SCORE (%)	1·2 (0·4, 3·5) AV: 2540	2·0 (1·4, 3·1) AV: 2548	2·3 (1·5, 3·4) AV: 2532	2·0 (1·4, 3·0) AV: 2485	1·7 (1·2, 2·3) AV: 2559	0·6 (0·1, 2·8) AV: 2725	0·6 (0·1, 2·7) AV: 6013	2·1 (1·0, 4·1) AV: 1426
PCE (%)	3·5 (1·5, 7·6) AV: 2521	8·7 (5·9, 12·3) AV: 2547	8·3 (5·6, 12·0) AV: 2336	8·0 (5·6, 11·7) AV: 2344	7·1 (4·9, 10·0) AV: 2423	AV: 0	2·1 (0·6, 6·4) AV: 5487	AV: 0
BMI (kg/m²)	26·7 (23·9, 29·7) AV: 2550	25·9 (23·9, 28·0) AV: 2560	26·3 (24·1, 28·5) AV: 2554	27·0 (24·9, 29·3) AV: 2543	26·1 (24·2, 28·0) AV: 2574	26·7 (23·9, 30·1) AV: 3065	26·2 (23·4, 29·9) AV: 6241	25·6 (23·5, 28·0) AV: 1546
Hypertension %	38·5 AV: 2550	38·7 AV: 2558	53·6 AV: 2552	49·7 AV: 2538	27·3 AV: 2571	43·8 AV: 3065	42·2 AV: 6353	50·3 AV: 1546
Diabetes %	2·7 AV: 2550	2·1 AV: 2560	6·2 AV: 2357	4·6 AV: 2403	4·7 AV: 2438	AV: 0	6·1 AV: 5895	AV: 0
Daily smoker %	44·2 AV: 2550	23·3 AV: 2559	19·3 AV: 2536	18·3 AV: 2524	18·9 AV: 2564	31·0 AV: 3065	32·4 AV: 6335	57·9 AV: 1544
Cholesterol lowering medication %	1·3 AV: 815	1·1 AV: 2560	12·7 AV: 2554	10·0 AV: 2544	13·6 AV: 2574	AV: 0	0·1 AV: 6124	AV: 0
LDL cholesterol (mmol/L)	3·5 (2·9, 4·2) AV: 2506	3·8 (3·2, 4·4) AV: 2466	3·8 (3·2, 4·5) AV: 2507	3·9 (3·2, 4·5) AV: 2430	3·7 (3·2, 4·3) AV: 2540	3·6 (3·0, 4·2) AV: 2337	3·4 (2·7, 4·1) AV: 5914	3·8 (3·1, 4·4) AV: 1363
HDL cholesterol (mmol/L)	1·4 (1·2, 1·7) AV: 2521	1·2 (1·0, 1·4) AV: 2552	1·3 (1·1, 1·6) AV: 2553	1·3 (1·0, 1·4) AV: 2507	1·3 (1·0, 1·4) AV: 2571	1·2 (1·1, 1·5) AV: 2484	1·4 (1·2, 1·7) AV: 5960	1·2 (1·0, 1·5) AV: 1395
Non-HDL cholesterol (mmol/L)	4·1 (3·5, 4·9) AV: 2521	4·7 (4·0, 5·3) AV: 2551	4·5 (3·8, 5·3) AV: 2553	4·6 (3·9, 5·4) AV: 2507	4·4 (3·7, 5·2) AV: 2571	4·2 (3·5, 4·9) AV: 2483	3·9 (3·1, 4·7) AV: 5960	4·3 (3·6, 5·1) AV: 1389
Non-HDL cholesterol <2·6 mmol/L %	4·2 AV: 2521	1·2 AV: 2551	2·3 AV: 2553	2·0 AV: 2507	2·3 AV: 2571	4·3 AV: 2483	11·6 AV: 5960	3·6 AV: 1389
Non-HDL cholesterol 2·6 to <3.7 mmol/L %	31·3 AV: 2521	16·4 AV: 2551	20·6 AV: 2553	18·1 AV: 2507	23·5 AV: 2571	31·1 AV: 2483	33·9 AV: 5960	25·2 AV: 1389
Non-HDL cholesterol 3·7 to <4.8 mmol/L %	36·8 AV: 2521	37·9 AV: 2551	38·0 AV: 2553	36·7 AV: 2507	38·5 AV: 2571	37·3 AV: 2483	31·6 AV: 5960	36·5 AV: 1389
Non-HDL cholesterol 4·8 to <5.7 mmol/L %	19·2 AV: 2521	27·9 AV: 2551	22·8 AV: 2553	24·3 AV: 2507	21·7 AV: 2571	17·7 AV: 2483	14·1 AV: 5960	19·9 AV: 1389
Non-HDL cholesterol ≥5·7 mmol/L %	8·5 AV: 2521	16·6 AV: 2551	16·4 AV: 2553	19·0 AV: 2507	14·1 AV: 2571	9·6 AV: 2483	8·8 AV: 5960	14·8 AV: 1389
Incident CVD %	1·6 AV: 2546	17·0 AV: 2559	7·1 AV: 2554	6·8 AV: 2539	6·6 AV: 2572	4·2 AV: 3039	1·9 AV: 6342	7·1 AV: 1546
Death %	6·0 AV: 2546	18·6 AV: 2559	6·1 AV: 2554	5·6 AV: 2539	3·7 AV: 2572	13·2 AV: 3065	6·0 AV: 6357	20·1 AV: 1546

	SHHEC 1 (N=11,166)	SHHEC 2 (N=1,633)	SHHEC 21 (N=976)	SHHEC 3 (N=1,565)	Tromso 1986-1987 (N=4,851)	Tromso 1994-1995 (N=25,705)
Examination years, range	1984-1987 AV: 11166	1992-1992 AV: 1633	1988-1989 AV: 976	1995-1995 AV: 1565	1986-1987 AV: 4851	1994-1995 AV: 25705
Examination age (years)	49·8 (44·0, 55·1) AV: 11166	51·7 (38·5, 63·7) AV: 1633	46·8 (37·6, 56·1) AV: 976	45·1 (35·4, 54·6) AV: 1565	32·8 (26·4, 40·8) AV: 4851	44·4 (34·9, 55·8) AV: 25705
Age <45 years %	29·0 AV: 11166	36·7 AV: 1633	45·2 AV: 976	49·5 AV: 1565	83·7 AV: 4851	51·7 AV: 25705
Age 45-59 years %	66·4 AV: 11166	30·5 AV: 1633	40·3 AV: 976	37·1 AV: 1565	14·7 AV: 4851	28·8 AV: 25705
Age ≥60 years %	4·6 AV: 11166	32·8 AV: 1633	14·5 AV: 976	13·4 AV: 1565	1·6 AV: 4851	19·4 AV: 25705
Female %	49·6 AV: 11166	53·0 AV: 1633	52·0 AV: 976	53·0 AV: 1565	46·8 AV: 4851	53·4 AV: 25705
SCORE (%)	0·9 (0·3, 2·1) AV: 11162	1·1 (0·1, 4·8) AV: 1633	0·5 (0·1, 2·3) AV: 976	0·4 (0·1, 1·7) AV: 1565	0·0 (0·0, 0·3) AV: 4824	0·4 (0·1, 2·4) AV: 25628
PCE (%)	3·9 (1·7, 8·1) AV: 10690	5·2 (1·5, 14·3) AV: 1551	4·4 (1·5, 10·0) AV: 766	3·2 (0·9, 7·7) AV: 1507	1·1 (0·3, 3·6) AV: 3573	2·9 (0·8, 9·3) AV: 25501
BMI (kg/m²)	25·3 (23·0, 27·8) AV: 11141	25·4 (22·9, 28·5) AV: 1629	25·1 (22·9, 27·8) AV: 975	25·7 (23·0, 28·7) AV: 1561	23·1 (21·2, 25·5) AV: 4839	24·6 (22·5, 27·2) AV: 25663
Hypertension %	37·2 AV: 11166	38·8 AV: 1633	31·8 AV: 976	30·2 AV: 1565	24·4 AV: 4847	36·6 AV: 25700
Diabetes %	1·4 AV: 11166	1·7 AV: 1633	2·3 AV: 976	2·1 AV: 1565	0·9 AV: 4850	1·6 AV: 25682
Daily smoker %	37·2 AV: 11162	42·0 AV: 1633	43·1 AV: 976	38·8 AV: 1565	50·3 AV: 4851	36·5 AV: 25677
Cholesterol lowering medication %	AV: 0	0·3 AV: 1623	AV: 0	0·4 AV: 1563	AV: 0	0·7 AV: 20019
LDL cholesterol (mmol/L)	4·0 (3·3, 4·7) AV: 10312	3·6 (3·0, 4·3) AV: 1508	3·8 (3·1, 4·6) AV: 745	3·6 (3·0, 4·3) AV: 1453	AV: 0	3·7 (3·0, 4·6) AV: 25106
HDL cholesterol (mmol/L)	1·5 (1·2, 1·8) AV: 10708	1·4 (1·2, 1·7) AV: 1554	1·1 (1·0, 1·4) AV: 767	1·3 (1·1, 1·6) AV: 1507	1·4 (1·2, 1·7) AV: 4839	1·5 (1·2, 1·8) AV: 25625
Non-HDL cholesterol (mmol/L)	4·8 (4·0, 5·6) AV: 10708	4·4 (3·7, 5·2) AV: 1554	4·5 (3·8, 5·4) AV: 767	4·5 (3·7, 5·3) AV: 1507	3·8 (3·1, 4·8) AV: 4838	4·4 (3·6, 5·4) AV: 25612
Non-HDL cholesterol <2·6 mmol/L %	1·3 AV: 10708	1·7 AV: 1554	2·0 AV: 767	3·5 AV: 1507	10·2 AV: 4838	5·1 AV: 25612
Non-HDL cholesterol 2·6 to <3.7 mmol/L %	16·7 AV: 10708	25·8 AV: 1554	21·9 AV: 767	22·1 AV: 1507	37·4 AV: 4838	25·2 AV: 25612
Non-HDL cholesterol 3·7 to <4.8 mmol/L %	32·0 AV: 10708	35·0 AV: 1554	33·8 AV: 767	32·8 AV: 1507	27·9 AV: 4838	30·5 AV: 25612
Non-HDL cholesterol 4·8 to <5.7 mmol/L %	26·0 AV: 10708	21·4 AV: 1554	23·6 AV: 767	24·8 AV: 1507	14·1 AV: 4838	20·3 AV: 25612
Non-HDL cholesterol ≥5·7 mmol/L %	24·0 AV: 10708	16·0 AV: 1554	18·8 AV: 767	16·9 AV: 1507	10·4 AV: 4838	18·9 AV: 25612
Incident CVD %	18·2 AV: 11166	16·9 AV: 1633	14·0 AV: 976	8·2 AV: 1565	6·5 AV: 4851	10·7 AV: 25703
Death %	26·6 AV: 11166	30·7 AV: 1633	23·5 AV: 976	12·6 AV: 1565	15·7 AV: 4851	13·2 AV: 25705

Supplement table S1. Weighted baseline characteristics of the MORGAM/BiomarCaRE dataset. For continuous variables median values (IQR) and for categorical variables percentages are shown. Number of individuals with available information is given for each variable (AV). SCORE = Systematic Coronary Risk Estimation; PCE = Pooled Cohort Equations; BMI = body-mass-index; non-HDL- / LDL-cholesterol = non-high density / low density lipoprotein-related cholesterol.

Table S2: Baseline information – non-MORGAM/non-BiomarCaRE cohorts

	ARIC (N=14,029)	ATTICA (N=3,042)	AusDiab (N=10,604)	BRHS (N=3,798)	CHS (N=4,504)	DETECT (N=6,224)	DHS (N=3,447)
Examination years, range	1986-1990 AV: 14029	2001-2003 AV: 2417	1999-2000 AV: 10604	1998-2000 AV: 3798	AV: 0	2003-2003 AV: 6224	2000-2002 AV: 3447
Examination age (years)	54·0 (49·0, 59·0) AV: 14029	45·0 (35·0, 54·0) AV: 3042	49·0 (40·0, 60·0) AV: 10604	68·1 (64·0, 72·9) AV: 3798	71·0 (67·0, 75·0) AV: 4504	59·0 (46·0, 68·0) AV: 6224	43·0 (36·0, 51·0) AV: 3447
Age <45 years %	0·3 AV: 14029	49·2 AV: 3042	37·0 AV: 10604	0 AV: 3798	0 AV: 4504	21·8 AV: 6224	54·6 AV: 3447
Age 45-59 years %	77·5 AV: 14029	35·6 AV: 3042	36·5 AV: 10604	1·9 AV: 3798	0 AV: 4504	29·8 AV: 6224	38·7 AV: 3447
Age ≥60 years %	22·2 AV: 14029	15·1 AV: 3042	26·5 AV: 10604	98·1 AV: 3798	100·0 AV: 4504	48·4 AV: 6224	6·7 AV: 3447
Female %	56·3 AV: 14029	50·2 AV: 3042	56·0 AV: 10604	0 AV: 3798	60·7 AV: 4504	61·3 AV: 6224	56·1 AV: 3447
SCORE (%)	0·9 (0·4, 1·8) AV: 13797	0·3 (0·0, 1·3) AV: 2632	0·6 (0·1, 2·5) AV: 10515	8·6 (5·3, 13·8) AV: 3597	6·1 (3·8, 9·9) AV: 4383	1·9 (0·3, 5·4) AV: 5819	0·2 (0·0, 0·7) AV: 3414
PCE (%)	4·9 (2·2, 9·6) AV: 13761	7·2 (2·7, 15·3) AV: 861	2·9 (0·8, 9·6) AV: 10486	24·5 (17·1, 33·7) AV: 3525	20·4 (12·7, 31·5) AV: 4351	8·4 (2·4, 21·6) AV: 5563	2·1 (0·6, 5·8) AV: 3339
BMI (kg/m²)	26·8 (24·0, 30·4) AV: 14005	25·9 (23·2, 29·0) AV: 2994	26·2 (23·5, 29·5) AV: 10437	26·6 (24·5, 29·0) AV: 3783	26·1 (23·5, 29·2) AV: 4490	26·4 (23·7, 29·8) AV: 6205	28·4 (24·5, 33·2) AV: 3419
Hypertension %	36·7 AV: 14016	66·1 AV: 1001	29·7 AV: 10516	74·2 AV: 3772	62·3 AV: 4464	53·4 AV: 5893	30·8 AV: 3361
Diabetes %	11·2 AV: 13902	6·9 AV: 3042	7·4 AV: 10604	5·8 AV: 3798	14·3 AV: 4460	14·6 AV: 6224	11·1 AV: 3440
Daily smoker %	26·1 AV: 14014	43·4 AV: 3035	14·7 AV: 10536	13·1 AV: 3792	10·2 AV: 4453	20·9 AV: 6009	28·6 AV: 3442
Cholesterol lowering medication %	2·5 AV: 13920	5·7 AV: 2093	6·9 AV: 10391	5·2 AV: 3798	4·3 AV: 4492	15·4 AV: 5926	5·3 AV: 3355
LDL cholesterol (mmol/L)	3·5 (2·9, 4·2) AV: 13628	3·1 (2·5, 3·8) AV: 2310	3·6 (3·0, 4·2) AV: 10305	3·9 (3·3, 4·6) AV: 3596	3·3 (2·8, 4·0) AV: 4415	4·1 (3·4, 4·9) AV: 5930	AV: 0
HDL cholesterol (mmol/L)	1·3 (1·0, 1·6) AV: 13820	1·2 (1·0, 1·4) AV: 2679	1·4 (1·1, 1·7) AV: 10601	1·3 (1·1, 1·5) AV: 3596	1·4 (1·1, 1·7) AV: 4461	1·4 (1·1, 1·7) AV: 6117	1·2 (1·0, 1·5) AV: 3439
Non-HDL cholesterol (mmol/L)	4·1 (3·4, 4·9) AV: 13817	3·7 (3·0, 4·5) AV: 2674	4·3 (3·5, 5·1) AV: 10600	4·7 (4·0, 5·5) AV: 3596	4·0 (3·4, 4·7) AV: 4461	4·5 (3·7, 5·4) AV: 6117	3·3 (2·7, 4·1) AV: 3439
Non-HDL cholesterol <2·6 mmol/L %	6·0 AV: 13817	13·4 AV: 2674	4·5 AV: 10600	1·2 AV: 3596	6·2 AV: 4461	3·2 AV: 6117	21·1 AV: 3439
Non-HDL cholesterol 2·6 to <3.7 mmol/L %	29·8 AV: 13817	38·7 AV: 2674	27·4 AV: 10600	16·3 AV: 3596	32·7 AV: 4461	22·2 AV: 6117	44·1 AV: 3439
Non-HDL cholesterol 3·7 to <4.8 mmol/L %	34·8 AV: 13817	29·1 AV: 2674	36·0 AV: 10600	34·9 AV: 3596	37·7 AV: 4461	34·5 AV: 6117	23·6 AV: 3439
Non-HDL cholesterol 4·8 to <5.7 mmol/L %	18·4 AV: 13817	12·4 AV: 2674	19·9 AV: 10600	27·3 AV: 3596	15·2 AV: 4461	21·2 AV: 6117	7·5 AV: 3439
Non-HDL cholesterol ≥5·7 mmol/L %	11·0 AV: 13817	6·4 AV: 2674	12·2 AV: 10600	20·3 AV: 3596	8·2 AV: 4461	19·0 AV: 6117	3·8 AV: 3439
Incident CVD %	20·4 AV: 14029	15·7 AV: 2020	3·2 AV: 8774	19·2 AV: 3797	49·3 AV: 4503	7·2 AV: 6224	4·5 AV: 2959
Death %	40·1 AV: 14029	3·7 AV: 2583	12·1 AV: 10604	37·6 AV: 3797	78·6 AV: 4503	3·4 AV: 6224	5·0 AV: 2960

	Dubbo (N=2,102)	FHS Gen3/OMNI 2/NOS (N=4,507)	FHS offspring (N=4,792)	Health 2000 (N=7,384)	HNRS (N=4,471)	HUNT (N=9,557)	JHS (N=2,413)
Examination years, range	1988-1989 AV: 2102	AV: 0	AV: 0	2000-2001 AV: 7384	2000-2003 AV: 4471	1996-1997 AV: 9555	2000-2004 AV: 2413
Examination age (years)	66·9 (62·9, 73·0) AV: 2102	40·0 (34·0, 47·0) AV: 4507	36·0 (29·0, 44·0) AV: 4792	50·9 (40·9, 63·6) AV: 7384	60·0 (53·0, 65·0) AV: 4471	47·9 (36·2, 61·8) AV: 9555	48·0 (41·2, 55·0) AV: 2413
Age <45 years %	0 AV: 2102	65·9 AV: 4507	75·8 AV: 4792	34·9 AV: 7384	0 AV: 4471	43·8 AV: 9555	36·3 AV: 2413
Age 45-59 years %	4·8 AV: 2102	31·3 AV: 4507	23·4 AV: 4792	34·3 AV: 7384	50·0 AV: 4471	28·6 AV: 9555	48·4 AV: 2413
Age ≥60 years %	95·2 AV: 2102	2·8 AV: 4507	0·8 AV: 4792	30·7 AV: 7384	50·0 AV: 4471	27·6 AV: 9555	15·3 AV: 2413
Female %	58·2 AV: 2102	54·0 AV: 4507	52·2 AV: 4792	55·4 AV: 7384	52·0 AV: 4471	55·0 AV: 9557	62·5 AV: 2413
SCORE (%)	6·4 (3·5, 12·1) AV: 2072	0·1 (0·0, 0·4) AV: 4492	0·1 (0·0, 0·3) AV: 4594	0·9 (0·2, 4·0) AV: 6073	2·1 (0·9, 4·5) AV: 4437	0·6 (0·1, 3·9) AV: 9093	0·3 (0·1, 1·1) AV: 2204
PCE (%)	18·7 (10·8, 30·4) AV: 2060	0·8 (0·3, 2·2) AV: 4473	1·3 (0·4, 3·6) AV: 4515	4·6 (1·4, 13·8) AV: 5609	8·1 (3·6, 15·9) AV: 4431	3·8 (0·9, 12·6) AV: 9074	4·0 (1·3, 9·2) AV: 2181
BMI (kg/m²)	25·5 (23·1, 28·4) AV: 2098	25·9 (22·9, 29·6) AV: 4502	24·6 (22·1, 27·6) AV: 4791	26·1 (23·5, 29·4) AV: 6605	27·3 (24·7, 30·2) AV: 4447	25·8 (23·5, 28·5) AV: 9517	30·8 (26·9, 35·9) AV: 2408
Hypertension %	68·7 AV: 2102	16·5 AV: 4496	15·3 AV: 4762	42·2 AV: 5863	51·9 AV: 4462	40·0 AV: 9512	49·4 AV: 2389
Diabetes %	5·7 AV: 2086	3·3 AV: 4496	1·8 AV: 4573	5·0 AV: 6735	12·5 AV: 4471	2·6 AV: 9540	17·1 AV: 2387
Daily smoker %	15·6 AV: 2079	14·0 AV: 4504	45·3 AV: 4774	23·3 AV: 6442	20·0 AV: 4462	29·0 AV: 9145	13·9 AV: 2395
Cholesterol lowering medication %	AV: 0	7·5 AV: 4505	0·4 AV: 4768	4·6 AV: 6109	10·6 AV: 4177	AV: 0	8·1 AV: 2383
LDL cholesterol (mmol/L)	4·3 (3·6, 5·0) AV: 2086	2·9 (2·3, 3·5) AV: 4500	3·2 (2·7, 3·9) AV: 4694	3·9 (3·2, 4·6) AV: 5950	3·8 (3·2, 4·4) AV: 4438	3·6 (2·9, 4·4) AV: 9547	3·3 (2·6, 3·9) AV: 2199
HDL cholesterol (mmol/L)	1·4 (1·1, 1·6) AV: 2092	1·3 (1·1, 1·7) AV: 4501	1·3 (1·0, 1·5) AV: 4699	1·3 (1·1, 1·6) AV: 6128	1·4 (1·2, 1·8) AV: 4448	1·4 (1·1, 1·6) AV: 9552	1·3 (1·1, 1·5) AV: 2224
Non-HDL cholesterol (mmol/L)	5·0 (4·3, 5·9) AV: 2092	3·5 (2·8, 4·2) AV: 4501	3·6 (3·0, 4·4) AV: 4699	4·6 (3·8, 5·4) AV: 6128	4·5 (3·9, 5·3) AV: 4447	4·4 (3·5, 5·3) AV: 9552	3·8 (3·1, 4·5) AV: 2224
Non-HDL cholesterol <2·6 mmol/L %	0·7 AV: 2092	16·7 AV: 4501	11·4 AV: 4699	2·2 AV: 6128	2·0 AV: 4447	4·7 AV: 9552	9·8 AV: 2224
Non-HDL cholesterol 2·6 to <3.7 mmol/L %	9·9 AV: 2092	42·9 AV: 4501	41·8 AV: 4699	20·8 AV: 6128	19·5 AV: 4447	26·3 AV: 9552	37·9 AV: 2224
Non-HDL cholesterol 3·7 to <4.8 mmol/L %	31·4 AV: 2092	27·5 AV: 4501	30·0 AV: 4699	33·4 AV: 6128	37·2 AV: 4447	30·5 AV: 9552	32·8 AV: 2224
Non-HDL cholesterol 4·8 to <5.7 mmol/L %	27·8 AV: 2092	9·1 AV: 4501	11·7 AV: 4699	24·3 AV: 6128	25·3 AV: 4447	22·0 AV: 9552	12·8 AV: 2224
Non-HDL cholesterol ≥5·7 mmol/L %	30·2 AV: 2092	3·9 AV: 4501	5·2 AV: 4699	19·2 AV: 6128	15·9 AV: 4447	16·6 AV: 9552	6·6 AV: 2224
Incident CVD %	37·2 AV: 2102	1·0 AV: 4401	21·5 AV: 4758	13·4 AV: 6187	11·1 AV: 4452	10·3 AV: 9555	4·6 AV: 2371
Death %	43·2 AV: 2102	0·8 AV: 4468	28·3 AV: 4790	19·8 AV: 6187	13·9 AV: 4471	22·5 AV: 9557	8·3 AV: 2371

	MDCS (N=29,492)	MESA (N=6,814)	MPP (N=33,183)	PREVEND (N=8,137)	Rotterdam (N=10,057)	ULSAM (N=2,310)
Examination years, range	1991-1996 AV: 29486	AV: 0	1974-1992 AV: 33183	1997-1998 AV: 8137	1997-2008 AV: 10057	1970-1973 AV: 2310
Examination age (years)	57·4 (51·1, 63·9) AV: 29486	62·0 (53·0, 70·0) AV: 6814	46·9 (39·8, 49·1) AV: 33183	47·0 (38·0, 58·0) AV: 8137	63·0 (58·0, 72·0) AV: 10057	49·6 (49·2, 50·1) AV: 2310
Age <45 years %	0·2 AV: 29486	0·1 AV: 6814	40·1 AV: 33183	42·7 AV: 8137	0 AV: 10057	0 AV: 2310
Age 45-59 years %	59·3 AV: 29486	42·7 AV: 6814	58·1 AV: 33183	34·3 AV: 8137	33·9 AV: 10057	100·0 AV: 2310
Age ≥60 years %	40·5 AV: 29486	57·2 AV: 6814	1·8 AV: 33183	23·0 AV: 8137	66·1 AV: 10057	0 AV: 2310
Female %	61·2 AV: 29492	52·8 AV: 6814	32·7 AV: 33183	51·5 AV: 8137	60·1 AV: 10057	0 AV: 2310
SCORE (%)	2·1 (0·9, 4·1) AV: 5234	2·0 (0·7, 5·0) AV: 6749	0·6 (0·2, 1·1) AV: 32454	0·9 (0·1, 4·2) AV: 8039	5·6 (2·4, 12·6) AV: 9054	1·8 (1·2, 2·6) AV: 2309
PCE (%)	7·3 (3·8, 13·3) AV: 4366	9·5 (3·8, 20·1) AV: 6741	2·3 (1·2, 4·1) AV: 236	3·7 (1·0, 11·0) AV: 6307	11·5 (5·1, 23·6) AV: 7769	7·8 (4·9, 12·4) AV: 1871
BMI (kg/m²)	25·3 (23·0, 28·0) AV: 29447	27·6 (24·5, 31·2) AV: 6814	24·1 (22·1, 26·5) AV: 33169	25·5 (23·1, 28·4) AV: 8048	26·7 (24·4, 29·5) AV: 9332	24·8 (22·9, 26·8) AV: 2310
Hypertension %	54·3 AV: 26700	44·9 AV: 6813	22·6 AV: 33110	34·7 AV: 6957	59·3 AV: 9501	28·5 AV: 2309
Diabetes %	3·2 AV: 24837	12·7 AV: 6790	1·1 AV: 33098	1·4 AV: 8021	10·0 AV: 9226	1·4 AV: 2310
Daily smoker %	23·8 AV: 27692	12·5 AV: 6774	45·4 AV: 32555	34·4 AV: 8109	20·4 AV: 9924	51·0 AV: 2310
Cholesterol lowering medication %	2·2 AV: 29492	16·2 AV: 6811	AV: 0	3·6 AV: 5839	13·2 AV: 9746	68·4 AV: 553
LDL cholesterol (mmol/L)	4·1 (3·5, 4·8) AV: 5241	3·2 (2·6, 3·7) AV: 6701	3·7 (3·0, 4·3) AV: 441	3·7 (2·9, 4·4) AV: 7896	3·8 (3·2, 4·4) AV: 8981	5·3 (4·4, 6·3) AV: 1871
HDL cholesterol (mmol/L)	1·3 (1·1, 1·6) AV: 5319	1·2 (1·0, 1·5) AV: 6788	1·5 (1·2, 1·7) AV: 445	1·3 (1·0, 1·6) AV: 7937	1·4 (1·1, 1·6) AV: 9087	1·3 (1·1, 1·6) AV: 1871
Non-HDL cholesterol (mmol/L)	4·7 (4·0, 5·5) AV: 5305	3·8 (3·2, 4·5) AV: 6788	4·1 (3·4, 4·9) AV: 443	4·3 (3·5, 5·1) AV: 7899	4·5 (3·8, 5·2) AV: 9086	5·6 (4·7, 6·7) AV: 1871
Non-HDL cholesterol <2·6 mmol/L %	1·2 AV: 5305	7·3 AV: 6788	4·1 AV: 443	6·1 AV: 7899	2·5 AV: 9086	0·4 AV: 1871
Non-HDL cholesterol 2·6 to <3.7 mmol/L %	16·1 AV: 5305	38·2 AV: 6788	33·2 AV: 443	27·5 AV: 7899	20·9 AV: 9086	5·8 AV: 1871
Non-HDL cholesterol 3·7 to <4.8 mmol/L %	34·4 AV: 5305	36·1 AV: 6788	35·4 AV: 443	32·1 AV: 7899	37·8 AV: 9086	22·2 AV: 1871
Non-HDL cholesterol 4·8 to <5.7 mmol/L %	27·4 AV: 5305	12·7 AV: 6788	18·7 AV: 443	20·3 AV: 7899	23·8 AV: 9086	25·4 AV: 1871
Non-HDL cholesterol ≥5·7 mmol/L %	20·8 AV: 5305	5·7 AV: 6788	8·6 AV: 443	14·1 AV: 7899	15·1 AV: 9086	46·2 AV: 1871
Incident CVD %	18·8 AV: 29484	6·9 AV: 6777	25·4 AV: 33182	8·6 AV: 8137	15·9 AV: 9919	50·1 AV: 2310
Death %	28·8 AV: 29491	7·6 AV: 6788	39·9 AV: 33182	16·9 AV: 8137	36·1 AV: 10038	88·9 AV: 2310

Supplement table S2. Weighted baseline characteristics of the non-MORGAM/non-BiomarCaRE datasets. For continuous variables median values (IQR) and for categorical variables percentages are shown. Number of individuals with available information is given for each variable (AV). SCORE = Systematic Coronary Risk Estimation; PCE = Pooled Cohort Equations; BMI = body-mass-index; non-HDL- / LDL-cholesterol = non-high density / low density lipoprotein-related cholesterol.

Table S3: Individuals on lipid lowering therapy

Survey decade	Age category	Individuals, N	Individuals on LLT, N	Individuals on LLT, %
1970-1979	<45	8,538	5	0·1
1970-1979	45-59	13,267	390	2·9
1970-1979	≥60	37	1	2·7
1980-1989	<45	41,160	8	0·02
1980-1989	45-59	66,405	281	0·4
1980-1989	≥60	26,364	333	1·3
1990-1999	<45	42,910	95	0·2
1990-1999	45-59	60,183	1,650	2·7
1990-1999	≥60	38,177	1,492	3·9
2000-2010	<45	26,023	335	1·3
2000-2010	45-59	39,521	2,697	6·8
2000-2010	≥60	37,030	5,070	13·7
2010-2013	<45	155	3	1·9
2010-2013	45-59	301	12	4·0
2010-2013	≥60	550	54	9·8

Supplement table S3. Number and percentage of individuals on lipid lowering therapy (LLT) stratified by survey decades and age categories.

Table S4: Sex-specific time dependent hazard ratios for non-HDL-cholesterol categories.

Variable	Sex	HR (95% CI) Group 1	p-value Group 1	HR (95% CI) Group 2	p-value Group 2	HR (95% CI) Group 3	p-value Group 3
**Non-HDL, 2·6 to <3·7 mmol/L**	Men	1·2 (-0·5, 0·9)	0·57	1·4 (0·0, 0·6)	0·032	1·1 (-0·1, 0·2)	0·35
**Non-HDL, 3·7 to <4·8 mmol/L**	Men	2·0 (0·0, 1·4)	0·052	2·0 (0·4, 1·0)	<0·001	1·3 (0·1, 0·4)	<0·001
**Non-HDL, 4·8 to <5·7 mmol/L**	Men	3·3 (0·5, 1·9)	<0·001	2·8 (0·8, 1·3)	<0·001	1·5 (0·3, 0·5)	<0·001
**Non-HDL, ≥ 5·7 mmol/L**	Men	6·4 (1·2, 2·6)	<0·001	4·1 (1·1, 1·7)	<0·001	1·8 (0·5, 0·7)	<0·001
**Non-HDL, 2·6 to <3·7 mmol/L**	Women	1·2 (-0·6, 0·9)	0·63	1·1 (-0·2, 0·4)	0·48	1·1 (-0·1, 0·3)	0·24
**Non-HDL, 3·7 to <4·8 mmol/L**	Women	2·1 (0·0, 1·5)	0·052	1·8 (0·3, 0·9)	<0·001	1·2 (0·0, 0·4)	0·011
**Non-HDL, 4·8 to <5·7 mmol/L**	Women	2·7 (0·1, 1·8)	0·021	2·3 (0·5, 1·1)	<0·001	1·4 (0·2, 0·5)	<0·001
**Non-HDL≥ 5·7 mmol/L**	Women	4·8 (0·7, 2·4)	<0·001	3·8 (1·0, 1·6)	<0·001	1·6 (0·3, 0·6)	<0·001

Supplement table S4. Sex-specific time dependent hazard ratios for non-HDL-cholesterol categories. The following time intervals were considered (age was used as the time scale). Group 1 attained age <45 years, Group 2 attained age between 45-59 years, and Group 3 attained age ≥60 years. The model was adjusted for age (time scale), sex and study cohort (strata), smoking, diabetes, body-mass-index, systolic blood pressure and antihypertensive medication. A non-HDL-cholesterol sex interaction was included in the model. CI = confidence interval, HR = hazard ratio.

Table S5: Sex-specific baseline characteristics for derivation and validation cohorts

	Derivation		Validation	
	**Women (N= 91,786)**	**Men (N= 107,629)**	**Women (N= 92,269)**	**Men (N= 107,162)**
**Examination years, range**	1977-2013<br>AV: 85,973	1970-2013<br>AV: 102,794	1977-2013<br>AV: 86,504	1970-2013<br>AV: 102,325
**Examination age (years)**	50·4 (40·0, 59·9)<br>AV: 91,785	51·3 (42·0, 59·6)<br>AV: 107,626	50·1 (39·2, 59·8)<br>AV: 92,267	51·3 (42·0, 59·5)<br>AV: 107,160
**Age <45 years %**	34·7<br>AV: 91,785	30·4<br>AV: 107,626	36·1<br>AV: 92,267	30·6<br>AV: 107,160
**Age 45-59 years %**	40·4<br>AV: 91,785	45·7<br>AV: 107,626	39·3<br>AV: 92,267	45·7<br>AV: 107,160
**Age ≥60 years %**	24·9<br>AV: 91,785	23·9<br>AV: 107,626	24·7<br>AV: 92,267	23·7<br>AV: 107,160
**SCORE (%)**	0·4 (0·1, 1·8)<br>AV: 79,447	1·9 (0·5, 4·9)<br>AV: 99,533	0·4 (0·1, 1·8)<br>AV: 79,853	1·8 (0·5, 5·0)<br>AV: 99,078
**PCE (%)**	2·4 (0·8, 6·8)<br>AV: 69,544	7·2 (2·6, 15·4)<br>AV: 69,119	2·4 (0·8, 6·8)<br>AV: 70,024	7·3 (2·6, 15·4)<br>AV: 68,591
**BMI (kg/m²)**	25·3 (22·6, 29·2)<br>AV: 90,826	26·0 (23·7, 28·6)<br>AV: 106,941	25·2 (22·4, 29·1)<br>AV: 91,272	26·0 (23·8, 28·7)<br>AV: 106,466
**Hypertension %**	36·5<br>AV: 89,179	43·8<br>AV: 105,355	35·8<br>AV: 89,507	43·9<br>AV: 104,900
**Diabetes %**	4·8<br>AV: 88,262	4·8<br>AV: 103,835	4·6<br>AV: 88,747	4·8<br>AV: 103,320
**Daily smoker %**	24·4<br>AV: 88,203	42·5<br>AV: 105,505	23·4<br>AV: 88,729	41·8<br>AV: 105,010
**Cholesterol lowering medication %**	4·3<br>AV: 63,987	5·5<br>AV: 59,917	4·7<br>AV: 64,335	5·3<br>AV: 59,494
**LDL cholesterol (mmol/L)**	3·5 (2·9, 4·3)<br>AV: 66,816	3·7 (3·0, 4·4)<br>AV: 65,608	3·5 (2·9, 4·3)<br>AV: 67,277	3·7 (3·0, 4·4)<br>AV: 65,298
**HDL cholesterol (mmol/L)**	1·5 (1·3, 1·8)<br>AV: 75,686	1·2 (1·0, 1·4)<br>AV: 88,590	1·5 (1·3, 1·8)<br>AV: 76,149	1·2 (1·0, 1·4)<br>AV: 88,092
**Non-HDL cholesterol (mmol/L)**	4·1 (3·3, 5·0)<br>AV: 75,637	4·5 (3·7, 5·3)<br>AV: 88,536	4·1 (3·3, 5·0)<br>AV: 76,107	4·5 (3·7, 5·3)<br>AV: 88,047
**Non-HDL cholesterol <2·6 mmol/L %**	7·1<br>AV: 75,637	3·3<br>AV: 88,536	6·9<br>AV: 76,107	3·6<br>AV: 88,047
**Non-HDL cholesterol 2·6 to <3.7 mmol/L %**	30·9<br>AV: 75,637	22·0<br>AV: 88,536	31·2<br>AV: 76,107	21·8<br>AV: 88,047
**Non-HDL cholesterol 3·7 to <4.8 mmol/L %**	31·9<br>AV: 75,637	34·6<br>AV: 88,536	31·9<br>AV: 76,107	34·4<br>AV: 88,047
**Non-HDL cholesterol 4·8 to <5.7 mmol/L %**	17·3<br>AV: 75,637	22·8<br>AV: 88,536	17·3<br>AV: 76,107	23·2<br>AV: 88,047
**Non-HDL cholesterol ≥ 5·7 mmol/L %**	12·7<br>AV: 75,637	17·2<br>AV: 88,536	12·7<br>AV: 76,107	17·0<br>AV: 88,047
**Incident CVD %**	7·8<br>AV: 90,191	16·7<br>AV: 105,946	7·9<br>AV: 90,625	16·8<br>AV: 105,554
**Death %**	14·1<br>AV: 91,144	25·5<br>AV: 106,890	13·7<br>AV: 91,630	25·8<br>AV: 106,417

Supplement table S5. Weighted baseline characteristics of the investigated study population separated for derivation and validation cohorts according to sex. For continuous variables median values (IQR) and for categorical variables percentages are shown. Number of individuals with available information is given for each variable (AV). Due to the presence of the Estonia case-cohort dataset, the summary estimates are weighted by the inverse of the inclusion probability for individuals in that cohort. The individuals from other cohorts are given weight one in the computation. From the Estonia data only the sub-cohort is used in the table computations. SCORE = Systematic Coronary Risk Estimation; PCE = Pooled Cohort Equations; BMI = body-mass-index; non-HDL- / LDL-cholesterol = non-high density / low density lipoprotein-related cholesterol; CVD = non-fatal and fatal cardiovascular disease

Table S6: Association of non-HDL-cholesterol categories with CVD – random effects

Variable	Sex	HR (95% CI)	p-value
Non-HDL-cholesterol, 2·6 to <3·7 mmol/L	Men	1·2 (1·0, 1·4)	0·04
Non-HDL-cholesterol, 3·7 to <4·8 mmol/L	Men	1·5 (1·2, 1·7)	<0·001
Non-HDL-cholesterol, 4·8 to <5·7 mmol/L	Men	1·8 (1·5, 2·2)	<0·001
Non-HDL-cholesterol, ≥5·7 mmol/L	Men	2·3 (2·0, 2·8)	<0·001
Non-HDL-cholesterol, 2·6 to <3·7 mmol/L	Women	1·1 (0·8, 1·5)	0·47
Non-HDL-cholesterol, 3·7 to <4·8 mmol/L	Women	1·4 (1·0, 1·8)	0·035
Non-HDL-cholesterol, 4·8 to <5·7 mmol/L	Women	1·6 (1·2, 2·2)	0·004
Non-HDL-cholesterol, ≥5·7 mmol/L	Women	1·9 (1·4, 2·6)	<0·001

Supplement table S6. Multivariate meta-analysis using random effects for the association of non-high density lipoprotein-related cholesterol (non-HDL-cholesterol) with incident fatal and non-fatal cardiovascular disease. Reference: non-HDL-cholesterol <2·6 mmol/L. Cox regressions were performed on each country adjusting for age, sex, study cohort, smoking, diabetes, body-mass-index, systolic blood pressure and antihypertensive medication.

Table S7: LDL- vs. non-HDL-cholesterol – C-indices for the prediction of CVD

	C-index (95% CI)	C-index difference (95% CI)	p-value
**Women**			
**Base model**	0·81 (0·81, 0·82)		
**Base model + LDL-cholesterol**	0·81 (0·81, 0·82)	0·0020 (0·0012, 0·0028)	<0·001
**Base model + non-HDL-cholesterol**	0·82 (0·81, 0·82)	0·0030 (0·0021, 0·0039)	<0·001
**Men**			
**Base model**	0·73 (0·73, 0·74)		
**Base model + LDL-cholesterol**	0·74 (0·74, 0·75)	0·0075 (0·0060, 0·0089)	<0·001
**Base model + non-HDL-cholesterol**	0·74 (0·74, 0·75)	0·0086 (0·0071, 0·0102)	<0·001

Supplement table S7. Cause specific Cox models for cardiovascular disease (CVD) and death of non-CVD causes as competing risks are used to estimate the C-index for 30-years long-term follow-up. In the models age is used as the time scale and body-mass-index, systolic blood pressure and the lipid variables are modelled using cubic splines. An interaction with sex is included for the lipid variables. Cohort and sex are used as strata. The models are further adjusted for smoking, diabetes and blood pressure medication. In the tables the p-values and C-index differences are always with respect to the first model in the table.

Table S8: Cross validation for each country

Tested country	Original data, RMSE %	Assuming a 50% lipid reduction, RMSE %	Derivation (N=)	Validation (N=)
**Denmark**	3·10	0·78	266,459	7,048
**Finland**	3·25	0·90	234,810	38,697
**Germany**	4·31	1·16	254,262	19,245
**Italy**	9·47	3·26	231,084	42,423
**United Kingdom**	3·31	1·15	252,969	20,538
**Poland**	4·33	1·39	268,850	4,657
**Spain**	8·97	2·72	268,265	5,242
**Lithuania**	2·58	0·74	269,597	3,910
**Russia**	11·98	6·58	268,125	5,382
**France**	4·80	1·53	266,412	7,095
**Sweden**	3·39	1·42	257,530	15,977
**Norway**	3·41	0·90	235,440	38,067
**USA**	4·17	1·90	234,929	38,578
**Australia**	6·10	1·75	262,853	10,654
**The Netherlands**	4·13	1·32	258,643	14,864

Supplement table S8. Root mean square error (RMSE) cross validation. Each country in turn is omitted from the derivation set and used as validation group. Given is the RMSE for the estimated probability of non-fatal or fatal cardiovascular disease by the age of 75 (original) and after 50% reduction of non-HDL-cholesterol (assuming a 50% lipid reduction) for men (France) and both sexes (all other countries). Greece and Estonia were used in the computations but not excluded in the cross-validation because of the small sample size available for the present computations.

Table S9: Risk tool – C-indices for the prediction of CVD

**Time**	**Sex**	**C-index (95% CI) Derivation**	**C-index (95% CI) Validation**
10 years	Women	0·78 (0·77, 0·80)	0·77 (0·76, 0·78)
30 years	Women	0·76 (0·76, 0·77)	0·74 (0·73, 0·75)
10 years	Men	0·73 (0·73, 0·74)	0·72 (0·71, 0·73)
30 years	Men	0·71 (0·71, 0·72)	0·67 (0·66, 0·68)

Supplement table S9. Discrimination of the models underlying the risk tool for individuals between 35 and 75 years of age. For each cause-specific model a sex-specific Weibull baseline hazard function was fitted in the derivation. The time column indicates the time used to estimate the probabilities of non-fatal or fatal cardiovascular disease.

Table S10: 30% reduction of baseline non-HDL-cholesterol

Women											
non-HDL-cholesterol category											
		<2·6 mmol/L		2·6-<3·7 mmol/L		3·7-<4·8 mmol/L		4·8-<5·7 mmol/L		≥5·7 mmol/L	
**Age category**	**Risk factors**	*NNT*	*RRR*	*NNT*	*RRR*	*NNT*	*RRR*	*NNT*	*RRR*	*NNT*	*RRR*
**<45 years**	**0-1**	46·19	0·38	29·12	0·49	19·43	0·59	13·92	0·66	9·73	0·74
	**≥2**	22·13	0·37	15·10	0·50	10·94	0·59	8·40	0·66	5·56	0·75
**45-59 years**	**0-1**	57·61	0·31	35·99	0·41	24·85	0·49	18·32	0·55	12·36	0·63
	**≥2**	29·45	0·30	19·30	0·40	14·17	0·48	10·90	0·54	7·16	0·63
**≥60 years**	**0-1**	98·12	0·20	60·23	0·29	42·10	0·36	29·55	0·42	18·84	0·50
	**≥2**	55·75	0·20	35·71	0·29	23·75	0·36	17·52	0·42	11·34	0·50

Men											
non-HDL-cholesterol category											
		<2·6 mmol/L		2·6-<3·7 mmol/L		3·7-<4·8 mmol/L		4·8-<5·7 mmol/L		≥5·7 mmol/L	
**Age category**	**Risk factors**	*NNT*	*RRR*	*NNT*	*RRR*	*NNT*	*RRR*	*NNT*	*RRR*	*NNT*	*RRR*
**<45 years**	**0-1**	22·33	0·38	13·26	0·50	8·87	0·59	6·46	0·66	4·53	0·74
	**≥2**	13·85	0·38	8·23	0·50	5·86	0·59	4·52	0·66	3·11	0·75
**45-59 years**	**0-1**	31·98	0·30	18·72	0·40	12·55	0·49	8·91	0·55	5·73	0·65
	**≥2**	18·24	0·28	10·92	0·40	7·71	0·48	5·74	0·55	3·80	0·64
**≥60 years**	**0-1**	61·45	0·21	34·91	0·29	22·86	0·36	16·21	0·42	10·33	0·49
	**≥2**	31·89	0·20	20·30	0·29	13·38	0·36	9·75	0·41	6·36	0·50

Supplement table S10. Sex- and age-specific estimated number needed to treat (NNT) to avoid one CVD event and relative risk reduction (RRR) for cardiovascular disease by 75 years of age according to age and non-high density lipoprotein-related cholesterol (non-HDL-cholesterol) categories. The model used is assuming a hypothetical 30% reduction of non-HDL-cholesterol.

Table S11: Additive CVD risk and exemplary risk reduction

Women

		**0-1 further risk factors**				**≥2 further risk factors**			
**Age**	**Non-HDL-C**	**Incidence (%)**	**Therapy (%)**	**ARR**	**RRR**	**Incidence (%)**	**Therapy (%)**	**ARR**	**RRR**
**40 years**	<2·6 mmol/L	5·4	2·5	2·9	0·54	23·4	10·8	12·6	0·54
	2·6 to <3·7 mmol/L	6·2	2·1	4·0	0·65	26·4	9·2	17·2	0·65
	3·7 to <4·8 mmol/L	7·4	1·7	5·7	0·77	31·0	7·1	24·0	0·77
	4·8 to <5·7 mmol/L	8·6	1·4	7·2	0·84	35·1	5·6	29·5	0·84
	≥5·7 mmol/L	9·5	1·1	8·4	0·88	38·7	4·7	34·0	0·88
**55 years**	<2·6 mmol/L	4·8	2·8	2·0	0·41	21·2	12·6	8·7	0·41
	2·6 to <3·7 mmol/L	5·5	2·7	2·8	0·51	23·9	11·7	12·2	0·51
	3·7 to <4·8 mmol/L	6·6	2·4	4·2	0·63	28·2	10·4	17·9	0·63
	4·8 to <5·7 mmol/L	7·6	2·2	5·4	0·71	32·0	9·3	22·7	0·71
	≥5·7 mmol/L	8·5	2·0	6·5	0·76	35·1	8·4	26·7	0·76
**65 years**	<2·6 mmol/L	3·4	2·4	1·0	0·30	16·1	11·3	4·8	0·30
	2·6 to <3·7 mmol/L	3·9	2·4	1·5	0·39	18·2	11·2	7·0	0·39
	3·7 to <4·8 mmol/L	4·7	2·4	2·3	0·49	21·6	10·9	10·7	0·49
	4·8 to <5·7 mmol/L	5·5	2·3	3·1	0·57	24·7	10·6	14·1	0·57
	≥5·7 mmol/L	6·1	2·3	3·8	0·62	27·2	10·3	17·0	0·62

Men

		**0-1 further risk factors**				**≥2 further risk factors**			
**Age**	**Non-HDL-C**	**Incidence (%)**	**Therapy (%)**	**ARR**	**RRR**	**Incidence (%)**	**Therapy (%)**	**ARR**	**RRR**
**40 years**	<2·6 mmol/L	9·6	4·4	5·2	0·54	35·0	16·1	18·9	0·54
	2·6 to <3·7 mmol/L	11·4	4·0	7·4	0·65	40·6	14·1	26·5	0·65
	3·7 to <4·8 mmol/L	14·4	3·3	11·1	0·77	49·2	11·2	38·0	0·77
	4·8 to <5·7 mmol/L	17·2	2·8	14·5	0·84	56·3	9·0	47·3	0·84
	≥5·7 mmol/L	19·7	2·4	17·3	0·88	61·5	7·4	54·1	0·88
**55 years**	<2·6 mmol/L	8·1	4·8	3·3	0·41	30·5	18·0	12·4	0·41
	2·6 to <3·7 mmol/L	9·6	4·7	4·9	0·51	35·5	17·4	18·2	0·51
	3·7 to <4·8 mmol/L	12·1	4·5	7·7	0·63	43·6	16·0	27·6	0·63
	4·8 to <5·7 mmol/L	14·6	4·2	10·4	0·71	50·3	14·6	35·8	0·71
	≥5·7 mmol/L	16·7	4·0	12·7	0·76	55·5	13·3	42·2	0·76
**65 years**	<2·6 mmol/L	5·5	3·8	1·6	0·30	22·9	16·0	6·9	0·30
	2·6 to <3·7 mmol/L	6·5	4·0	2·5	0·39	26·9	16·5	10·4	0·39
	3·7 to <4·8 mmol/L	8·3	4·2	4·1	0·49	33·5	16·9	16·6	0·49
	4·8 to <5·7 mmol/L	9·9	4·3	5·7	0·57	39·3	16·9	22·4	0·57
	≥5·7 mmol/L	11·4	4·3	7·1	0·62	44·0	16·6	27·4	0·62

Supplement table S11. Cumulative incidences at different index ages (40, 55, and 65 years of age) of non-fatal and fatal cardiovascular disease (CVD) according to non-high density lipoprotein (non-HDL)-related cholesterol (non-HDL-cholesterol) categories given for women and men with 0-1 further cardiovascular risk factor (defined as daily smoking, arterial hypertension, diabetes mellitus, and obesity) and those with ≥2 further cardiovascular risk factors. Death of non-CVD causes was used as competing risk. Absolute risk reductions (ARR) and relative risk reductions (RRR) are given for exemplary individuals within the given categories assuming a 50% reduction of non-HDL-cholesterol (therapy). These exemplary individuals were defined as follows: non-HDL-cholesterol blood level at baseline was set as 2·2 mmol/L, 3·0 mmol/L, 4·2 mmol/L, 5·2 mmol/L, and 6·0 mmol/L, respectively; 0-1 further cardiovascular risk factor: no smoking, systolic blood pressure of 120 mmHg, no antihypertensive drug therapy, no diabetes mellitus, and body mass index of 20 kg/m²; ≥2 further cardiovascular risk factor: daily smoking, systolic blood pressure of 150 mmHg without antihypertensive drug therapy, diabetes mellitus, and body mass index of 30 kg/m².

Table S12: Long-term CVD risk prediction and the benefit of lipid reduction

Women											
**non-HDL-cholesterol category**											
		**<2·6 mmol/L**		**2·6-<3·7 mmol/L**		**3·7-<4·8 mmol/L**		**4·8-<5·7 mmol/L**		**≥5·7 mmol/L**	
**Age category**	**Risk factors**	*Risk*	*Therapy*	*Risk*	*Therapy*	*Risk*	*Therapy*	*Risk*	*Therapy*	*Risk*	*Therapy*
**<45 years**	**0-1**	5·7 (5·4, 6·1)	2·6 (2·4, 2·8)	6·9 (6·7, 7·3)	2·2 (2·2, 2·3)	8·8 (8·5, 9·1)	2·0 (2·0, 2·1)	10·9 (10·6, 11·3)	1·8 (1·8, 1·9)	14·0 (13·4, 14·7)	1·5 (1·5, 1·6)
	**≥2**	12·3 (11·1, 14·0)	5·7 (5·2, 6·6)	13·4 (12·8, 14·3)	4·3 (4·1, 4·6)	15·6 (14·9, 16·6)	3·6 (3·4, 3·8)	18·1 (17·1, 19·2)	3·0 (2·9, 3·2)	24·1 (22·6, 25·8)	2·5 (2·4, 2·7)
**45-59 years**	**0-1**	5·6 (5·3, 6·0)	3·0 (2·9, 3·2)	6·7 (6·5, 7·0)	2·8 (2·7, 2·9)	8·2 (8·1, 8·5)	2·7 (2·6, 2·8)	10·0 (9·8, 10·3)	2·7 (2·6, 2·7)	12·9 (12·5, 13·3)	2·5 (2·4, 2·6)
	**≥2**	11·3 (10·5, 12·3)	6·3 (5·8, 6·8)	12·8 (12·4, 13·6)	5·4 (5·3, 5·8)	14·6 (14·2, 15·2)	4·9 (4·8, 5·1)	16·9 (16·5, 17·5)	4·6 (4·5, 4·8)	22·1 (21·4, 23·0)	4·3 (4·1, 4·4)
**≥60 years**	**0-1**	5·0 (4·5, 5·7)	3·4 (3·1, 3·9)	5·7 (5·5, 6·0)	3·2 (3·1, 3·4)	6·7 (6·5, 6·9)	3·2 (3·1, 3·3)	8·2 (7·9, 8·4)	3·3 (3·3, 3·5)	10·7 (10·3, 11·1)	3·4 (3·3, 3·5)
	**≥2**	9·1 (7·9, 10·4)	6·3 (5·5, 7·2)	9·8 (9·3, 10·5)	5·6 (5·3, 6·0)	11·9 (11·5, 12·5)	5·7 (5·5, 6·0)	13·8 (13·3, 14·4)	5·6 (5·4, 5·9)	17·7 (17·1, 18·4)	5·6 (5·4, 5·9)

Men											
**non-HDL-cholesterol category**											
		**<2·6 mmol/L**		**2·6-<3·7 mmol/L**		**3·7-<4·8 mmol/L**		**4·8-<5·7 mmol/L**		**≥5·7 mmol/L**	
**Age category**	**Risk factors**	*Risk*	*Therapy*	*Risk*	*Therapy*	*Risk*	*Therapy*	*Risk*	*Therapy*	*Risk*	*Therapy*
**<45 years**	**0-1**	11·8 (11·2, 12·4)	5·3 (5·0, 5·6)	15·0 (14·6, 15·4)	4·7 (4·5, 4·8)	19·0 (18·6, 19·4)	4·3 (4·2, 4·4)	23·4 (23·0, 23·8)	3·8 (3·8, 3·9)	29·8 (29·2, 30·5)	3·2 (3·1, 3·3)
	**≥2**	18·9 (17·5, 20·5)	8·5 (7·9, 9·3)	24·2 (23·3, 25·2)	7·6 (7·3, 7·9)	28·8 (28·1, 29·5)	6·4 (6·3, 6·6)	33·4 (32·7, 34·2)	5·5 (5·4, 5·6)	43·0 (42·0, 44·2)	4·4 (4·3, 4·6)
**45-59 years**	**0-1**	10·6 (10·0, 11·1)	5·9 (5·6, 6·2)	13·2 (12·8, 13·6)	5·6 (5·4, 5·7)	16·4 (16·1, 16·7)	5·5 (5·3, 5·6)	20·3 (20·0, 20·6)	5·3 (5·2, 5·4)	27·0 (26·5, 27·6)	4·9 (4·8, 5·0)
	**≥2**	19·3 (18·0, 20·6)	11·1 (10·4, 11·9)	23·0 (22·2, 23·7)	9·9 (9·6, 10·2)	27·0 (26·4, 27·5)	9·1 (8·9, 9·3)	31·8 (31·3, 32·4)	8·5 (8·4, 8·7)	40·9 (40·1, 41·9)	7·5 (7·4, 7·7)
**≥60 years**	**0-1**	7·8 (7·3, 8·2)	5·3 (4·9, 5·6)	9·9 (9·6, 10·2)	5·6 (5·5, 5·8)	12·3 (12·1, 12·5)	5·9 (5·8, 6·0)	14·8 (14·5, 15·1)	6·1 (5·9, 6·2)	19·6 (19·1, 20·1)	6·3 (6·2, 6·5)
	**≥2**	15·5 (14·0, 17·0)	10·7 (9·6, 11·7)	17·2 (16·6, 17·9)	9·8 (9·5, 10·2)	21·0 (20·5, 21·6)	10·1 (9·9, 10·4)	24·7 (24·2, 25·3)	10·2 (9·9, 10·4)	31·6 (30·6, 32·5)	10·1 (9·8, 10·4)

Supplement table S12. Individual risk of fatal or non-fatal cardiovascular disease (CVD) in women and men according to age-category, non-high density lipoprotein (non-HDL)-related cholesterol (non-HDL-cholesterol) category, and the number of further cardiovascular risk factors (daily smoking, arterial hypertension, diabetes mellitus, and obesity). Given are the probability of CVD by the age of 75 years (*Risk*) and the hypothetically achievable probability for CVD by the age of 75 years after 50% reduction of non-HDL-cholesterol (*Therapy*) and their 95% confidence intervals computed using 1,000 bootstrap resamples.

Online-only figures

Figure S1: Association of non-HDL-cholesterol and CVD in individuals without diabetes mellitus

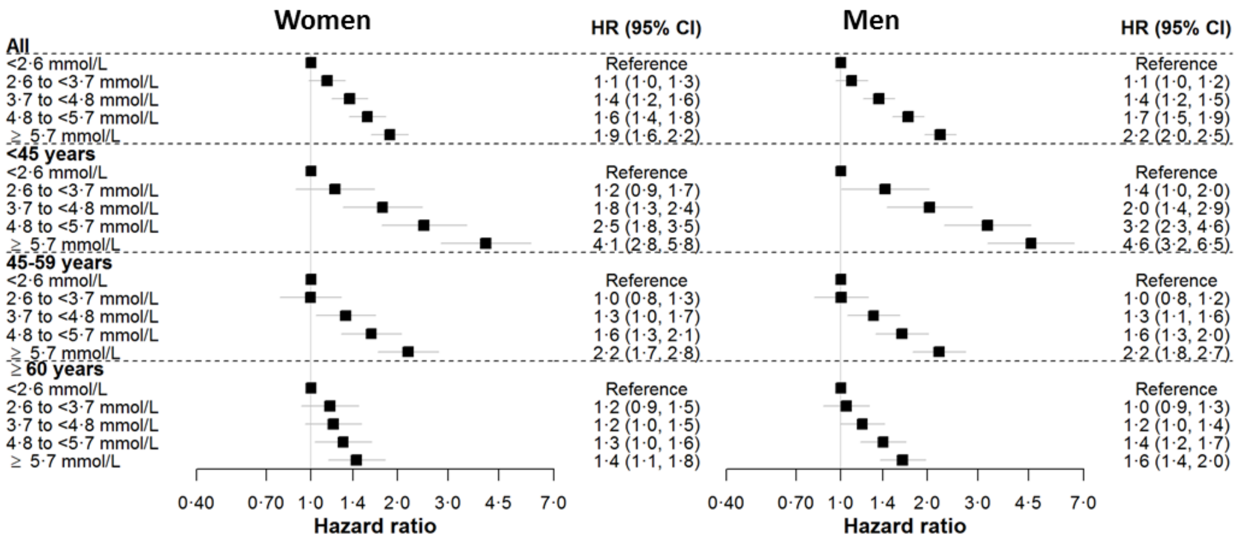

Supplement figure S1. Sex-specific hazard ratios (HR) during lifetime for fatal and non-fatal cardiovascular disease according to age categories in individuals without diabetes mellitus at baseline (reference: non-HDL-cholesterol <2·6 mmol/L). The Cox regression models were adjusted for age at baseline, sex, study cohort, smoking, diabetes, body-mass-index, systolic blood pressure and antihypertensive medication. CI = confidence interval, HR = hazard ratio.

Figure S2: Association of non-HDL-cholesterol and CVD in individuals without lipid lowering therapy

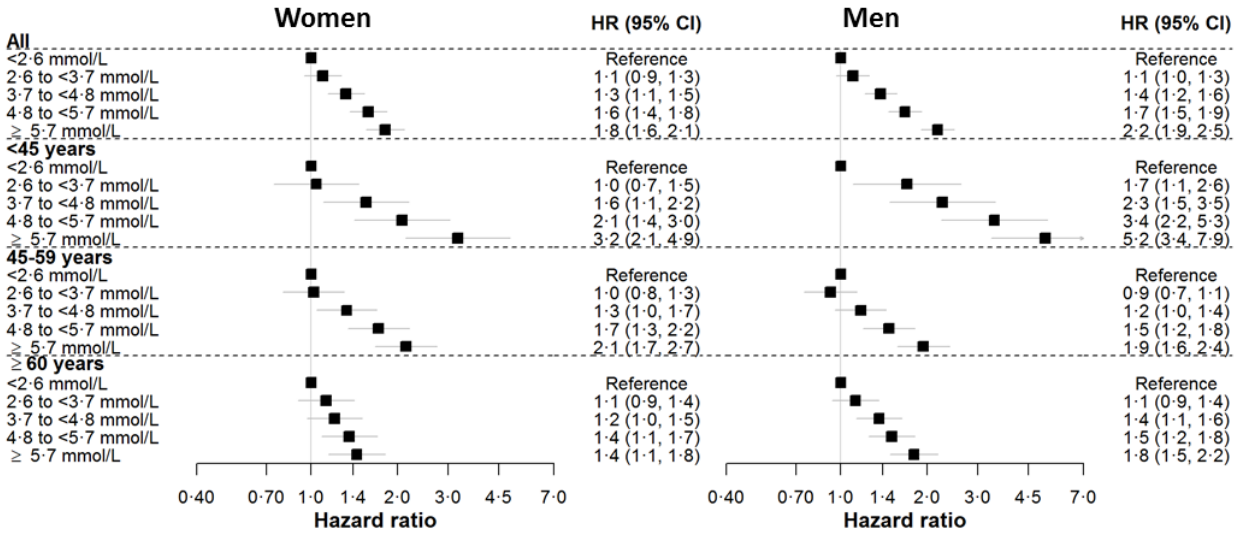

Supplement figure S2. Sex-specific hazard ratios (HR) during lifetime for fatal and non-fatal cardiovascular disease according to age categories in individuals without lipid lowering therapy at baseline (reference: non-HDL-cholesterol <2·6 mmol/L). The Cox regression models were adjusted for age at baseline, sex, study cohort, smoking, diabetes, body-mass-index, systolic blood pressure and antihypertensive medication. CI = confidence interval, HR = hazard ratio.

Figure S3: Association of non-HDL-cholesterol and CVD additionally adjusted for HDL-cholesterol

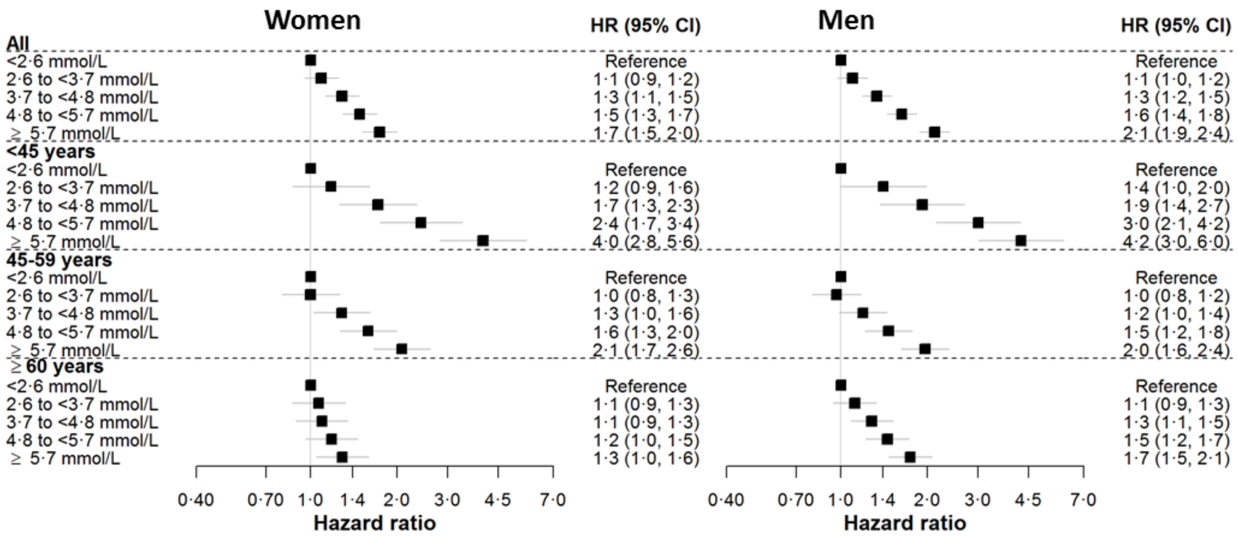

Supplement figure S3. Sex-specific hazard ratios (HR) during lifetime for fatal and non-fatal cardiovascular disease according to age categories (reference: non-HDL-cholesterol <2·6 mmol/L). The Cox regression models were adjusted for age at baseline, sex, study cohort, smoking, diabetes, body-mass-index, systolic blood pressure, antihypertensive medication, and HDL-cholesterol. CI = confidence interval, HR = hazard ratio.

Figure S4: Incidence of CVD across LDL-cholesterol thresholds

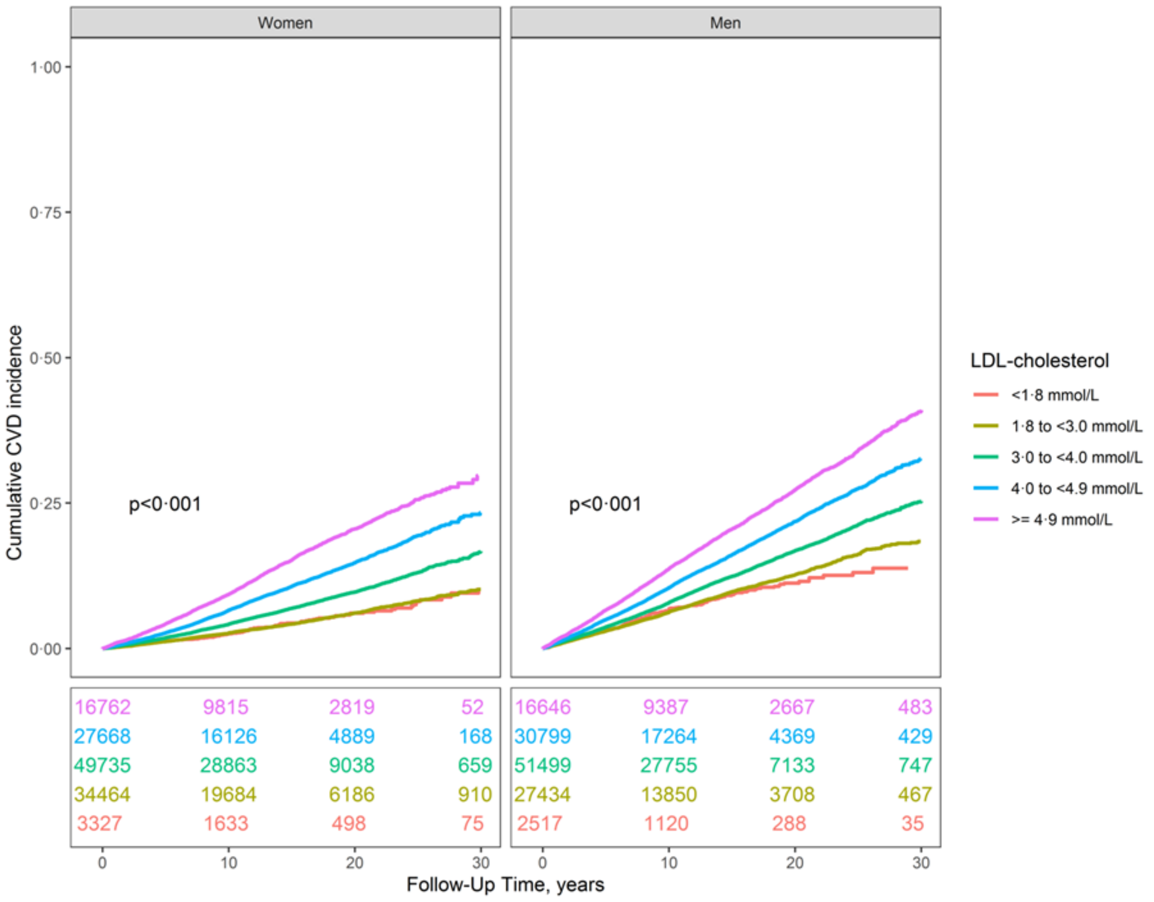

Supplement figure S4. Cumulative incidence curves and numbers at risk for incident fatal and non-fatal cardiovascular disease (CVD) according to low density lipoprotein (LDL)-related cholesterol (LDL-cholesterol) categories in women and men. Death before experiencing CVD was used as a competing risk. P-values are given for Gray's test comparing cumulative incidence curves.

Figure S5: Sex-specific continuous association of LDL-cholesterol and CVD

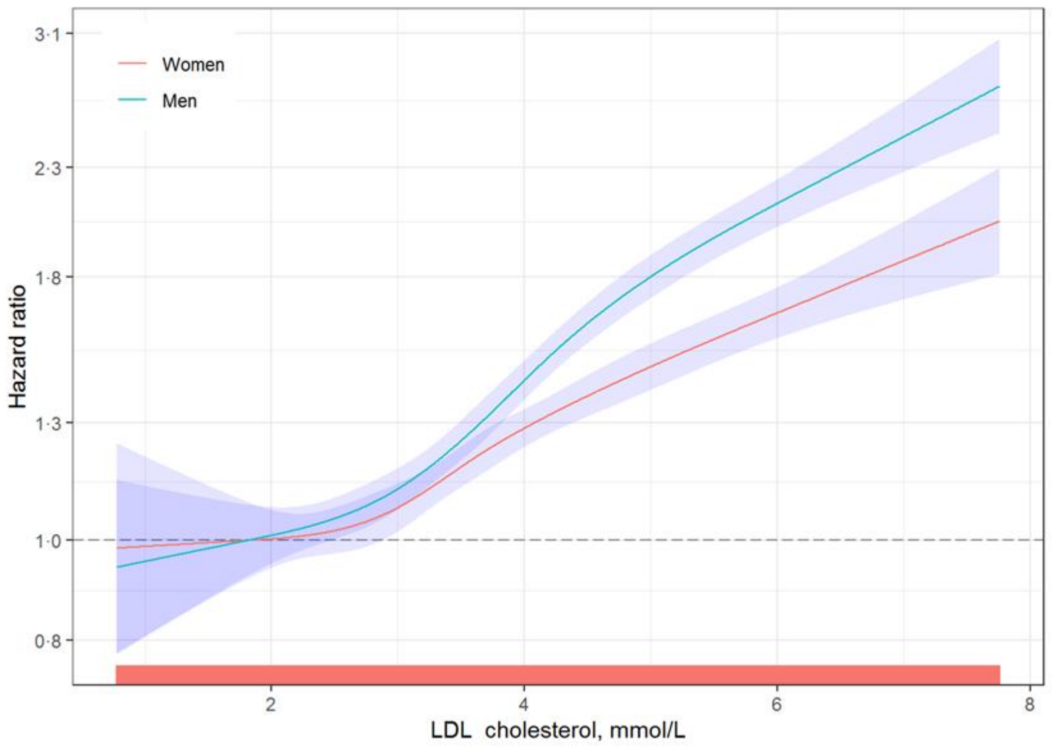

Supplement figure S5. Sex-specific linear association of LDL-cholesterol and cardiovascular disease risk (winsorized at 0·8 and 7·8 mmol/L). The Cox model used is fully adjusted for age, sex, study cohort, smoking, diabetes, body-mass-index, systolic blood pressure and antihypertensive medication. Low density lipoprotein (LDL)-related cholesterol (LDL-cholesterol) was modelled using cubic splines. A sex-LDL-cholesterol interaction was included in the model. Median follow-up was 12·8 [IQR 7·5, 18·4] years.

Figure S6: Age- and sex-specific association of LDL-cholesterol and CVD

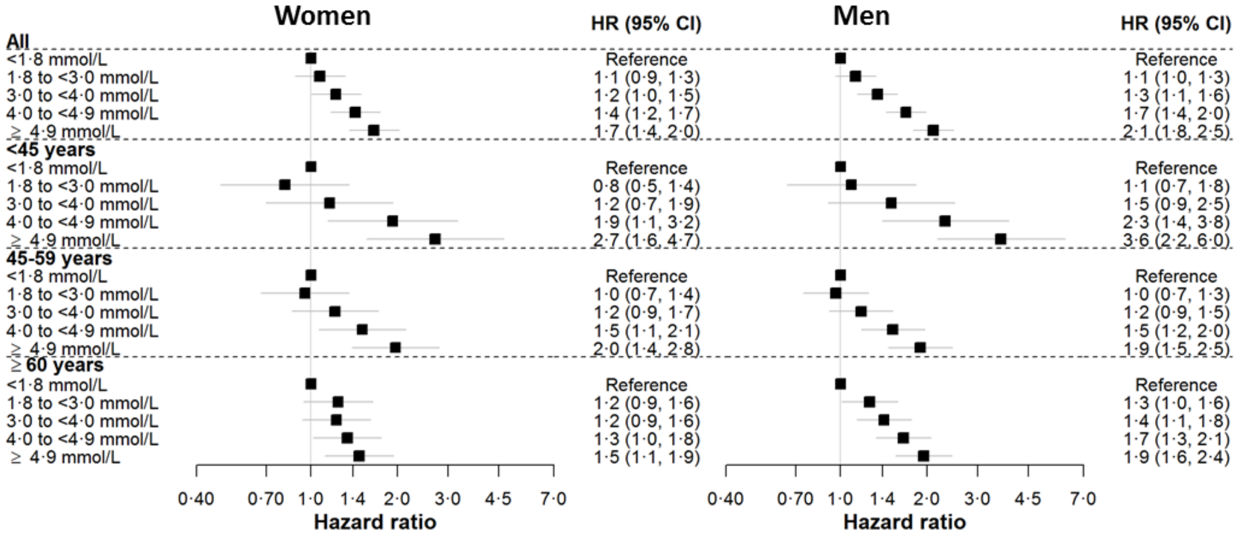

Supplement figure S6. Sex-specific hazard ratios (HR) during lifetime for fatal and non-fatal cardiovascular disease according to age categories (reference: low density lipoprotein (LDL)-related cholesterol (LDL-cholesterol) <1·8 mmol/L). The Cox regression model was adjusted for age, sex, study cohort, smoking, diabetes, body-mass-index, systolic blood pressure, and antihypertensive medication. p<0·001 for the interaction of age and LDL-cholesterol categories in women and in men. CI = confidence interval. HR = hazard ratio.

Figure S7: Diagnostic accuracy in derivation and validation datasets

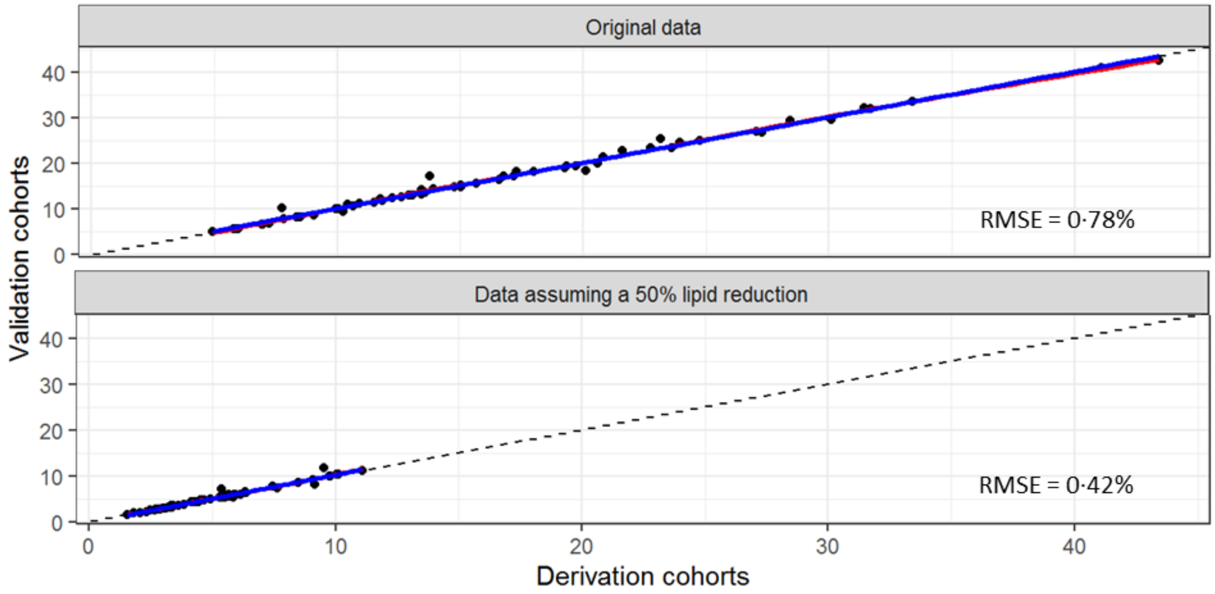

Supplemental figure S7. Comparison of derivation (x-axis) and validation cohorts (y-axis) predicted probabilities (shown as percentage) of fatal and non-fatal cardiovascular disease by the age of 75 and the hypothetically assumed probability of fatal and non-fatal cardiovascular disease by the age of 75 after 50% lipid reduction. The least squares regression lines are shown in blue and a scatterplot smoothers are shown in red. The identity line is shown as a dashed line. RMSE = root mean square error.

Figure S8. Smooth calibration curves.

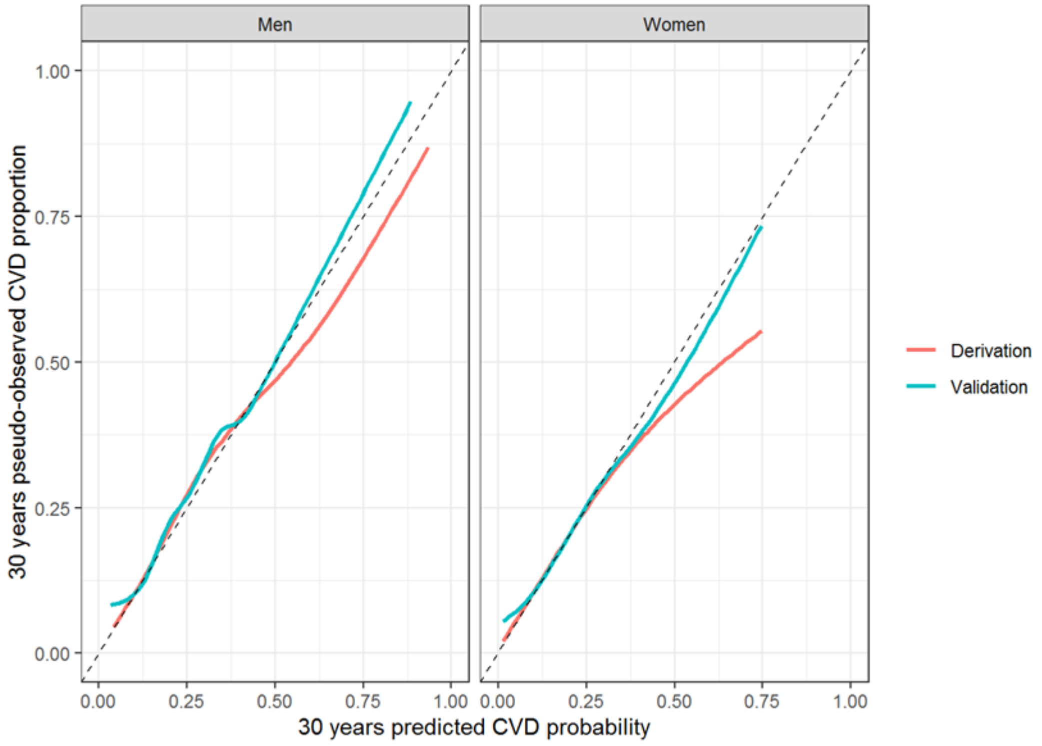

Supplement figure S8. Smooth calibration curves for predicted 30-years probabilities of cardiovascular disease (CVD) computed from the Cox cause-specific models on which the risk tool is based. In the derivation dataset 10-fold cross-validation was used. Proportion of individuals with predicted 30-years probability for CVD >50% was 2.1% (derivation) and 2.4% (validation) in women and 8.1% (derivation) and 9.9% (validation) in men. A generalized additive model was used as smoother.

Figure S9: Predicted CVD risk – time dependent hazard ratios

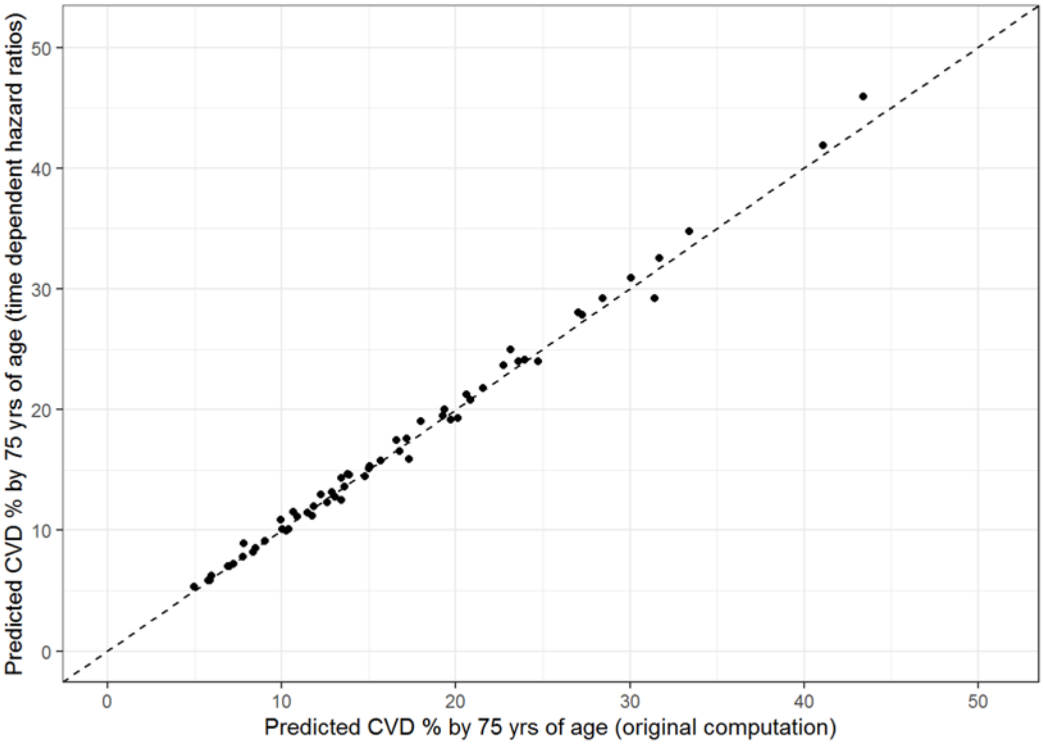

Supplement figure S9. Sensitivity analyses comparing the original cardiovascular disease (CVD) risk estimation by the age of 75 years given in the risk tool (x-axis) with an alternative risk tool based on a model using time dependent coefficients (y-axis). The identity line is shown as a dashed line.

Figure S10: Additive CVD risk and exemplary risk reduction

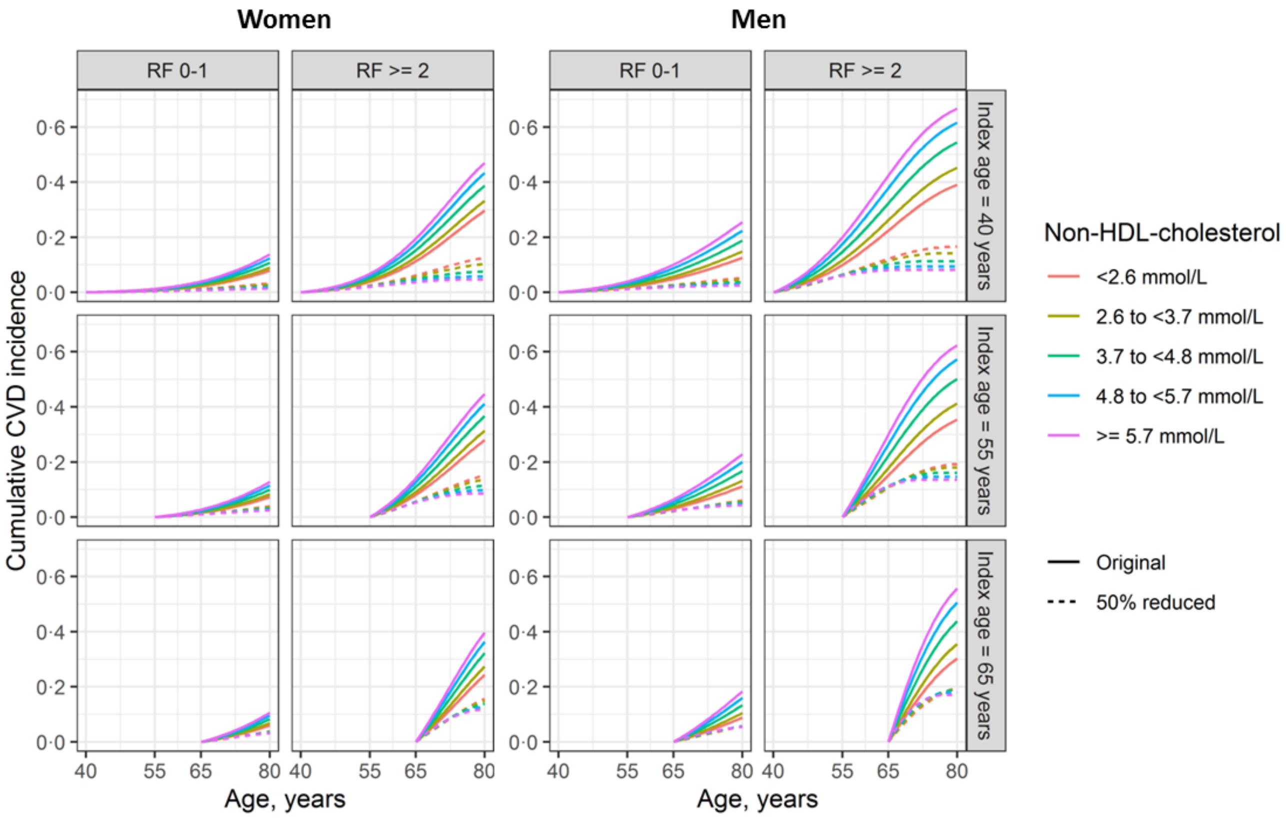

Supplemental figure S10. Cumulative incidence curves at different index ages (40, 55, and 65 years of age) of non-fatal and fatal cardiovascular disease (CVD) according to non-high density lipoprotein (non-HDL)-related cholesterol (non-HDL-cholesterol) categories given for women and men with 0-1 further cardiovascular risk factor (RF; defined as daily smoking, arterial hypertension, diabetes mellitus, and obesity) and those with ≥2 RF. Death of non-CVD causes was used as competing risk. Spotted lines are illustrating CVD incidence rates for exemplary individuals within the given categories assuming a 50% reduction of non-HDL-cholesterol. These exemplary individuals were defined as follows: non-HDL-cholesterol blood level at baseline was set as 2·2 mmol/L, 3·0 mmol/L, 4·2 mmol/L, 5·2 mmol/L, and 6·0 mmol/L, respectively; 0-1 RF: no smoking, systolic blood pressure of 120 mmHg, no antihypertensive drug therapy, no diabetes mellitus, and body mass index of 20 kg/m²; ≥2 RF: daily smoking, systolic blood pressure of 150 mmHg without antihypertensive drug therapy, diabetes mellitus, and body mass index of 30 kg/m².

Funding source of participating cohorts

The following funding sources played no part in the design of the current study; in the collection, analyses, and interpretation of data; in the writing of the report; and in the decision to submit the paper for publication:

The ATTICA study was funded by research grants from the Hellenic Society of Cardiology and the Hellenic Atherosclerosis Society.

The AusDiab study is funded by National Health and Medical Research Council (NHMRC grants 233200 and 1007544), Australian Government Department of Health and Ageing, Abbott Australasia Pty Ltd, Alphapharm Pty Ltd, Amgen Australia, AstraZeneca, Bristol-Myers Squibb, City Health Centre-Diabetes Service-Canberra, Department of Health and Community Services - Northern Territory, Department of Health and Human Services – Tasmania, Department of Health – New South Wales, Department of Health – Western Australia, Department of Health – South Australia, Department of Human Services – Victoria, Diabetes Australia, Diabetes Australia Northern Territory, Eli Lilly Australia, Estate of the Late Edward Wilson, GlaxoSmithKline, Jack Brockhoff Foundation, Janssen-Cilag, Kidney Health Australia, Marian & FH Flack Trust, Menzies Research Institute, Merck Sharp & Dohme, Novartis Pharmaceuticals, Novo Nordisk Pharmaceuticals, Pfizer Pty Ltd, Pratt Foundation, Queensland Health, Roche Diagnostics Australia, Royal Prince Alfred Hospital, Sydney, Sanofi Aventis, sanofi-synthelabo, and the Victorian Government's OIS Program.

The British Regional Heart Study is a British Heart Foundation (BHF) research group and receives support from BHF Programme grant RG/13/163/30528.

CHS was supported by contracts HHSN268201200036C, HHSN268200800007C, HHSN268201800001C, N01HC55222, N01HC85079, N01HC85080, N01HC85081, N01HC85082, N01HC85083, N01HC85086, and grants U01HL080295 and U01HL130114 from the National Heart, Lung, and Blood Institute (NHLBI), with additional contribution from the National Institute of Neurological Disorders and Stroke (NINDS). Additional support was provided by R01AG023629 from the National Institute on Aging (NIA). A full list of principal CHS investigators and institutions can be found at CHS-NHLBI.org.

The Dallas Heart Study has been supported by grants from the Donald W. Reynolds Foundation and the National Center for Advancing Translational Sciences of the NIH (UL1TR001105).

The DanMONICA cohorts at the Research Center for Prevention and Health were established over a period of ten years and have been funded by numerous sources which have been acknowledged, where appropriate, in the original articles.

The DETECT (Diabetes Cardiovascular Risk-Evaluation: Targets and Essential Data for Commitment of Treatment) study is supported by an unrestricted educational grant of Pfizer GmbH, Karlsruhe, Germany

EGCUT was supported by Estonian Research Council IUT20-60 to (A.M.) and European Union through the European Regional Development Fund 2014-2020.4.01.15-0012 GENTRANSMED (A.M).

The ESTHER study was funded by grants from the Baden-Württemberg state Ministry of Science, Research and Arts (Stuttgart, Germany), the Federal Ministry of Education and Research (Berlin, Germany), the Federal Ministry of Family Affairs, Senior Citizens, Women and Youth (Berlin, Germany), and the Saarland state Ministry for Social Affairs, Health, Women and Family Affairs (Saarbrücken, Germany).

The Framingham Heart Study (FHS) acknowledges the support of contracts NO1-HC-25195, HHSN268201500001I and 75N92019D00031 from the National Heart, Lung and Blood Institute for this research. We also acknowledge the dedication of the FHS study participants without whom this research would not be possible. Dr. Vasan is supported in part by the Evans Medical Foundation and the Jay and Louis Coffman Endowment from the Department of Medicine, Boston University School of Medicine. Dr. Benjamin is supported in part by R01HL128914; 2R01 HL092577; American Heart Association, 8SFRN34110082; 1R01 HL141434 01A1

The FINRISK surveys were mainly funded by budgetary funds of THL. Additional funding has been obtained from numerous non-profit foundations. Dr Salomaa (PI) has been supported by the Finnish Foundation for Cardiovascular Research and the Academy of Finland (grant number 139635).

The HAPIEE study was funded by the Wellcome Trust Trust (grants 064947 and 081081), the US National Institute of Aging (grant R01 AG23522) and MacArthur Foundation Research Network (grant 712058).

The Health 2000 Study is funded by the National Institute for Health and Welfare (THL), the Finnish Centre for Pensions (ETK), The Social Insurance Institution of Finland (KELA), The Local Government Pensions Institution (KEVA) and other organizations listed on the website of the survey (https://thl.fi/fi/tutkimus-ja-kehittaminen/tutkimukset-ja-hankkeet/terveys-2000-2011).

The Heinz Nixdorf RECALL Study was funded by the Heinz Nixdorf Foundation [Chairman: Martin Nixdorf; Past Chairman: Dr jur. Gerhard Schmidt (deceased)]. This study is also supported by the German Ministry of Education and Science (BMBF), and the German Aero-space Center [Deutsches Zentrum für Luft- und Raumfahrt (DLR)], Bonn, Germany. The German Research Council Assessment supported the study (DFG project: ER 155/6-2) and funded the study of psychosocial factors and neighborhood level information (DFG project SI 236/8-1 and SI 236/9-1). The sponsor of the study transferred the monitoring of the study to the German Ministry of Education and Science, Bonn, using an international advisory board and quality control as well as event committee, but had no role concerning the study design, data collection, analysis, interpretation, or writing the report.

The Nord-Trøndelag Health Study (The HUNT Study) is a collaboration between and was funded by HUNT Research Centre, (Faculty of Medicine and Health Sciences, NTNU, Norwegian University of Science and Technology), Trøndelag County Council, Central Norway Regional Health Authority, and the Norwegian Institute of Public Health.

Malmö Diet and Cancer Study and the Malmö Prevention Project were supported by the Swedish Research Council, the Swedish heart and Lung Foundation and the Knut and Alice Wallenberg Foundation.

The Melbourne Collaborative Cohort Study (MCCS) cohort recruitment was funded by VicHealth and Cancer Council Victoria. The MCCS was further augmented by Australian National Health and Medical Research Council grants 209057, 396414 and 1074383 and by infrastructure provided by Cancer Council Victoria. Cases and their vital status were ascertained through the Victorian Cancer Registry and the Australian Institute of Health and Welfare, including the National Death Index and the Australian Cancer Database.

MESA was funded by: Contracts HHSN268201500003I, N01-HC-95159, N01-HC-95160, N01-HC-95161, N01-HC-95162, N01-HC-95163, N01-HC-95164, N01-HC-95165, N01-HC-95166, N01-HC-95167, N01-HC-95168 and N01-HC-95169 from the National Heart, Lung, and Blood Institute, and by grants UL1-TR-000040 and UL1-TR-001079 from the National Center for Research Resources.

The Moli-sani was partially supported by research grants from Pfizer Foundation (Rome, Italy), the Italian Ministry of University and Research (MIUR, Rome, Italy)–Programma Triennale di Ricerca, Decreto n.1588 and Instrumentation Laboratory (Milan, Italy).

The follow-up activities for the MONICA Brianza Study were funded by the Regione Lombardia Health Administration (grants 31737/1997, 17155/2004 and 10800/2009) as part of the Osservatorio Epidemiologico Cardiovascolare Regionale Lombardo.

The MONICA/KORA study was initiated and financed by the Helmholtz Zentrum München – German Research Center for Environmental Health, which is funded by the German Federal Ministry of Education and Research (BMBF) and by the State of Bavaria.

The MORGAM-Catalonia study was supported by regional budget sources of the Institute of Health Studies, Catalan Department of Health, Barcelona, Spain. The BIOMARCARE part of the study was practically supported by the Catalan Institute of Cardiovascular Sciences (ICCC), Barcelona, Spain.

The Northern Sweden MONICA project was supported by Norrbotten and Västerbotten County Councils. Dr Söderberg has been supported by the Swedish Heart–Lung Foundation (grant numbers 20140799, 20120631, 20100635), the County Council of Västerbotten (ALF, VLL-548791) and Umeå University.

The PREVEND study was founded by the Dutch Kidney Foundation supported the infrastructure of the PREVEND program from 1997 to 2003 (Grant E.033). The University Medical Center Groningen supported the infrastructure from 2003 to 2006. Dade Behring, Ausam, Roche, and Abbott financed laboratory equipment and reagents by which various laboratory determinations could be performed. The Dutch Heart Foundation supported studies on lipid metabolism (Grant 2001-005).

The Rotterdam Study is funded by Erasmus Medical Center and Erasmus University Rotterdam, Netherlands Organization for the Health Research and Development (ZonMw), the Research Institute for Diseases in the Elderly (RIDE), the Ministry of Education, Culture and Science, the Ministry for Health, Welfare and Sports, the European

Commission (DG XII), and the Municipality of Rotterdam. The funders had no role in study design, data collection and analysis, decision to publish, or preparation of the manuscript.

The Scottish Heart Health Extended Cohort (SHHEC) study was funded by the Scottish Chief Scientist Office, British Heart Foundation and FP Fleming Trust.

SHIP is part of the research Network Community Medicine of the University Medicine Greifswald, which is supported by the German Federal State of Mecklenburg- West Pomerania.

The 3rd and 4th surveys of the Tromsø Study were funded by numerous public sources, the main being the University of Tromsø the National Screening Services, the Norwegian Research Council, the Norwegian Council on Cardiovascular Diseases, and the Norwegian Foundation for Health and Rehabilitation.

ULSAM is funded by Uppsala University and Uppsala University Hospital.

Additional funding for current analyses was provided to the University Heart Center Hamburg by the Pfizer ASPIRE Cardiovascular grant award.

Online-only references

1 The alpha-tocopherol, beta-carotene lung cancer prevention study: design, methods, participant characteristics, and compliance. The ATBC Cancer Prevention Study Group. *Ann Epidemiol* 1994; **4**: 1–10.

2 Rapola JM, Virtamo J, Korhonen P, *et al.* Validity of diagnoses of major coronary events in national registers of hospital diagnoses and deaths in Finland. *Eur J Epidemiol* 1997; **13**: 133–8.

3 Leppälä JM, Virtamo J, Heinonen OP. Validation of stroke diagnosis in the National Hospital Discharge Register and the Register of Causes of Death in Finland. *Eur J Epidemiol* 1999; **15**: 155–60.

4 Osler M, Linneberg A, Glümer C, Jørgensen T. The cohorts at the Research Centre for Prevention and Health, formerly 'The Glostrup Population Studies'. *Int J Epidemiol* 2011; **40**: 602–10.

5 Lynge E, Sandegaard JL, Rebolj M. The Danish National Patient Register. *Scand J Public Health* 2011; **39**: 30–3.

6 Leitsalu L, Haller T, Esko T, *et al.* Cohort Profile: Estonian Biobank of the Estonian Genome Center, University of Tartu. *Int J Epidemiol* 2015; **44**: 1137–47.

7 Schöttker B, Haug U, Schomburg L, *et al.* Strong associations of 25-hydroxyvitamin D concentrations with all-cause, cardiovascular, cancer, and respiratory disease mortality in a large cohort study. *Am J Clin Nutr* 2013; **97**: 782–93.

8 Borodulin K, Vartiainen E, Peltonen M, *et al.* Forty-year trends in cardiovascular risk factors in Finland. *Eur J Public Health* 2015; **25**: 539–46.

9 Wild PS, Sinning CR, Roth A, *et al.* Distribution and categorization of left ventricular measurements in the general population: results from the population-based Gutenberg Heart Study. *Circ Cardiovasc Imaging* 2010; **3**: 604–13.

10 Rastenyte D, Salomaa V, Mustaniemi H, *et al.* Comparison of trends in ischaemic heart disease between North Karelia, Finland, and Kaunas, Lithuania, from 1971 to 1987. *Br Heart J* 1992; **68**: 516–23.

11 Tunstall-Pedoe H, World Health Organization, editors. MONICA, monograph, and multimedia sourcebook: world's largest study of heart disease, stroke, risk factors, and population trends 1979-2002. Geneva: World Health Organization, 2003.

12 Löwel H, Meisinger C, Heier M, Hörmann A. The Population-Based Acute Myocardial Infarction (AMI) Registry of the MONICA/KORA Study Region of Augsburg. *Gesundheitswesen* 2005; **67**: 13–8.

13 Löwel H, Lewis M, Hörmann A, Keil U. Case finding, data quality aspects and comparability of myocardial infarction registers: results of a south German register study. *J Clin Epidemiol* 1991; **44**: 249–60.

14 Pajak A, Broda G, Abernathy JR, *et al.* Poland-US collaborative study on cardiovascular epidemiology: classification agreement between US National Cholesterol Education Program and European Atherosclerosis Society hyperlipidemia guidelines in selected Polish and US populations. *Atherosclerosis* 1992; **95**: 43–50.

15 Giampaoli S, Urbinati GC, Menotti A, Ricci G. Short term changes in cardiovascular risk factors in the Di.S.Co. Intervention Project. Research Group of the DI.S.Co. Project. *Eur J Epidemiol* 1991; **7**: 372–9.

16 Di Castelnuovo A, de Curtis A, Costanzo S, *et al.* Association of D-dimer levels with all-cause mortality in a healthy adult population: findings from the MOLI-SANI study. *Haematologica* 2013; **98**: 1476–80.

17 Gianfagna F, Veronesi G, Guasti L, *et al.* Do apolipoproteins improve coronary risk prediction in subjects with metabolic syndrome? Insights from the North Italian Brianza cohort study. *Atherosclerosis* 2014; **236**: 175–81.

18 Sans S, Paluzie G, Balañá L, Puig T, Balaguer-Vintró I. [Trends in prevalence, awareness, treatment and control of arterial hypertension between 1986 and 1996: the MONICA-Catalonia study]. *Med Clin (Barc)* 2001; **117**: 246–53.

19 Sans S, Puigdefábregas A, Paluzie G, Monterde D, Balaguer-Vintró I. Increasing trends of acute myocardial infarction in Spain: the MONICA-Catalonia Study. *Eur Heart J* 2005; **26**: 505–15.

20 Giampaoli S, Palmieri L, Capocaccia R, Pilotto L, Vanuzzo D. Estimating population-based incidence and prevalence of major coronary events. *Int J Epidemiol* 2001; **30 Suppl 1**: S5-10.

21 Dobson AJ, Alexander HM, Al-Roomi K, *et al.* Coronary events in the Hunter region of New South Wales, Australia: 1984-1986. *Acta Med Scand Suppl* 1988; **728**: 84–9.

22 Peltonen M, Lundberg V, Huhtasaari F, Asplund K. Marked improvement in survival after acute myocardial infarction in middle-aged men but not in women. The Northern Sweden MONICA study 1985-94. *J Intern Med* 2000; **247**: 579–87.

23 Nikitin YP, Shalaurova IY, Serova NV. The validation of serum thiocyanate smoking data in a population survey. *Rev Epidemiol Sante Publique* 1990; **38**: 469–72.

24 Yarnell JW. The PRIME study: classical risk factors do not explain the severalfold differences in risk of coronary heart disease between France and Northern Ireland. Prospective Epidemiological Study of Myocardial Infarction. *QJM Mon J Assoc Physicians* 1998; **91**: 667–76.

25 Tunstall-Pedoe H, Peters SAE, Woodward M, Struthers AD, Belch JJF. Twenty-Year Predictors of Peripheral Arterial Disease Compared With Coronary Heart Disease in the Scottish Heart Health Extended Cohort (SHHEC). *J Am Heart Assoc* 2017; **6**. DOI:10.1161/JAHA.117.005967.

26 Völzke H, Alte D, Schmidt CO, *et al.* Cohort profile: the study of health in Pomerania. *Int J Epidemiol* 2011; **40**: 294–307.

27 Jacobsen BK, Eggen AE, Mathiesen EB, Wilsgaard T, Njølstad I. Cohort profile: the Tromso Study. *Int J Epidemiol* 2012; **41**: 961–7.

28 Mannsverk J, Wilsgaard T, Njølstad I, *et al.* Age and gender differences in incidence and case fatality trends for myocardial infarction: a 30-year follow-up. The Tromso Study. *Eur J Prev Cardiol* 2012; **19**: 927–34.

29 Mathiesen EB, Johnsen SH, Wilsgaard T, Bønaa KH, Løchen M-L, Njølstad I. Carotid plaque area and intima-media thickness in prediction of first-ever ischemic stroke: a 10-year follow-up of 6584 men and women: the Tromsø Study. *Stroke* 2011; **42**: 972–8.

30 Rywik SL, Williams OD, Pajak A, *et al.* Incidence and correlates of hypertension in the Atherosclerosis Risk in Communities (ARIC) study and the Monitoring Trends and Determinants of Cardiovascular Disease (POL-MONICA) project. *J Hypertens* 2000; **18**: 999–1006.

31 Dunstan DW, Zimmet PZ, Welborn TA, *et al.* The Australian Diabetes, Obesity and Lifestyle Study (AusDiab)--methods and response rates. *Diabetes Res Clin Pract* 2002; **57**: 119–29.

32 The Atherosclerosis Risk in Communities (ARIC) Study: design and objectives. The ARIC investigators. *Am J Epidemiol* 1989; **129**: 687–702.

33 Pitsavos C, Panagiotakos DB, Chrysohoou C, Stefanadis C. Epidemiology of cardiovascular risk factors in Greece: aims, design and baseline characteristics of the ATTICA study. *BMC Public Health* 2003; **3**: 32.

34 Panagiotakos DB, Georgousopoulou EN, Pitsavos C, *et al.* Ten-year (2002-2012) cardiovascular disease incidence and all-cause mortality, in urban Greek population: the ATTICA Study. *Int J Cardiol* 2015; **180**: 178–84.

35 Emberson JR, Whincup PH, Lawlor DA, Montaner D, Ebrahim S. Coronary heart disease prevention in clinical practice: are patients with diabetes special? Evidence from two studies of older men and women. *Heart Br Card Soc* 2005; **91**: 451–5.

36 Fried LP, Borhani NO, Enright P, *et al.* The Cardiovascular Health Study: design and rationale. *Ann Epidemiol* 1991; **1**: 263–76.

37 Victor RG, Haley RW, Willett DL, *et al.* The Dallas Heart Study: a population-based probability sample for the multidisciplinary study of ethnic differences in cardiovascular health. *Am J Cardiol* 2004; **93**: 1473–80.

38 Wittchen H-U, Glaesmer H, März W, *et al.* Cardiovascular risk factors in primary care: methods and baseline prevalence rates--the DETECT program. *Curr Med Res Opin* 2005; **21**: 619–30.

39 Simons LA, McCallum J, Friedlander Y, Simons J, Powell I, Heller R. Dubbo study of the elderly: sociological and cardiovascular risk factors at entry. *Aust N Z J Med* 1991; **21**: 701–9.

40 Simons LA, Simons J, McCallum J, Friedlander Y. Lifestyle factors and risk of dementia: Dubbo Study of the elderly. *Med J Aust* 2006; **184**: 68–70.

41 Dawber TR, Meadors GF, Moore FE. Epidemiological approaches to heart disease: the Framingham Study. *Am J Public Health Nations Health* 1951; **41**: 279–81.

42 Peasey A, Bobak M, Kubinova R, *et al.* Determinants of cardiovascular disease and other non-communicable diseases in Central and Eastern Europe: rationale and design of the HAPIEE study. *BMC Public Health* 2006; **6**: 255.

43 Heistaro S. (eds.). Methodology report: Health 2000 survey. B26/2008. Helsinki, 2008.

44 Schmermund A, Möhlenkamp S, Stang A, *et al.* Assessment of clinically silent atherosclerotic disease and established and novel risk factors for predicting myocardial infarction and cardiac death in healthy middle-aged subjects: rationale and design of the Heinz Nixdorf RECALL Study. Risk Factors, Evaluation of Coronary Calcium and Lifestyle. *Am Heart J* 2002; **144**: 212–8.

45 Krokstad S, Langhammer A, Hveem K, *et al.* Cohort Profile: the HUNT Study, Norway. *Int J Epidemiol* 2013; **42**: 968–77.

46 Sempos CT, Bild DE, Manolio TA. Overview of the Jackson Heart Study: a study of cardiovascular diseases in African American men and women. *Am J Med Sci* 1999; **317**: 142–6.

47 Berglund G, Elmstähl S, Janzon L, Larsson SA. The Malmo Diet and Cancer Study. Design and feasibility. *J Intern Med* 1993; **233**: 45–51.

48 Rosvall M, Janzon L, Berglund G, Engström G, Hedblad B. Incident coronary events and case fatality in relation to common carotid intima-media thickness. *J Intern Med* 2005; **257**: 430–7.

49 Nilsson PM, Nilsson J-A, Berglund G. Population-attributable risk of coronary heart disease risk factors during long-term follow-up: the Malmö Preventive Project. *J Intern Med* 2006; **260**: 134–41.

50 Giles GG, English DR. The Melbourne Collaborative Cohort Study. *IARC Sci Publ* 2002; **156**: 69–70.

51 Bild DE, Bluemke DA, Burke GL, *et al.* Multi-Ethnic Study of Atherosclerosis: objectives and design. *Am J Epidemiol* 2002; **156**: 871–81.

52 Pinto-Sietsma SJ, Janssen WM, Hillege HL, Navis G, De Zeeuw D, De Jong PE. Urinary albumin excretion is associated with renal functional abnormalities in a nondiabetic population. *J Am Soc Nephrol JASN* 2000; **11**: 1882–8.

53 Corsetti JP, Gansevoort RT, Bakker SJL, Sparks CE, Vart P, Dullaart RPF. Apolipoprotein B attenuates albuminuria-associated cardiovascular disease in prevention of renal and vascular endstage disease (PREVEND) participants. *J Am Soc Nephrol JASN* 2014; **25**: 2906–15.

54 Ikram MA, Brusselle GGO, Murad SD, *et al.* The Rotterdam Study: 2018 update on objectives, design and main results. *Eur J Epidemiol* 2017; **32**: 807–50.

55 Sundström J, Risérus U, Byberg L, Zethelius B, Lithell H, Lind L. Clinical value of the metabolic syndrome for long term prediction of total and cardiovascular mortality: prospective, population based cohort study. *BMJ* 2006; **332**: 878–82.

56 Carlsson AC, Ärnlöv J, Sundström J, Michaëlsson K, Byberg L, Lind L. Physical activity, obesity and risk of cardiovascular disease in middle-aged men during a median of 30 years of follow-up. *Eur J Prev Cardiol* 2016; **23**: 359–65.

57 Kulathinal S, Niemela M, Niiranen T, *et al.* Description of MORGAM Cohorts. MORGAM Project. *E-Publ Internet* 2005; : URN:NBN:fi-fe20051214.
